# A comprehensive review of stroke-related signaling pathways and treatment in western medicine and traditional Chinese medicine

**DOI:** 10.3389/fnins.2023.1200061

**Published:** 2023-06-07

**Authors:** Binhao Chen, Weifeng Jin

**Affiliations:** ^1^The First School of Clinical Medicine, Zhejiang Chinese Medical University, Hangzhou, China; ^2^College of Pharmaceutical Science, Zhejiang Chinese Medical University, Hangzhou, China

**Keywords:** stroke, crosstalk, network disease, traditional Chinese medicine, pathway

## Abstract

This review provides insight into the complex network of signaling pathways and mechanisms involved in stroke pathophysiology. It summarizes the historical progress of stroke-related signaling pathways, identifying potential interactions between them and emphasizing that stroke is a complex network disease. Of particular interest are the Hippo signaling pathway and ferroptosis signaling pathway, which remain understudied areas of research, and are therefore a focus of the review. The involvement of multiple signaling pathways, including Sonic Hedgehog (SHH), nuclear factor erythroid 2-related factor 2 (Nrf2)/antioxidant response element (ARE), hypoxia-inducible factor-1α (HIF-1α), PI3K/AKT, JAK/STAT, and AMPK in pathophysiological mechanisms such as oxidative stress and apoptosis, highlights the complexity of stroke. The review also delves into the details of traditional Chinese medicine (TCM) therapies such as Rehmanniae and Astragalus, providing an analysis of the recent status of western medicine in the treatment of stroke and the advantages and disadvantages of TCM and western medicine in stroke treatment. The review proposes that since stroke is a network disease, TCM has the potential and advantages of a multi-target and multi-pathway mechanism of action in the treatment of stroke. Therefore, it is suggested that future research should explore more treasures of TCM and develop new therapies from the perspective of stroke as a network disease.

## 1. Introduction

Stroke is a serious medical condition that occurs when brain tissue is damaged due to cerebrovascular accidents. Different types of stroke include ischemic stroke, cerebral hemorrhage, and subarachnoid hemorrhage, which are classified based on their causes and symptoms. Ischemic stroke, the most common type, occurs when brain tissue is damaged due to a lack of oxygen and nutrients caused by thrombosis, embolism, or systemic under perfusion. In contrast, a cerebral hemorrhage occurs when a blood vessel ruptures in the brain, resulting in tissue underperfusion. About fifteen percent of cerebral hemorrhages result from ruptured blood vessels, structural abnormalities of blood vessels, or post-hypertensive small vessel abnormalities. Subarachnoid hemorrhage, which accounts for about 5% of strokes, is primarily caused by ruptured saccular aneurysms (Dirnagl et al., 1999; Donnan et al., 2008).

Stroke, along with severe conditions such as ischemic heart disease, chronic obstructive pulmonary disease, and Coronavirus Disease 2019 (COVID-19), is a significant cause of mortality worldwide. The pathophysiology of stroke comprises multiple mechanisms, including inflammatory response, oxidative stress, apoptosis, angiogenesis, and autophagy (Krupinski et al., 1994; Dirnagl et al., 1999; Allen and Bayraktutan, 2009). The Global Burden of Disease study data indicates that the number of stroke cases increased to 12.2 million in 2019, which is a substantial rise of 70.0% in comparison to the total number of stroke cases recorded in 1990 (GBD 2019 Stroke Collaborators, 2021) In low-income countries, the age-standardized mortality rate for stroke is 3.6 times higher compared to high-income countries, and stroke also poses a greater burden in low-income countries. Moreover, the incidence of stroke exhibits regional disparities, as well as variations in age and gender distribution (GBD 2019 Stroke Collaborators, 2021; Vyas et al., 2021). Stroke can be treated with a variety of interventions such as anticoagulation, antiplatelet medication, blood pressure control, lipid reduction, thrombolysis, carotid endarterectomy, and stem cell transplantation, all of which can help reduce the risk of stroke and provide some relief to patients (O’Rourke et al., 2004; Donnan et al., 2008; Barthels and Das, 2020). Research on epidemiology and preventive medicine has suggested that maintaining appropriate levels of metal elements in plasma is crucial for reducing the risk of stroke. Studies have identified high concentrations of iron, copper, and selenium in plasma as risk factors for stroke development (Alim et al., 2019; Mirończuk et al., 2021; Shi et al., 2021; Zhang et al., 2021). Further research has confirmed that stroke occurrence, progression, and prognosis can be significantly affected by cell death resulting from iron-related mechanisms (Zhou et al., 2021). Selenium (Se) therapy has been found to mitigate the detrimental effects of stroke by targeting the three primary factors involved in iron-related cell death, namely lipid peroxidation, generation of reactive oxygen species, and iron metabolism (Alim et al., 2019).

The signaling pathways associated with stroke are intricate, comprehensive, and interconnected systems. The year 2011 witnessed the emergence of a new field of study known as cyber medicine (Barabási et al., 2011). Stroke rarely results from abnormalities in the product of a single gene target, but rather is a multifaceted outcome of complex network interactions (Qin et al., 2022). The main focus of this paper is the concept of “network disease”, which seeks to provide novel insights into the exploration of better drug targets. Stroke is a complex network disease that involves multiple signaling pathways, and relying solely on single-target Western drugs may not be effective in treating it or have side effects. The benefit of TCM and its formulations lies in their ability to provide a synergistic effect on multiple pathways and targets, making them effective in acting on various signaling pathways involved in stroke (Lou et al., 2022). This paper aims to explore the molecular mechanisms of stroke-related signaling pathways, provide an overview of the historical process, development, and relationship between each signaling pathway and stroke, and highlight the intricate and interconnected relationships among them. In addition, we will review the current status of TCM in improving and treating stroke through various molecular mechanisms such as angiogenesis, inflammatory response, and oxidative stress. TCM holds great potential for pharmacological studies of network diseases such as stroke.

## 2. Historical progression of stroke’s classical signaling pathways

### 2.1. Stroke related signaling pathways: historical progress and current research

**FIGURE 1:**
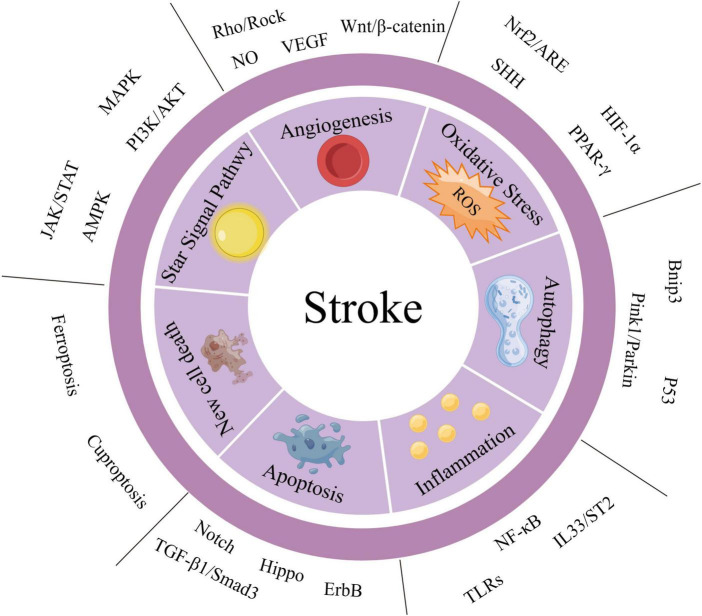
Summary diagram of stroke-related signaling pathways. We have categorized the important signaling pathways involved in stroke into two major groups: the “STAR” signaling pathway and other signaling pathways. Additionally, based on the pathophysiological mechanisms of stroke, we have divided the other signaling pathways into six aspects: angiogenesis, oxidative stress, autophagy, inflammatory response, apoptosis, and new cell death. The STAR signaling pathways include JAK/STAT, AMPK, mitogen-activated protein kinase (MAPK), and PI3K/AKT. These pathways can affect several pathophysiological mechanisms of stroke and are listed separately for emphasis.

Given the complex pathogenesis of stroke, this paper provides a macroscopic overview of its pathophysiological basis. The pathophysiological mechanisms that contribute to stroke include angiogenesis, oxidative stress, autophagy formation, inflammatory response, and apoptosis (as summarized in Supplementary Table 1). Next, we provide a summary and elaboration of stroke-related signaling pathways from various molecular mechanisms such as angiogenesis, oxidative stress, autophagy behavior, inflammatory response, cell proliferation, and apoptosis (as summarized in Supplementary Table 2). A summary diagram of stroke-related signaling pathways is shown in Figure 1. Supplementary Table 2 summarizes the historical process and research progress of signaling pathways related to different pathophysiological mechanisms of stroke. Understanding the historical development process of each signaling pathway related to stroke can enhance our comprehension of the research process and development status of a particular signaling pathway. Currently, scientific research tends to explore the correlation between two signaling pathways, as the signal axis can impact the occurrence, development, and treatment of stroke (Zhan et al., 2022). In recent years, due to the network disease characteristics of stroke, the potential crosstalk between a signaling pathway and other signaling pathways has been gradually uncovered (Lou et al., 2022).

### 2.2. From the historical process: the Hippo signaling pathway and iron death signaling pathway in stroke should be focused

Previously, we summarized Supplementary Table 2, which summarized the historical course of stroke-related signaling pathways. Since its discovery in 2017, ferroptosis, a newly identified programmed cell death (PCD) pathway, has shown a potential relationship with stroke (Dixon et al., 2012; Zille et al., 2017). The signaling pathways involved in ferroptosis mainly include lipid peroxidation, iron metabolism, and amino acid metabolism, which can affect the generation of reactive oxygen species (ROS). Ferroptosis has gained significant attention in the fields of neuroscience and medicine, and it is now considered an important area of research (Hirschhorn and Stockwell, 2019). Ferroptosis has been implicated in the development and progression of a range of diseases, including stroke. Inhibition of ferroptosis, a newly identified form of cell death, has been shown to reduce lesion damage to some extent and even improve prognosis. However, the existing research on ferroptosis, particularly in relation to stroke, is limited. Therefore, we highlight the significance of ferroptosis and draw attention to its importance in stroke-related signaling pathways.

The Hippo signaling pathway is known to play a crucial role in regulating cell behavior, growth, proliferation, and tissue homeostasis (Meng et al., 2016). The historical studies on this pathway are mainly focused on cancer, and it regulates organ regeneration and cell plasticity (Harvey et al., 2013). Regeneration is also very important for the prognosis of stroke, affecting tissue recovery and cell regeneration after stroke injury. Therefore, it is very important to study the role of this signaling pathway in stroke. Recently, the important role of Sonic Hedgehog signaling pathway in brain repair and functional recovery after stroke suggests that the regulation of Sonic Hedgehog signaling pathway is a potential strategy to extend the therapeutic window after stroke. The molecular mechanism of regulating the Hippo signaling pathway can affect ischemia-reperfusion injury (Jin et al., 2017; Gong et al., 2019, 2021). Therefore, we take it out of the historical process of stroke and emphasize the potential of this pathway in stroke with a lot of pen and ink, suggesting that new and more research should be carried out.

It is crucial to consider the aforementioned pathways, however, there is limited strong historical research on their specific mechanisms and effects in the treatment of stroke. Additionally, it has been revealed that ferroptosis has crosstalk with other pathways such as necroptosis and oxidative stres (Yan et al., 2021). Furthermore, there is a potential crosstalk between the Hippo signaling pathway and the ferroptosis signaling pathway (He et al., 2022). Therefore, in the following sections, we will provide a summary of the historical progression of the Hippo signaling pathway and the ferroptosis signaling pathway, their underlying mechanisms, the interplay between pathways, and their relationship to stroke.

### 2.3. Hippo signaling pathway and stroke: regeneration potential

#### 2.3.1. Research history of Hippo signaling pathway

**FIGURE 2:**
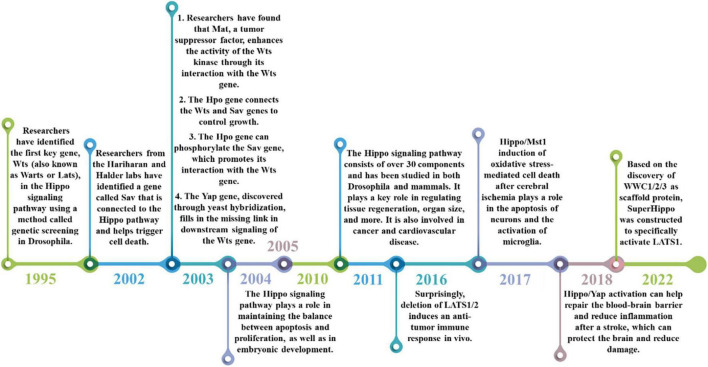
The historical process of the Hippo signaling pathway. The discovery of the Hippo signaling pathway began with the identification of the Wts gene in 1995, which was followed by the discovery of other genes such as Sav, YAP, and Mst. These findings helped to improve our understanding of the composition and function of the Hippo signaling pathway. Over the years, the Hippo signaling pathway has been found to regulate a range of biological processes, including cell proliferation, apoptosis, tissue development, and more (2004–2010). More recently, it has been implicated in cardiovascular disease, cancer (2011–2016), and its potential role in stroke treatment has been discovered (2018–2022).

The Hippo signaling pathway, also referred to as the Salvador/Warts/Hippo (SWH) pathway, has gained attention as a research area in recent years. It was initially discovered in tissues of the fruit fly Drosophila melanogaster. The research history of Hippo signaling pathway is shown in Figure 2. The first crucial gene of the Hippo signaling pathway, known as Warts (Wts or Lats), was identified through genetic screening in 1995 in Drosophila. This gene encodes a protein serine/threonine kinase and acts as a newly identified tumor suppressor gene. Inhibition of its expression can lead to excessive proliferation and abnormal differentiation (Justice et al., 1995; Xu et al., 1995). Subsequently, in 2002, the Hariharan and Halder labs identified the second gene of the Hippo pathway, Sav. This gene contains two domains associated with the Wts gene and promotes apoptosis. Mutations in the Sav gene are associated with abnormal proliferation and cancer (Kango-Singh et al., 2002; Tapon et al., 2002). Since then, there have been several discoveries in the Hippo signaling pathway. For instance, the tumor suppressor Mat was found to interact with the Wts gene to enhance Wts kinase activity (Lai et al., 2005). Additionally, the Hpo gene encodes a Ste-20 family protein that links the Wts gene with the Sav gene to control growth (Harvey et al., 2003; Udan et al., 2003; Wu et al., 2003). Among them, the Sav gene can be phosphorylated by the Hpo gene to promote the interaction with the Wts gene (Pantalacci et al., 2003). Furthermore, the Yap gene was found in yeast hybridization, which makes up for the missing link of downstream transduction of the Wts gene to regulate the cell cycle and activate cell death regulators (Huang et al., 2005). The identification of Mat, Hpo, Yap, and other genes has enabled the linkage of previously identified pathway components, resulting in the discovery of the Hippo signaling pathway, which regulates organ size by controlling cell number. The pathway was first identified in Drosophila tissues, and more than 30 components have been identified through various studies. Research has advanced from the initial studies in Drosophila to studies in mammals, with the pathway being evolutionarily conserved, as the key molecules identified in Drosophila have homologous genes in mammals (Meng et al., 2016). The Hippo signaling pathway is crucial for maintaining the balance between cell apoptosis and proliferation (Harvey et al., 2003). Furthermore, the Hippo signaling pathway is involved in embryonic development, tissue regeneration, and the regulation of organ size (Grijalva et al., 2014; Elbediwy et al., 2016; Moya and Halder, 2019). The Hippo signaling pathway has been implicated in a wide range of diseases, including cancer and cardiovascular diseases, due to its important function (Moroishi et al., 2015; Zanconato et al., 2016; Zheng and Pan, 2019).

#### 2.3.2. The key regulatory mechanisms of Hippo signaling pathway

The Hippo signaling pathway is regulated through a series of steps. In mammals, the pathway is activated by upstream membrane protein receptors in response to extracellular signals. These receptors are subsequently phosphorylated by a sequence of conserved kinases, which ultimately regulate the activity of downstream effectors, namely Yes-associated protein (YAP)/PDZ-binding motif (TAZ) (Meng et al., 2016). Upon activation, YAP/TAZ may relocate to the nucleus and engage with TEA domain family members (TEAD) 1-4, triggering the activation of downstream transcription factors and consequent transcriptional activation (Lian et al., 2010; Meng et al., 2016; Pobbati and Hong, 2020).

**FIGURE 3:**
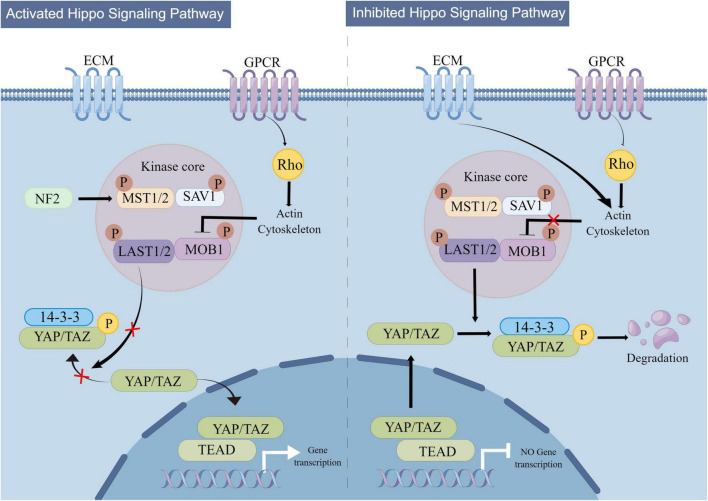
Mechanistic diagram of the Hippo signaling pathway. The Hippo signaling pathway is activated by a variety of upstream signals, such as mechanical signals from cell contacts and G protein-coupled receptor (GPCR) signaling, among others. Two key proteins, MST1/2 and LATS1/2, form the core of the kinase phosphorylation cascade in the Hippo signaling pathway. Various signals that stimulate the Hippo signaling pathway can affect the localization of YAP/TAZ within the cell, leading to changes in their binding to TEAD and regulation of downstream Hippo signaling pathway targets.

The regulatory mechanism of Hippo signaling is shown in Figure 3. The Hippo signaling pathway is stimulated by various upstream signals, including mechanical signals originating from cell contact (predominantly from the extracellular matrix or ECM), G protein-coupled receptors (GPCRs), stress signals, as well as signals linked to cell cycle, polarity, and structure (Yu et al., 2012; Meng et al., 2016; Zheng and Pan, 2019). A kinase phosphorylation cascade constitutes the core of the pathway through which these inputs are transmitted.

Two critical proteins, MST1 and its interacting protein LATS1/2, are involved in the kinase phosphorylation cascade that forms the central part of the Hippo signaling pathway (Lian et al., 2010; Meng et al., 2016). By interacting with LATS1/2, the MST1/2 protein contributes to the inhibition of cell proliferation and differentiation. The C-terminal SAV (Sav/Rassf/Hpo) domain of MST1/2, a serine/threonine kinase, can boost its activity when it forms a complex with the scaffold protein SAV1. Upon activation by specific molecules in cells, the MST1 protein forms a complex with LATS1/2, leading to the suppression of cell proliferation and differentiation and ensuring the continuity of the kinase phosphorylation cascade (Grijalva et al., 2014; Meng et al., 2016). The modulation of the MST1 and LATS1/2 proteins’ activities by the Hippo signaling pathway is pivotal in regulating cell growth and differentiation. The transcriptional coactivators Yes-associated protein (YAP) and PDZ-binding motif (TAZ) are the downstream effectors of the Hippo signaling pathway (Moya and Halder, 2019). YAP/TAZ have the ability to move back and forth between the nucleus and cytoplasm. In the absence of Hippo signaling pathway activity, YAP/TAZ relocate to the nucleus and serve as transcriptional coactivators by binding to DNA with TEAD 1-4. On the other hand, upon Hippo pathway activation, YAP/TAZ become phosphorylated and are prevented from entering the nucleus, which in turn promotes their function as transcriptional corepressors (Meng et al., 2016). The activation of the Hippo pathway leads to the suppression of YAP/TAZ function due to phosphorylation mediated by LATS1/2. Conversely, in the absence of Hippo pathway activity, YAP/TAZ become dephosphorylated and move to the nucleus, where they can engage with the transcription factors TEAD1-4 to trigger gene expression (Meng et al., 2016).

Normally, the Hippo signaling pathway proficiently manages cell growth and averts uncontrolled proliferation. Nevertheless, in specific instances, the pathway may lose its functionality, triggering abnormal growth and tumor formation. Various research studies have uncovered that the Hippo signaling pathway is frequently deactivated in tumor tissues, which facilitates cell proliferation and ultimately results in tumor advancement (Zheng and Pan, 2019). Enhancing our comprehension of the Hippo signaling pathway’s regulatory mechanisms could pave the way for innovative approaches to treat tumors (Lian et al., 2010; Elbediwy et al., 2016; Pobbati and Hong, 2020).

#### 2.3.3. Molecular mechanisms of Hippo signaling pathway in stroke

The relationship between the Hippo signaling pathway and stroke is primarily centered around the two core targets of YAP/TAZ and MST1. While previous research has primarily focused on the role of the Hippo signaling pathway in regulating cell proliferation and differentiation in cancer, there is growing interested in its potential involvement in stroke (Zanconato et al., 2016). Recently, its therapeutic potential in cardiovascular diseases has been discovered (Dey et al., 2020). Microglial activation in the infarcted area following a stroke is a critical factor that can mediate oxidative stress-induced cell death (Davalos et al., 2005). After being recognized as a crucial pro-apoptotic factor in neuronal death triggered by oxidative stress, the emerging evidence of MST1’s potential participation in ischemia-reperfusion injury implies that it could be a potential therapeutic target for the treatment of neurodegenerative disorders (Li D. et al., 2018).

Siqi Zhao et al. established a correlation between cerebral ischemia-induced microglial activation and the Hippo/MST1 signaling pathway. They discovered that Src kinase functions as an upstream factor that facilitates this association (Zhao et al., 2016). Furthermore, YAP/TAZ, which is another key site within the Hippo signaling pathway, also appears to play an important role following a stroke (Zhao et al., 2016). Activation of YAP/TAZ by dexamethasone has been shown to reduce brain damage, and infarct size, improve neurological function and decrease blood-brain barrier permeability following a stroke (Gong et al., 2019). In a study by Luping Huang et al., it was found that XMU-MP-1 could induce the nuclear localization of YAP in astrocytes, resulting in reduced brain damage, decreased release of inflammatory factors such as Interleukin-1β (IL-1β) and Interleukin-6 (IL-6), and a decrease in astrogliosis (Huang et al., 2020). Other studies have demonstrated that verteporfin, a drug used in photodynamic therapy, can reduce Blood-Brain Barrier (BBB) permeability after stroke by inhibiting the nuclear expression of YAP. This helps to maintain BBB integrity and reduce brain damage (Gong et al., 2021).

#### 2.3.4. Crosstalk between Hippo signaling and other signaling pathways

There is likely a crosstalk between the Hippo signaling pathway and other signaling pathways, such as the Wnt, Notch, and SHH pathways. Some of the most notable examples of pathway crosstalk are summarized below.

##### 2.3.4.1. Crosstalk between Hippo signaling and Wnt signaling

The regulation of cell proliferation, differentiation, migration, and apoptosis relies significantly on Wnt signaling (Varelas et al., 2010). Wnt proteins serve as signaling molecules in this pathway, and the critical proteins and receptors involved include β-catenin, Dishevelled (Dvl), and Frizzled (Fzd) (Mccrea et al., 1991; Siegfried et al., 1994; Bhanot et al., 1996). The extranuclear negative regulator YAP can limit the activity of the Wnt/β-catenin signaling pathway by interacting with Dvl, modulating Glycogen Synthase Kinase 3 beta (GSK-3β) activity, and binding to β-catenin, affecting its nuclear translocation (Varelas et al., 2010; Tsai et al., 2012; Wang Y. et al., 2017). However, upon activation of the Wnt/β-catenin signaling pathway, β-catenin can evade degradation and inhibit TAZ degradation outside the nucleus, resulting in the co-accumulation of TAZ and β-catenin (Azzolin et al., 2012). Furthermore, through binding to the DNA enhancer located in the first intron of the YAP gene, the β-catenin / Transcription Factor 4 (TCF4) complex can trigger the expression of YAP, its downstream factor, in cells (Konsavage et al., 2012; Park et al., 2015). A study published in Cell has confirmed that YAP interacts with the Wnt/β-catenin signaling pathway, involving the transcription factor Thromboxane B5 (TXB5) and the beta-transducin repeat-containing protein E3 (b-TrCP E3) ligase (Azzolin et al., 2012, 2014; Tsai et al., 2012; Park et al., 2015).

##### 2.3.4.2. Crosstalk between Hippo signaling and Notch signaling

The Notch signaling pathway is a crucial mechanism that governs cell differentiation and proliferation. This pathway holds immense significance in the field of biology, as it is responsible for regulating cell fate decisions and influencing embryonic development and stem cell differentiation (Andersson and Lendahl, 2014). By acting as a critical regulator of cellular differentiation, the Notch signaling pathway helps to ensure that cells develop into the correct types and that tissues and organs form correctly (Totaro et al., 2018). Several investigations have verified the substantial involvement of YAP1 in controlling the Notch signaling pathway in liver cancer. In particular, YAP1’s activation of Jag-1, the ligand responsible for instigating the Notch signaling cascade, has been demonstrated (Tschaharganeh et al., 2013). In addition, the conjugate of YAP and TEAD has also been found to exert regulatory effects on Notch signaling and other genes within the Notch signaling pathways (Yimlamai et al., 2014; Hansen et al., 2015).

Recent studies have shown that the activation of YAP/TAZ through mechanical cues, in conjunction with distant enhancers, can stimulate the expression of delta ligands and promote epidermal differentiation through the Notch signaling pathway. This process has a direct impact on the properties of somatic stem cells (SC), influencing their ability to differentiate and self-renew (Totaro et al., 2017). The interplay between YAP/TAZ and the Notch signaling pathway’s downstream effector is crucial in multiple biological processes, such as the development of hepatobiliary ducts, epidermis, and the pathogenesis of cancer (Totaro et al., 2018). Through its interaction with the Notch signaling pathway, YAP/TAZ influences cell fate decisions and regulates cellular proliferation, differentiation, and apoptosis, highlighting the complex interplay between different signaling pathways in various biological contexts. The emerging understanding of the role of YAP/TAZ and the Notch signaling pathway in various cellular processes underscores the need for further research into their mechanisms of action and potential therapeutic implications.

##### 2.3.4.3. Crosstalk between Hippo signaling and SHH signaling

**FIGURE 4:**
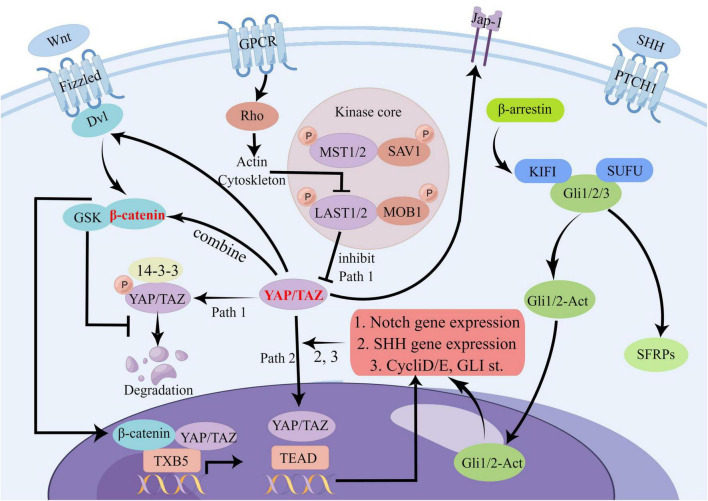
Potential crosstalk diagram between Hippo signaling pathway and Wnt, Notch, and SHH signaling pathways. In the Hippo signaling pathway, YAP/TAZ can influence several other pathways through crosstalk. For example, it can affect Dvl and β-catenin signaling in the Wnt pathway, JAP-1 receptor and downstream gene transduction in the SHH pathway, and the Notch pathway through binding to TEAD. This complex network of crosstalk represents a potential, albeit unproven, mechanism for stroke. There are many other points of crosstalk involving the Hippo signaling pathway in stroke, and we have highlighted some of the most significant ones.

The SHH signaling pathway is accountable for specifying the body axis, arranging tissues and organs, and sustaining appropriate cell proliferation in tissues. By serving as a vital modulator of embryonic development, the SHH signaling pathway helps ensure the accurate differentiation of cells and the proper formation of tissues and organs (Varjosalo and Taipale, 2008). Recent studies have demonstrated that Yes-associated protein (YAP) is a target of oncogenic activation induced by the Sonic hedgehog (SHH) pathway (Fernandez et al., 2009). In cerebellar granule neuron precursors (CGNP), SHH signaling prompts the nuclear translocation of YAP1, which stimulates their proliferation (Fernandez et al., 2009) FoxO6^–/–^ mouse studies have shown that the loss of SHH is associated with Hippo signaling (Sun et al., 2018). Furthermore, YAP has been shown to upregulate the expression of SHH, thereby contributing to bronchial morphogenesis (Isago et al., 2020). The exploration of the complex interplay between the Hippo signaling pathway and other signaling pathways in stroke remains incomplete. Our research builds upon prior studies to scrutinize plausible mechanisms of crosstalk between the Hippo signaling pathway and other pathways in the context of stroke. We provide a map below illustrating potential crosstalk between the Hippo signaling pathway and Wnt, Notch, and SHH signaling pathways (Figure 4):

### 2.4. Ferroptosis signaling pathway and stroke: regulating cell death

#### 2.4.1. Research progress on ferroptosis of new cell death

**FIGURE 5:**
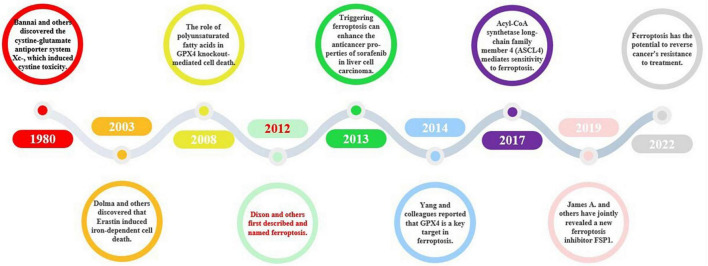
The historical course of ferroptosis. Ferroptosis, a novel form of cell death, was named in 2012, and since then, some conduits in the ferroptosis signaling pathway, such as the xC- system and GPX4, have been described. Further discoveries of key targets, such as Acyl-CoA synthetase long-chain family member 4 (ASCL4) and FSP1, have improved our understanding of the ferroptosis signaling pathway and elevated its importance. More recently, ferroptosis and its associated signaling pathways have been found to play a role in cancer, cardiovascular disease, and other fields.

In 1980, Bannai et al. made a groundbreaking discovery by identifying the antiporter protein cystine/glutamate transporter (xCT/SCL7A11), commonly known as system xC^–^ (Bannai and Kitamura, 1980). Later on, Murphy’s research revealed that system xC- also has the potential to induce glutamate toxicity, a condition that damages brain cells and can lead to neurological disorders (Hirschhorn and Stockwell, 2019). Descriptions of the unique cell death caused by cystine deprivation, which is now known as ferroptosis, existed before its official naming. These descriptions included the role of reduced glutathione (Ratan et al., 1994), ceramide-induced non-apoptotic ROS-dependent cell death (D’Autréaux and Toledano, 2007), and the involvement of polyunsaturated fatty acids in glutathione peroxidase 4 (GPX4) knockdown-mediated cell death (Seiler et al., 2008). In 2003, Dolma et al. discovered that Erastin induces iron-dependent cell death (Dolma et al., 2003). In 2012, Dixon et al. officially identified ferroptosis as a distinct mode of cell death with unique mechanisms that differentiate it from traditional apoptosis (Dixon et al., 2012). Subsequently, Yang et al. identified GPX4 as a crucial target of ferroptosis (Yang et al., 2014). Acyl-CoA synthetase long-chain family member 4 (ACSL4) is recognized as a pivotal regulator of ferroptosis and is responsible for mediating sensitivity to this process (Doll et al., 2017). In 2019, James A. Olzmann, Marcus Conrad, and Jose Pedro Friedmann Angeli identified a new repressor of ferroptosis, Ferroptosis inhibitor protein 1 (FSP1) (Doll et al., 2019). Ferroptosis is thought to have a considerable impact on numerous diseases, such as neurodegenerative diseases, cardiovascular diseases, and cancer. As a result, scientists are investigating approaches to impede ferroptosis with the aim of creating potent therapeutic interventions. The research progress on ferroptosis of new cell death is shown in Figure 5.

#### 2.4.2. Regulatory mechanisms of ferroptosis siganling pathway

Ferroptosis is an orchestrated process of cell death that encompasses various mechanisms and pathways, including iron metabolism, lipid peroxidation, and amino acid metabolism (Li J. et al., 2020; Zhang et al., 2021). The buildup of intracellular iron ions (Fe^2+^) can activate ferroptosis, underscoring its significance in the regulation of this process. Transferrin (TFRC) is a pivotal protein that facilitates the translocation of iron from extracellular to intracellular compartments and plays a crucial role in regulating iron-induced cell death (Yang and Stockwell, 2016; Li J. et al., 2020).

The cystine/glutamate antiporter xC- operates by exchanging glutamate with cystine in a 1:1 proportion. Nevertheless, excessive levels of glutamate can impede xC-’s function, inducing ferroptosis (Yang and Stockwell, 2016). Cystine is an indispensable component necessary for the biosynthesis of glutathione (GSH), a process catalyzed by glutamate-cysteine ligase (GCL) and glutathione synthetase (GSS). Nevertheless, curtailing xC-’s activity can diminish the uptake of cystine, ultimately impairing GSH synthesis (Yang and Stockwell, 2016; Zhang et al., 2021). As a consequence, the decrease in cystine uptake leads to a reduction in the activity of GPX4, an enzyme responsible for membrane lipid repair, as well as a decrease in the antioxidant capacity of cells. Ultimately, these effects promote the onset of ferroptosis (Zhang et al., 2021). Reactive Species-Generating Compound 3 (RSL3) is an influential ferroptosis elicitor that directly hinders the activity of GPX4, culminating in the diminished cellular antioxidant capability and buildup of reactive oxygen species (ROS), eventually instigating ferroptosis (Yang et al., 2014; Yang and Stockwell, 2016; Hirschhorn and Stockwell, 2019).

The quantity and distribution of polyunsaturated fatty acids (PUFAs) in a cell determine the degree of lipid oxidation and influence the occurrence of ferroptosis. Free PUFAs play a role in synthesizing lipid signaling molecules and are incorporated into membrane phospholipids. Following lipid oxidation, PUFAs transmit ferroptosis signals that induce cellular death (Hirschhorn and Stockwell, 2019; Li J. et al., 2020; Chen et al., 2021a). ACSL4 and lysophosphatidylcholine acyltransferase 3 (LPCAT3) are two critical enzymes involved in the synthesis and restructuring of PUFAs in membrane phospholipids (Doll et al., 2017; Chen et al., 2021a). These enzymes facilitate the incorporation of PUFAs into phospholipids, resulting in the formation of polyunsaturated fatty acid phospholipids (PUFA-PLs). PUFA-PLs are highly susceptible to free radical-induced oxidation, which is mediated by lipoxygenases (ALOXs). The oxidation of PUFA-PLs eventually leads to the breakdown of the lipid bilayer and disrupts membrane function, ultimately promoting ferroptosis (Hirschhorn and Stockwell, 2019; Li J. et al., 2020; Chen et al., 2021a).

#### 2.4.3. Research progress on ferroptosis signaling pathway in stroke

Ferroptosis has gained significant attention in neuroscience and medicine and is now an important area of research (Hirschhorn and Stockwell, 2019). Research has indicated that ferroptosis inhibitors possess the potential to shield against degenerative brain illnesses such as Parkinson’s disease (PD), Huntington’s disease (HD), and Alzheimer’s disease (AD), alongside other types of neurodegenerative diseases and traumatic and hemorrhagic brain injuries (Stockwell et al., 2017; Hirschhorn and Stockwell, 2019; Zhang et al., 2021). Recently, there has been growing interest in the therapeutic potential of ferroptosis in treating heart disease and cancer (Chen et al., 2021a; Li N. et al., 2021).

Ferroptosis has been suggested to be linked to stroke, as the reduced blood supply to the brain during a stroke can lead to a depletion of intracellular iron ions (Fe^2+^), which may ultimately promote ferroptosis (Zhang et al., 2021). Additionally, The accumulation of intracellular reactive oxygen species (ROS) can be caused by a stroke, which can further promote ferroptosis. While the precise role of ferroptosis in stroke remains unclear, recent studies have suggested that inhibiting ferroptosis may help reduce stroke-related damage (Zille et al., 2017; Li Q. et al., 2017).

Several studies have shown the role and therapeutic potential of ferroptosis in stroke. For instance, ZILLE, M et al. demonstrated that both ferroptosis and necrosis markers were increased following in vitro and in vivo stroke, and the inhibition of these pathways led to increased cell survival (Zille et al., 2017). In their study, Yu Cui et al. showed that protecting against cerebral ischemia-induced ferroptosis can be achieved by knocking down ACSL4, a crucial enzyme that regulates the synthesis of PUFA. Conversely, the risk of cerebral ischemia was observed to increase with the overexpression of ACSL4 (Cui et al., 2021). The inhibitory effect of baicalein on ferroptosis has been demonstrated in two models—an in vitro model of oxygen-glucose deprivation/reperfusion (OGD/R) in HT22 cells, and a rat model of transient middle cerebral artery occlusion (tMCAO) induced by RSL3. Baicalein achieves this effect primarily by regulating the expression levels of GPX4, ACSL4, and ASCL3, which are key enzymes involved in ferroptosis (Duan et al., 2021). Astragaloside IV has demonstrated potential neuroprotective effects against ferroptosis induced brain injury after subarachnoid hemorrhage (SAH) by activating the Nrf2/HO-1 signaling pathway. This pathway reduces lipid peroxidation and increases antioxidant enzyme levels, including glutathione peroxidase 4 (GPX4). As a result, the reduction of lipid peroxidation can prevent ferroptosis from occurring (Liu Z. et al., 2022). Sikai Zhan et al. showed that Danhong injection has the potential to alleviate nerve cell ferroptosis after ischemic stroke in permanent middle cerebral artery occlusion (pMCAO) mice by activating the SLC7A11/HO-1 pathway (Zhan et al., 2022). The findings of these studies indicate that the targeting of ferroptosis may hold promise as a therapeutic strategy for treating stroke in the future.

#### 2.4.4. Crosstalk between ferroptosis siganling pathway and other signaling pathways

Several cellular pathways, such as the AMPK, Wnt, and Hippo signaling pathways, may interact with ferroptosis, indicating a possibility of crosstalk between them.

##### 2.4.4.1. Crosstalk between ferroptosis siganling and Wnt signaling

Currently, the connection between ferroptosis and Wnt signaling is not yet fully understood by researchers. However, studies suggest that there could potentially be an association between Wnt signaling and cell death, indicating a possible relationship between the two pathways. Specifically, research has shown that β-catenin may bind to the TCF4 transcription factor and activate the expression of GPX4 by binding to the promoter region of GPX4. This activation can then inhibit ferroptosis (Wang H. et al., 2022).

##### 2.4.4.2. Crosstalk between ferroptosis siganling and AMPK signaling

The crosstalk between ferroptosis and the AMP-activated protein kinase (AMPK) signaling pathway has been extensively studied and established. Ferroptosis requires the phosphorylation of Beclin 1 (BECN1), and AMPK facilitates this process by directly activating the activity of BECN1. This activation leads to the initiation of autophagy, which subsequently inhibits ferroptosis by removing iron from the cell (Kang et al., 2018; Song et al., 2018). In a mouse model of renal ischemia/reperfusion injury, it was observed that activating AMPK during energy stress could reduce the pathological damage caused by ferroptosis and lower the levels of polyunsaturated fatty acids. Conversely, inactivating AMPK increased cell sensitivity to ferroptosis, suggesting the potential therapeutic significance of targeting the AMPK pathway in diseases related to ferroptosis (Lee et al., 2020; Li C. et al., 2020).

##### 2.4.4.3. Crosstalk between ferroptosis siganling and Hippo signaling

**FIGURE 6:**
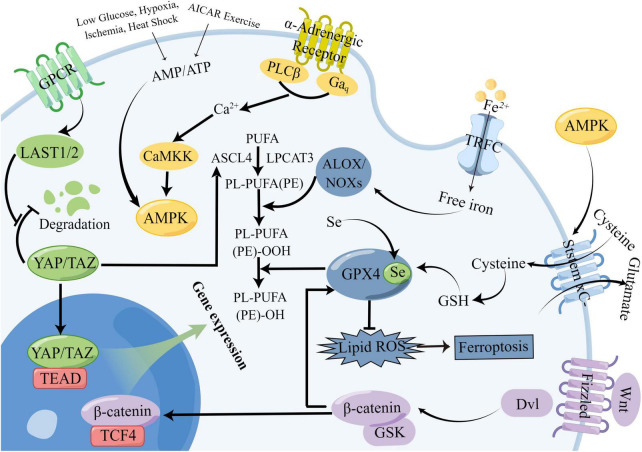
Potential crosstalk diagram between ferroptosis signaling pathway and Wnt, AMPK, and Hippo signaling pathways. The crosstalk between ferroptosis and other signaling pathways involves several components. One aspect involves β-catenin in the Wnt signaling pathway, which participates in the dialogue with ferroptosis signaling. Additionally, AMPK kinase affects glutamate transduction in ferroptosis signaling, while YAP/TAZ, key targets of the Hippo signaling pathway, impact the ASCL4 target in the ferroptosis signaling pathway.

YAP, a protein involved in the Hippo signaling pathway, plays a role in regulating the lipid peroxidation process of ferroptosis. It does so by acting on the ASCL4 target in the ferroptosis pathway, as well as through its action on NADPH Oxidase 4 (NOX4) (Yang W. H. et al., 2019; He et al., 2022). Although the intricate crosstalk mechanisms between ferroptosis and other signaling pathways in stroke have been sparsely investigated, our study delves into this area based on prior research. Our objective is to explore potential crosstalk mechanisms between ferroptosis and other signaling pathways in the context of stroke. The following is a map of potential crosstalk of signaling pathways between ferroptosis and Wnt, AMPK, and Hippo signaling pathways (Figure 6).

### 2.5. Stroke is supposed to be a network disease: a complex network of pathways

Stroke is a complex network disease. The relationship and crosstalk among the signaling pathways involved in stroke are intricate and multifaceted. Apart from the Hippo signaling pathway and ferroptosis, pathways such as Wnt, AMPK, and Notch also contribute to stroke pathology. Upon scrutinizing the interplay between ferroptosis, Hippo signaling, and stroke, it is clear that the stroke signaling pathway encompasses a complex network of signaling pathways that interact and cross-talk with each other. This phenomenon of crosstalk is also evident in the historical evolution of stroke signaling pathways (Huang et al., 2013). Individual signaling pathways in stroke are involved in multiple pathophysiologies. For example, the Rho/Rock, Wnt/β-catenin, NO, and Vascular Endothelial Growth Factor (VEGF) signaling pathways, which contribute to angiogenesis, also play a role in neurogenesis, cell proliferation, and cell apoptosis (Menet et al., 2020; Lu et al., 2021; Hu Y. et al., 2022; Wang H. et al., 2022). Similarly, the SHH signaling pathway, which is associated with oxidative stress, affects anti-oxidation, anti-apoptosis, and the promotion of neurogenesis and angiogenesis (Huang et al., 2013). The Nrf2/ARE signaling pathway, which is involved in oxidative stress, is also associated with the inflammatory response and exhibits cross-talk with the NF-κB signaling pathway (Ahmed et al., 2017). The signaling pathway of HIF-1α plays a role in stroke-related processes such as inflammatory response, angiogenesis, and neuroprotection (Cheng et al., 2014; He et al., 2021). Peroxisome proliferator-activated receptor gamma (PPAR-α) agonists have the potential to protect against excessive oxidative stress, inflammation, and apoptosis following stroke (Collino et al., 2006; Luo et al., 2006; Fong et al., 2010). The signaling pathway of NF-κB plays a role in processes related to the inflammatory and immune response, as well as apoptosis (Brand et al., 1996; Pahl, 1999). The Notch signaling pathway, commonly associated with cell apoptosis, has been found to be increasingly associated with organogenesis and angiogenesis (Campos et al., 2002; Ito et al., 2002). The Hippo signaling pathway regulates organ volume and affects tissue regeneration by controlling apoptosis (Moya and Halder, 2019). The signaling pathway of TGF-β1/Smad3 serves a dual purpose of regulating both cell proliferation and apoptosis. In addition, it also holds significant importance in several physiological processes, including but not limited to inflammation, tissue repair, and the onset of cancer (Kang et al., 2009; Gough et al., 2021). The star signaling pathways in stroke, including the PI3K/AKT, JAK/STAT, AMPK, and MAPK pathways, play a role in angiogenesis, apoptosis, inflammation, autophagy, and oxidative stress (Jiang S. et al., 2018; Shariati and Meric-Bernstam, 2019; Arnold et al., 2021; Li N. et al., 2021). These pathways have multiple downstream targets and crosstalk with other pathways, contributing to various functions in stroke. Hence, exploring the mechanisms of these signaling pathways in stroke is crucial as they hold potential for treating stroke.

Secondly, taking individual signaling pathways as examples, it is also shown that there is a complex crosstalk relationship between stroke-related signaling pathways. The PI3K/AKT signaling pathway, considered a star pathway, participates in multiple functions such as oxidative stress, apoptosis, inflammation, and angiogenesis (Shariati and Meric-Bernstam, 2019). The mechanism of action is complex and involves a wide range of signaling pathways. Moreover, there are connections and crosstalk among the PI3K/AKT signaling pathway, HIF signaling pathway, and angiogenic NO signaling pathway (Ho et al., 2012; Szabo, 2017). Additionally, activated AKT has been found to protect against oxidative damage after stroke through the Nrf2/ARE pathway (Chan, 2005). The PI3K/AKT signaling pathway activation can inhibit the expression of pro-inflammatory factors stimulated by NF-κB, thereby reducing the inflammatory response (Xian et al., 2021). Studies have demonstrated that the PI3K/AKT signaling pathway promotes VEGF production, which induces angiogenesis after a stroke. Additionally, the activation of mTOR, a downstream target of PI3K/AKT, inhibits autophagy via the PI3K/AKT/mTOR signaling pathway (Chen J. et al., 2019; Yang et al., 2021). The Nrf2 signaling pathway, involved in oxidative stress, exhibits possible crosstalk with the MAPK and PI3K/AKT signaling pathways (Alfieri et al., 2011). The Notch signaling pathway has the potential for crosstalk with various other signaling pathways, including but not limited to Wnt, TGF-β/BMP, GSK-3β, Ras/MAPK, and autophagy signaling pathways (Hansson et al., 2004; Andersson et al., 2011; Sarin and Marcel, 2017). Stroke-related signaling pathways can affect other pathways through various axes, suggesting that stroke is a network disease with complex cellular signaling mechanisms.

Finally, the complex crosstalk relationship between signaling pathways forms a network of stroke signaling pathways. For example, in stroke, taurine can reduce ferroptosis after subarachnoid hemorrhage by affecting the crosstalk between the GABA/AKT/GSK3β/β-catenin axis and the Wnt and ferroptosis signaling pathways (Liu C. et al., 2022). Additionally, artesunate can inhibit the inflammatory response after ICH through the AMPK/mTORC1/GPX4 pathway by affecting the crosstalk between the AMPK signaling pathway and the ferroptosis signaling pathway (Xie et al., 2023). However, few experimental studies have investigated the crosstalk of the Hippo signaling pathway in stroke, which is a potential area of crosstalk that requires further investigation. Meanwhile, even though the crosstalk study of other signaling pathways has not been conducted in stroke, it demonstrates the complexity of signaling pathways in the human body. For example, the PI3K/AKT signaling pathway affects stroke through various pathophysiological mechanisms, such as oxidative stress, apoptosis, inflammation, and angiogenesis, and these pathways are not affected alone but overlap with each other (Shariati and Meric-Bernstam, 2019). When western drugs are used to treat stroke, they often target a single signaling pathway, which can lead to crosstalk between multiple pathways and affect only one aspect of stroke, such as inflammatory response or angiogenesis, without addressing the various pathophysiological mechanisms of stroke (Rikitake et al., 2005; Zacharek et al., 2009). This can result in poor therapeutic outcomes and potential side effects. In contrast, TCM has the characteristics of targeting multiple pathways and can act on stroke from multiple angles (Lou et al., 2022). Therefore, in proposing that stroke should be viewed as a network disease, new therapies need to be explored. Traditional Chinese medicine has the potential to play a multi-effect role in the treatment and improvement of stroke due to its multi-target and multi-pathway action. Research has shown that Compound Tongluo Decoction can inhibit endoplasmic reticulum stress and blepharoptosis, activate the SHH signaling pathway, and promote angiogenesis. This suggests that there may be potential crosstalk between the ferroptosis signaling pathway and the SHH signaling pathway in stroke (Hui et al., 2022). Although some scholars have proposed the network disease perspective for stroke, feasible evidence is still lacking (Lehnertz et al., 2023). Therefore, we have summarized the current status, advantages, and limitations of Western and traditional Chinese medicine in treating stroke, as well as the potential for combining these two approaches. Using the network disease perspective to view stroke can facilitate the development of new therapies, the discovery of the vast potential of traditional Chinese medicine, and the exploration of new possibilities for integrating traditional Chinese and Western medicine in stroke treatment.

## 3. Recent western medicine key treatment and trials of stroke

We summarize and outline the recent mature methods of western medicine in the treatment of stroke and the methods in the research stage. The prevention and treatment of ischemic stroke remains a challenging issue in the field of neurology. Advancing our understanding of the disease’s pathogenesis, developing effective treatment methods, and discovering novel drugs hold significant economic value and practical importance. Current clinical treatment options for stroke mainly focus on thrombolysis, antiplatelet and anticoagulant therapies, lipid-lowering medications, and non-surgical treatments such as alteplase and tissue plasminogen (tPA) administration, as well as surgical interventions like craniotomy thrombectomy and ventricular drainage (Donnan et al., 2008; Powers et al., 2019). Despite this, the existing western medicine treatment options still primarily rely on thrombolysis and vascular intervention.

### 3.1. Current existing clinical treatment methods of western medicine

**FIGURE 7:**
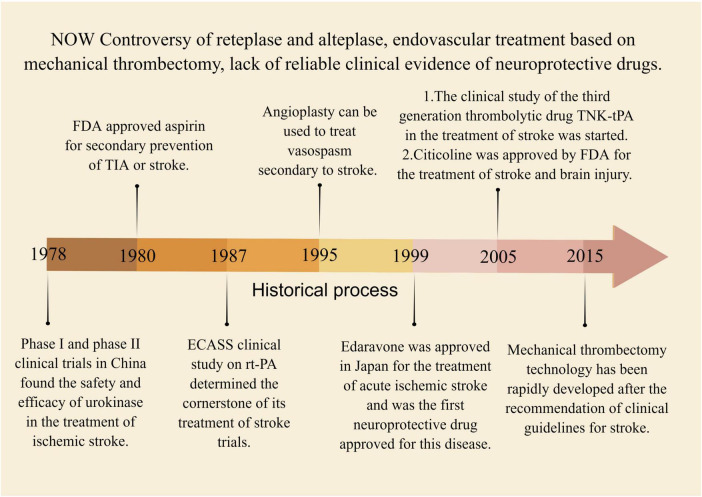
The historical process of key western medical methods in the treatment of stroke. Between 1978 and 2015, several intravenous thrombolytic therapies were discovered, including urokinase, alteplase, and tenecteplase. Concurrently, endovascular therapy emerged as a potential treatment for stroke, but lacked high-quality evidence to be confirmed by clinical trials.

Supplementary Table 3 summarizes the current clinical treatment methods for stroke, which primarily include intravenous thrombolysis, endovascular therapy, and drug therapy. Intravenous thrombolysis typically involves the use of alteplase, urokinase, and tirofiban. This method helps to reduce the incidence of stroke and increase blood perfusion in the ischemic area, but it carries a serious risk of bleeding. Additionally, the treatment time window for stroke is crucial in intravenous thrombolysis, as appropriate treatment timing plays a significant role in neurological recovery following a stroke (Wardlaw et al., 2014; Powers et al., 2019). The emergence of the third-generation thrombolytic enzyme, tirofiban, with a faster injection time and improved efficacy, poses a challenge to the primary clinical use of alteplase. However, its clinical application remains controversial due to a lack of sufficient clinical evidence to support its use (Singh et al., 2023). Mechanical thrombectomy is considered the preferred option for endovascular treatment, though the clinical effectiveness of arterial thrombectomy requires further evaluation (Hlavica et al., 2015). Drug therapy for stroke mainly consists of antiplatelet and neuroprotective medications. Aspirin and other antiplatelet drugs are limited in their efficacy due to the risk of bleeding, while the clinical effectiveness of neuroprotective drugs needs to be further evaluated in larger clinical trials (Greer, 2010; Martí-Carvajal et al., 2020). However, it is challenging to avoid the toxic side effects associated with drug therapy. For instance, edaravone is known to cause kidney and liver toxicity (Lapchak, 2010). Other treatments, such as oxygen therapy, anticoagulation, volume expansion, vascular dilation, and defibrination, have minimal evidence of effectiveness in treating stroke and are considered marginal treatments in clinical practice. Due to their limitations, these treatments are rarely used in clinical practice (Sandercock et al., 2008; Chang and Jensen, 2014; Powers et al., 2019). Figure 7 presents a summary of the historical evolution of key Western medical treatment approaches.

### 3.2. Current western drug treatment in the research and development stage

Supplementary Table 4 provides a summary of various western drugs for the treatment of stroke that are currently undergoing animal experiments or clinical trials. Among them, Fasudil, a Rock inhibitor, has shown promising results in reducing the area of cerebral infarction and is used to treat subarachnoid hemorrhage by targeting the Rho/Rock signaling pathway of angiogenesis in stroke (Rikitake et al., 2005; Shibuya et al., 2005; Shimokawa and Takeshita, 2005). Another drug, rosiglitazone (RSG), has demonstrated the ability to reduce the release of inflammatory factors and decrease the damage of recurrent stroke by activating PPAR-γ, but its clinical use is limited (Culman et al., 2007; Li et al., 2019). Metformin has demonstrated potential to reduce the risk of stroke by activating AMPK phosphorylation, suppressing NF-κB activation, and decreasing the levels of inflammatory factors such as IL-6, IL-1β, Tumor Necrosis Factor alpha (TNF-α), and Intercellular Adhesion Molecule 1 (ICAM-1) (Liu et al., 2014). Clinical drugs with specific pharmacological effects can have additional targets and sites of action, highlighting the importance of careful monitoring during clinical use to discover new applications that may improve therapeutic outcomes for patients with multiple diseases and reduce drug development costs. While Western medicine is frequently utilized for ischemic stroke treatment, it typically targets only one aspect of the disease and may have significant adverse effects. Despite the emergence of new drugs, few have been proven to be effective, and many Western drug trials focus predominantly on animal studies rather than clinical translation. Western medicine’s efficacy in treating stroke is limited, and it faces obstacles such as high research and development expenses, lengthy clinical trial periods, and restricted therapeutic benefits (Liu et al., 2018). Stroke is a multifaceted neurological disorder that involves numerous signaling pathways. As a result, Western drugs that target a single aspect of stroke have limited efficacy and often cause unwanted side effects.

## 4. Current TCM treatment improves and treats stroke through multiple targets and pathways

**FIGURE 8:**
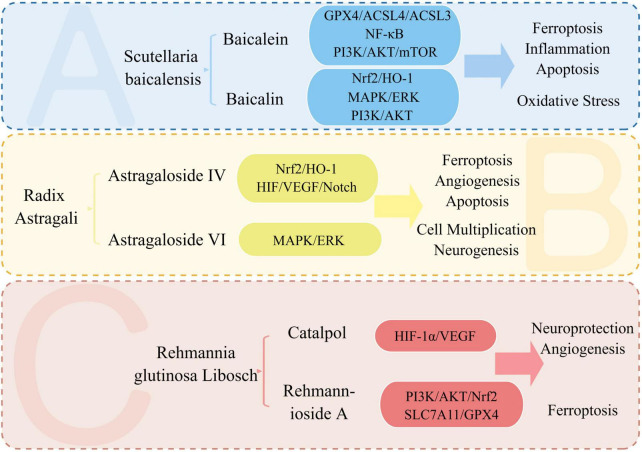
Treatment of stroke with key TCM and its active ingredients. We summarize the signaling pathways and pathophysiological mechanisms involved in the treatment of stroke with the active ingredients of Astragalus, Scutellaria, and Rehmanniae. Baicalein and baicalin, two active components, impact the pathophysiological mechanisms of stroke, including ferroptosis, oxidative stress, inflammation, and apoptosis. Meanwhile, Astragalus’ active ingredient, astragaloside IV, plays a neuroprotective role by affecting ferroptosis and angiogenesis in stroke.

Ischemic stroke, from the perspective of TCM, falls under the category of “stroke”. It is considered a syndrome of deficiency of essence and standard, where the accumulation of phlegm and blood stasis and the obstruction of brain vessels are the main pathogenesis. Therefore, promoting blood circulation and removing stasis is the main treatment approach. TCM is known for its multi-target and multi-pathway treatment approach. By acting on multiple targets of stroke signaling pathways and affecting various pathophysiological mechanisms, it can exert multi-angle treatment and improvement effects (Chen S. et al., 2022; Lou et al., 2022). Chinese patent drugs like Danhong injection and Danqi capsule have shown promising results in the prevention and treatment of ischemic stroke. Acupuncture, another TCM treatment, has also shown potential in the treatment of ischemic stroke (Liu et al., 2018; Chen S. et al., 2022). TCM is gaining wide recognition and acceptance globally (Liu et al., 2018). During the outbreak of COVID-19, TCM played a significant role in epidemic prevention, treatment, and rehabilitation. The unique benefits of TCM in preventing and treating chronic and complex multifactorial conditions, especially in cardiovascular and cerebrovascular diseases such as stroke, have gained significant attention. With a mature theoretical foundation, TCM has shown promising clinical outcomes in the prevention and treatment of these diseases (Liu et al., 2018; Zhan et al., 2022). Supplementary Table 5 summarizes TCM, and their active components for the treatment and improvement of stroke. The following provides an overview of the research status of key TCM, such as Scutellaria baicalensis, Astragalus membranaceus, Rehmanniae radix, and their active components in the treatment of stroke. The treatment of stroke with key TCM and its active ingredients is shown in Figure 8.

### 4.1. Scutellaria baicalensis

Scutellaria baicalensis, also known as Scutellaria Baicalensis, is a Chinese medicine used for clearing heat and drying dampness. According to the Compendium of Materia Medica, it is used to treat various conditions, such as wind and heat, dampness and heat, headache, heat pain of running the dolphin, asthenia of lung, fishy throat, and blood loss. The plant contains baicalin, baicalein, astragaloside iv, and other compounds that have hemostatic and fetal safety properties. Baicalein, a flavonoid with the highest content in Scutellaria baicalensis, has been found to improve blood-cerebral circulation and possess anticoagulant properties. Studies by Li, M. et al. and Yang, S. et al. have demonstrated that baicalein can inhibit ferroptosis and neuronal apoptosis, reduce cerebral infarction area, and regulate signaling pathways such as GPX4/ACSL4/ACSL3 and NF-κB (Yang S. et al., 2019; Li et al., 2022). Baicalin, another flavonoid in Scutellaria baicalensis, has anti-thrombotic and anti-inflammatory activities. Research conducted by Huang, Z. et al. and Duan, L. et al. has demonstrated that baicalin has the ability to activate the Nrf2-HO-1 signaling pathway, reduce reactive oxygen species, and inhibit ferroptosis, resulting in a reduction of brain injury (Duan et al., 2021; Huang et al., 2021a). Baicalin has also been found to activate the PI3K/AKT signaling pathway, up-regulate glutamate transporter 1, increase the release of Brain-Derived Neurotrophic Factor (BDNF) and Tropomyosin Receptor Kinase B (TrKB), and exert antioxidant, anti-inflammatory, and neuroprotective effects during OGD/R, according to studies by Zhou et al. (2017) and Li C. et al. (2020).

### 4.2. Astragalus mongholicus

”Qi is the beauty of blood, and blood is the mother of qi” is a TCM theory that emphasizes the interdependence of qi and blood. To activate blood circulation, it is necessary to first promote the movement of qi. Astragalus membranaceus, known for its qi-promoting properties, can tonify qi, discharge pus, and benefit water, which indirectly helps to promote blood circulation and remove blood stasis. Its main component is astragaloside IV. Sun et al. conducted a study where they found that Astragaloside IV promoted neurogenesis and neural stem cell proliferation after stroke in a photochemical ischemia model (Sun et al., 2020). Liang, C. et al. discovered that Astragaloside IV can significantly reduce infarct size in the MACO/R model by activating the HIF/VEGF/Notch signaling pathway, increasing miRNA-210 expression, and promoting angiogenesis and cell proliferation (Liang et al., 2020). In a study by Liu et al., it was discovered that Astragaloside IV could activate the Nrf2/HO-1 signaling pathway, which in turn increased the levels of SLC7A11, GPX4, and ROS. This activation led to an enhanced antioxidant capacity and inhibition of lipid peroxidation in an ICH model with intravascular perforation (Liu Z. et al., 2022). Chen, X. et al. discovered that Astragaloside VI has the ability to target the MAPK signaling pathway that is EGF-mediated. Through activation of the EGFR/MAPK cascade, this promotes functional repair, neurogenesis, and nerve cell proliferation in MCAO models (Chen J. et al., 2019).

### 4.3. Rehmannia glutinosa

Rehmanniae is a form of TCM available in raw and processed forms that provides benefits such as improved blood circulation, elimination of blood stagnation, and nourishing Yin while promoting body fluids. Studies by Wang, H et al. have found that Catalpol, a compound extracted from Rehmanniae, exhibits neuroprotective properties by reducing brain damage in both in vivo MCAO/R models and in vitro OGD/R models, while also encouraging the growth, movement, and formation of blood vessels in brain microvascular endothelial cells (Wang et al., 2020). Fu, Y. et al. found that Rehmannioside A, in the MCAO/R model, can reduce cognitive dysfunction, nerve damage, and suppress ferroptosis by activating the PI3K/AKT/Nrf2 and SLC7A11/GPX4 signaling pathways (Fu et al., 2022). Astragalus membranaceus, Scutellaria baicalensis, and Rehmanniae rehmanniae are important Chinese herbs that have been used to treat cardiovascular and cerebrovascular diseases. Studies have shown that the active ingredients in these herbs have multiple therapeutic effects on stroke by targeting various pathways, including angiogenesis, inflammatory response, apoptosis, and oxidative stress. The treatment of stroke with TCM and its active components involves multiple signaling pathways, including the widely-acting PI3K/AKT and ferroptosis signaling pathways. This suggests that the treatment of stroke with TCM and its active components involves overlapping effects on multiple signaling pathways, rather than targeting a single pathway.

## 5. The difference and combination of traditional Chinese and Western medicine in the treatment of stroke

In the treatment of stroke, there are differences, advantages, disadvantages, and similarities between the pharmacological mechanisms of traditional Chinese medicine and Western medicine. Integrated traditional Chinese and Western medicine is becoming more common in the treatment of stroke. It is important to continue exploring the potential of traditional Chinese medicine and discovering new possibilities for combining traditional Chinese and Western medicine to provide better treatment options for stroke patients.

Western medicine utilizes drug therapy (such as intravenous thrombolysis and antiplatelets) and surgical treatments (like mechanical thrombectomy) in the treatment of stroke. These methods can help improve blood circulation and lower blood pressure, thus reducing the risk of stroke. For example, thrombolytic agents can dissolve clots, restore blood flow, and reduce the risk of ischemia. Antihypertensive drugs can lower blood pressure and reduce the risk of brain hemorrhage. However, drug therapy may also lead to adverse reactions, such as bleeding caused by thrombolytic agents (Wardlaw et al., 2014; Powers et al., 2019). Some drugs used in the treatment of stroke require long-term use, which can lead to drug resistance and dependence. In addition, surgical treatment is also an option, which can help reduce the risk of stroke by removing vascular stenosis or repairing aneurysms. For example, aneurysm surgery can prevent rupture and thus reduce the risk of intracerebral hemorrhage. However, surgical treatment may also carry surgical risks and complications, such as postoperative infection and bleeding (Powers et al., 2019; Krishnan et al., 2021). Surgical treatment may also require a longer period of rehabilitation compared to drug therapy. Additionally, Western medicine generally follows a single-target, single-pathway approach in the treatment of stroke, such as the use of intravenous thrombolytic drugs (Powers et al., 2019).

Traditional Chinese Medicine (TCM) can treat stroke through multiple targets and pathways with less risk of adverse reactions. TCM can act on various signaling pathways, such as the PI3K/AKT and SLC7A11/GPX4 pathways, which are involved in ferroptosis, angiogenesis, and neuroprotection. This multi-target approach may lead to more comprehensive and effective treatment of stroke (Fu et al., 2022). However, due to the individualized nature of TCM intervention and the lack of feasible blinding methods, it can be difficult to conduct randomized controlled trials, leading to a limited number of high-quality clinical trials for TCM in the treatment of stroke (Yang et al., 2016; Feng et al., 2022). Despite these challenges, acupuncture, a traditional Chinese medicine treatment, has gained recognition and has been shown to effectively treat stroke (Yang et al., 2016).

In the practice of integrated traditional Chinese and Western medicine for the treatment of stroke, traditional Chinese medicine, acupuncture, massage, and other TCM therapies are often combined with conventional Western medicine and surgical methods. When used in stroke rehabilitation, the combination of TCM and Western medicine has been shown to be more effective than Western medicine alone, as it can improve neurological deficits, reduce adverse reactions, and lead to better patient outcomes. This also highlights the superior efficacy of acupuncture when combined with Western medicine (Zhong L. L. et al., 2022; Hao et al., 2023). Traditional Chinese herbal medicine (TCHM) has been used as a single or adjuvant treatment for stroke, working through a variety of mechanisms such as anti-inflammation, anti-oxidative stress, anti-apoptosis, regulation of BBB, inhibition of platelet activation, and promotion of neurogenesis and angiogenesis. TCHM provides a valuable resource for the development of therapeutic drugs to treat stroke and for the discovery of more effective and safer combination therapy or individual treatment methods (Hao et al., 2023).

## 6. Problems and prospects

A variety of signaling pathways are associated with stroke, including angiogenesis (such as the Rho/Rock, Wnt/β-catenin, and NO signaling pathways), oxidative stress (including the Nrf2/ARE and SHH signaling pathways), immune inflammation (such as the NF-κB and TLRs signaling pathways), autophagy (including the Bnip3 signaling pathways), apoptosis (including the Notch and Hippo signaling pathways), ferroptosis, cuproptosis, and others such as the PI3K/AKT, MAPK, AMPK, and JAK/STAT signaling pathways. All of these pathways are interconnected, creating a complex network of signaling pathways involved in the pathophysiology of stroke. Stroke is a complex network disease that involves a diverse range of pathophysiological mechanisms and signaling pathways. As a result, investigating stroke requires thorough examination of various cross-talk issues.

Western medicines typically treat stroke through a single target, but due to differences in genotype and phenotype among patients, there can be poor effectiveness and significant variation in the treatment’s effects. Moreover, some drugs can cause liver damage, renal toxicity, and other side effects (O’Rourke et al., 2004). Currently, thrombolytic therapy and intravascular therapy, such asrecombinant tissue plasminogen activato (rt-PA), are still the most widely recognized treatments for stroke, often combined with auxiliary measures that have a broad range of indications (Herpich and Rincon, 2020). However, the bleeding risk to patients and the time window for thrombolysis are significant issues that cannot be ignored. Therefore, stroke centers have been established, and basic and complete pre-hospital management of stroke has been introduced in many countries (Barthels and Das, 2020). The opening of “stroke green channels” and the use of internet technology to assist with hospital admissions have also been implemented. However, these measures can be challenging to implement in developing countries, such as those in Asia and Africa, where there is limited investment and insufficient technical support. Therefore, reducing the onset symptoms of patients and extending the time window for treatment could provide significant support for the diagnosis, treatment, and prognosis of stroke (Powers et al., 2019). Due to the network nature of stroke, Western drugs that target a single pathway or target may have limitations and potential side effects.

TCM compounds or preparations and their effective ingredients have the advantage of targeting multiple pathways and targets simultaneously, including PI3K/AKT, NF-κB, and iron death pathways, and regulating various pathophysiological mechanisms such as apoptosis, oxidative stress, and inflammation in stroke (Liu et al., 2018). However, the mechanism of action of TCM remains unclear and involves effects that are multi-component, involve multiple pathways, and target multiple aspects, which is a bottleneck for the modernization and internationalization of TCM (Liu et al., 2018; Zhu et al., 2022). To overcome this, modern scientific and technological means such as network pharmacology, metabolomics, and proteomics can be used to study the effect and mechanism of TCM in stroke treatment (Kibble et al., 2015). Researchers need to clarify the signaling pathway network of stroke, identify the active ingredients in TCM, and strengthen pharmacodynamic and pharmacokinetic studies to clarify their pharmacological and toxic effects. Compound Chinese medicines have shown neuroprotective and damage-reducing effects on stroke in animal experiments, but their regional promotion is limited to clinical trials in China. Therefore, it is crucial to develop these compound Chinese medicines and promote them globally (Liu et al., 2018; Lou et al., 2022). TCM has shown to have fewer side effects and can be widely promoted in developing countries, especially in Asia and Africa, for the prevention and treatment of stroke. Moreover, TCM can also be used to assist the diagnosis, treatment, and recovery of stroke in developed countries (Sarfo et al., 2023). It is crucial to develop TCM suitable for stroke treatment, as it can be used as a means to extend the time window of stroke, retard the advancement of the ailment, and improve the prognosis and rehabilitation (Liu et al., 2018; Zhu et al., 2022). Therefore, the multi-target and multi-pathway treatment approach of TCM aligns with the complex network nature of stroke as a disease.

Network pharmacology has emerged for drug target discovery, experimental design, mechanism study, and efficacy evaluation in the exploration of TCM treatment strategies for stroke (Kibble et al., 2015; Zheng et al., 2022). It is a necessary, conditional, directional, and innovative approach. However, while network pharmacology is a hot topic, the credibility and professionalism of the network pharmacology databases need to be improved, and some network pharmacology articles remain limited to data mining. Although network pharmacology combined with experimental verification is reliable for TCM treatment of stroke, research on TCM in stroke models is still limited to preliminary experimental research and lacks clinical translation (Zheng et al., 2022).

In summary, stroke is a complex disease involved with numerous signal pathways, which makes it difficult to treat. Some western drugs like tissue plasminase (tPA) and aspirin have been used clinically, they have limited effects and side effects. TCM possesses significant potential in the treatment of stroke and is a valuable resource in this regard. However, stroke treatment research has been mostly limited to animal experiments and lacks clinical transformation (Liu et al., 2018; Powers et al., 2019; Zhu et al., 2022). It is essential to explore the potential therapy of integrated traditional Chinese and western medicine in the treatment of stroke. By combining the advantages of both approaches, this therapy can complement each other’s shortcomings, add high-quality evidence, reduce adverse reactions, and increase drug efficacy. TCM is a vast treasure trove, and there are many potential drugs that can be developed from it. Therefore, in the future, efforts should be directed towards excavating and developing the TCM treasure trove (Duan T. et al., 2022; Feng et al., 2022; Zhong L. L. et al., 2022; Hao et al., 2023). Additionally, it is essential to focus on the development of TCM placebo and randomized controlled trials to improve the credibility of TCM for stroke treatment. Network pharmacology and metabolomics can be used to mine data and conduct theoretical research in combination with Chinese traditional medical codes to find strong evidence to support the potential therapeutic effect of TCM. It is crucial to carry out clinical practice of TCM and its effective ingredients and to discover the possibility of treating stroke with integrated Chinese and western medicine. Multi-mode treatment of stroke can be explored to improve the possible direction of stroke treatment (Yang et al., 2016; Liu et al., 2018; Duan T. et al., 2022; Zhu et al., 2022). In conclusion, we propose embracing a network disease perspective when considering stroke, as it highlights the significance of acknowledging the multifaceted nature of stroke, including its multiple targets, pathways, and channels. By adopting this approach, we can facilitate the development of novel therapeutic strategies to effectively treat stroke.

## Author contributions

BC drafted the manuscript. WJ provided the analysis and helped in the interpretation of results. Both authors approved the final version of the manuscript.
