# Supplementary material for: A comprehensive review of stroke-related signaling pathways and treatment in western medicine and traditional Chinese medicine
Source: Front Neurosci. 2023 Jun 7;17:1200061. doi: 10.3389/fnins.2023.1200061 (PMC10282194; doi:10.3389/fnins.2023.1200061)
Supplement: Supplementary file 1 [file Data_Sheet_1.docx]

# Appendix

We put the tables involved in the main text in the appendix for display, and the following is the description of each table.

Table 2: This table provides an overview of the pathophysiological mechanisms involved in stroke, including angiogenesis, oxidative stress, autophagy, inflammation, and apoptosis. It outlines the historical process of these mechanisms and their relationship with stroke, providing a basis for the classification of stroke-related signaling pathways.

Table 3: This table classifies the signaling pathways related to stroke based on the pathophysiological mechanisms involved. It provides information on the origin, historical process, and association with stroke of each signaling pathway. This information helps explore the initial role of these signaling pathways and provide innovative ideas for future research. Examples of angiogenesis-related stroke signaling pathways include Rho/Rock signaling pathway, Wnt/β-catenin signaling pathway, VEGF signaling pathway, and NO signaling pathway.

Table 4:This table summarizes the historical process, advantages, and disadvantages of recent key western medicine methods in the treatment of stroke. It is divided into surgical treatment (arterial thrombolysis, angioplasty, mechanical thrombectomy), and drug treatment (intravenous thrombolysis, antiplatelet, defibrase, neuroprotective drugs).

Table 5: We have summarized recent experimental studies on western medicine for stroke and organized them based on the classification of stroke-related signaling pathways. Our analysis reveals that only a few western medical treatments have the capability to act on more than one signaling pathway. The tables containing the detailed information on these studies have been moved to the appendix for display.

Table 6:This table shows the experimental methods of each Traditional Chinese Medicine (TCM) in the treatment of stroke, including experimental models and experimental results. The table is organized based on the classification of stroke-related signaling pathways, helping to identify which TCMs may be effective for specific pathophysiological mechanisms.

Table 2: An overview of pathophysiological mechanisms of stroke

| **Aspects** | **Association with stroke** |
| --- | --- |
| Angiogenesis | **1994** Increased angiogenesis after stroke([Krupinski et al., 1994](#_ENREF_189))  **1996-2001**Research has demonstrated that growth factors like TGF-β1 can stimulate the growth of new blood vessels in the brain, known as cerebral angiogenesis. Thus, using growth factors to induce angiogenesis in stroke patients holds great potential as a therapeutic approach.([Krupinski et al., 1997](#_ENREF_188), [Croll and Wiegand, 2001](#_ENREF_70)) |
| Oxidative stress | 1**993** Neurodegenerative diseases, including stroke, are associated with oxidative stress as a key underlying mechanism. When the body produces an excess of reactive oxygen species (ROS), it can lead to oxidative free radical damage, causing harm to cells and tissues. Ischemic stroke is particularly susceptible to this type of damage.([Coyle and Puttfarcken, 1993](#_ENREF_69)) |
| Autophagy | **1995** It was discovered, for the first time, that autophagy is activated following cerebral ischemia. This process, which involves the degradation and recycling of cellular components, was found to be initiated in response to the brain's reduced blood supply during ischemia.([Nitatori et al., 1995](#_ENREF_249)) |
| Inflammatory | **1996** Following injuries such as trauma, ischemia, or infection, the brain is capable of producing a distinct inflammatory response. This reaction entails a complicated series of events, which includes the discharge of inflammatory molecules and the triggering of immune cells.([Arvin et al., 1996](#_ENREF_16)) |
| Apoptosis | **1995**Following either systemic or focal ischemic injury, a large number of brain neurons undergo apoptosis. This process, which involves programmed cell death, occurs as a result of the brain's response to the ischemic insult.([Bredesen, 1995](#_ENREF_30)) |

Table 3: Overview of research on stroke-related signaling pathways

| **Aspects** | **Pathways** | **Origins** | **Development** | **Association with stroke** |
| --- | --- | --- | --- | --- |
| Angioge-nesis | Rho/Rock signaling pathway | **1985**The Rho gene was first discovered and named by Pascal Madaule and colleagues in their research.([Madaule and Axel, 1985](#_ENREF_228))  **1987**Aktories, K. et al. found that extracellular C3 enzymes.([Ohashi and Narumiya, 1987](#_ENREF_259), [Aktories et al., 1987](#_ENREF_4))  **1988**In their research, Morii and colleagues showed that the Rho gene encodes a protein that binds to extracellular C3 enzymes. This finding established a connection between the Rho gene and these enzymes.([Narumiya et al., 1988](#_ENREF_247), [Morii et al., 1988](#_ENREF_242)) | **1992-1995**Narumiya and colleagues identified the cellular functions of Rho as a molecular switch for actin, which plays a role in influencing cell motility.([Ridley and Hall, 1992](#_ENREF_271), [Kishi et al., 1993](#_ENREF_180))  **1996-1999**Y-27632, an inhibitor of Rho/Rock, was discovered in 1997, and fasudil, marketed in Japan, exhibited effects on angiogenesis and arterial vasospasm in subarachnoid hemorrhage.([Uehata et al., 1997](#_ENREF_323), [Shibuya et al., 2005](#_ENREF_290))  **2000-2006**The Rho/Rock signaling pathway holds promise as a therapeutic target for cardiovascular diseases and is believed to play a role in VEGF-induced angiogenesis, possibly by phosphorylating Ezrin and inducing transcriptional repression.([van Nieuw Amerongen et al., 2003](#_ENREF_327), [Hirooka and Shimokawa, 2005](#_ENREF_134), [Kishore et al., 2005](#_ENREF_181))  **2007-Now**The Rho/Rock signaling pathway has been implicated in various physiological and pathological processes, including neural regeneration, tissue repair, inflammation, immune response, apoptosis, and astrocyte function.([Lu et al., 2021](#_ENREF_225), [Zanin-Zhorov et al., 2014](#_ENREF_387)) | **2001-2007**Studies have demonstrated that Rho and Rho kinase expression is elevated following stroke and that the Rho/Rock pathway exerts a regulatory effect on endothelial cells, thereby facilitating the upregulation of eNOS activity.([Shin et al., 2007](#_ENREF_294), [Yagita et al., 2007](#_ENREF_368))  **2008-NOW**The Rho/Rock([Zanin-Zhorov et al., 2014](#_ENREF_387)) signaling pathway is known to play an angiogenic role in the subarachnoid hemorrhage subtype.([Tachibana et al., 1999](#_ENREF_308))Subsequent studies have suggested a correlation between the Rho/Rock pathway and angiogenesis and neuroprotection following stroke, although further experimental evidence is needed to fully elucidate its role.([He et al., 2013](#_ENREF_128)) |
|  | Wnt/β-catenin signaling pathway | **1982**Int-1 was isolated from mouse mammary tumor virus by Roe1 Nusse et al.([Nusse and Varmus, 1982](#_ENREF_254))  **1987-1991**Int1 gene plays an important role in the normal embryonic development of mice, and can make the normal embryonic development of Drosophila appear Wingless. Roe1 Nusse combined wingless with int1 and named Int1 as Wnt gene.([Nusse et al., 1991](#_ENREF_253)) | The Wnt signaling pathway was pioneered by Roe1 Nusse, and then the Wnt/β-catenin signaling pathway has a new development. Detailed target information of this pathway can be found on the Stanford website.  **1990-1992**The Wnt gene family signaling was recognized as a gene signal involved in embryonic development and tumor development.([Noordermeer et al., 1994](#_ENREF_251))  **1996** The seven-transmembrane receptor Frizzled (FZD) was identified as a cell surface receptor for Wnt signaling ligands in the Wnt signaling pathway. Additionally, β-catenin was shown to interact with the LEF-TCF complex and function in the nucleus.([Bhanot et al., 1996](#_ENREF_25), [Siegfried et al., 1994](#_ENREF_296), [Huber et al., 1996](#_ENREF_152))  **1999-2004**Various protein regulators at different points in the Wnt pathway were discovered, leading to gradual perfection of the pathway. During this time, studies found overexpression of Wnt, or FZD with SFRP silencing during carcinogenesis, linking the Wnt pathway to cancer.([Ugolini et al., 2001](#_ENREF_324))  **2005-2022** Research has focused on studying the role of the Wnt signaling pathway in tissue formation, nerve repair, and tumor development.([Nusse, 2005](#_ENREF_252), [Klaus and Birchmeier, 2008](#_ENREF_183)) | **2001**GSK-3β plays a crucial role in the Wnt signaling pathway, and its potential involvement in stroke has been suggested.([Frame and Cohen, 2001](#_ENREF_100))  **2001-2010**There is a possibility that the Wnt/β-catenin signaling pathway participates in the mechanisms of post-stroke neurogenesis and angiogenesis.([Zerlin et al., 2008](#_ENREF_389), [Shruster et al., 2012](#_ENREF_295))  **2016-2022**Dogwood, acetylshikonin, electroacupuncture, XQ-1H, and oligodendrocyte precursor cells are potential mediators of angiogenesis and neurogenesis in stroke through the Wnt/β-catenin signaling pathway.([Xu et al., 2016](#_ENREF_366), [Li et al., 2018b](#_ENREF_216), [Shi et al., 2022](#_ENREF_289), [Wang et al., 2022c](#_ENREF_340), [Xu et al., 2019](#_ENREF_363)) |
|  | VEGF signaling pathway | **1983**D.R. Senger et al. identified a novel permeability factor called VPF from tumors.([Senger et al., 1983](#_ENREF_288))  **1989**N Ferrara and D W Leung et al. isolated and uniformly named VEGF in bovine pituitary follicular cells.([Ferrara and Henzel, 1989](#_ENREF_97), [Leung et al., 1989](#_ENREF_201)) | **1990-2000**Researchers have discovered the VEGF receptor tyrosine kinase.([Veikkola and Alitalo, 1999](#_ENREF_329))  **2000-2005**VEGF serves as a mitogen for endothelial cells and induces angiogenesis, regulating growth and development. It plays a crucial role in tumor or angiogenesis and also mediates endothelial cell proliferation.([McMahon, 2000](#_ENREF_236), [Ferrara, 2001](#_ENREF_96))  **2005-2010** Researchers have directed VEGF into clinical treatment, and have conducted clinical trials and studies in the treatment of cancer.([Waldner and Neurath, 2012](#_ENREF_333))  **2010-2022**The mechanism of VEGF is well understood, and its effects have gradually expanded to include nerve, cardiovascular, cerebrovascular, lymphatic vessels, and ocular functions. It has been found to have various effects, such as promoting nerve migration, inducing angiogenesis, and providing neuroprotection.([Shim and Madsen, 2018](#_ENREF_292), [Karaman et al., 2018](#_ENREF_173)) | **1999-2003**VEGF has been linked to ischemic stroke and is known to promote angiogenesis, neurogenesis, and neuroprotection following such strokes.([van Bruggen et al., 1999](#_ENREF_326), [Zhang et al., 2000](#_ENREF_399))  **2004-2015**The use of VEGF therapy and VEGF-mediated therapy has been investigated.^(^[^Ishikawa et al., 2013^](#_ENREF_157)^)^  **2016-2022**The early or acute upregulation of VEGF or its treatment may lead to increased vascular permeability, thereby exacerbating cerebral edema.([Hu et al., 2022b](#_ENREF_146)) |
|  | NO signaling pathway | **1978-1984**R. Furchgott discovered that nitric oxide could act as a messenger molecule regulating blood vessels, and that the endothelium produces a humoral factor known as vascular relaxing factor (EDRF).([Furchgott and Zawadzki, 1980](#_ENREF_105), [Griffith et al., 1984](#_ENREF_117))  **1987** IGNARRO L J et al. discovered that NO and EDRF are in fact the same substance.([Ignarro et al., 1987](#_ENREF_153), [Palmer et al., 1987](#_ENREF_262)) | **1991-2000**As our understanding of the NO signaling pathway and its pathophysiological mechanisms continues to advance, it has become increasingly clear that maintaining appropriate levels of NO can have a significant positive impact on vascular regulation and host defense.([Gross and Wolin, 1995](#_ENREF_118))  **2000-2010**As drugs that target the NO signaling pathway have advanced, the clinical application of NO has become increasingly refined.([Zhang et al., 2011](#_ENREF_395))  **2011-2022**NO has been established as a key regulator of several bodily systems, including the reproductive system, immune system, circulatory system and central nervous system. The clinical significance and potential for further development of NO-based therapies are therefore significant.([Cinelli et al., 2020](#_ENREF_61))  **2017** At the same time, the links between hydrogen sulfide, PI3K/AKT signaling pathway, HIF and NO were found.([Szabo, 2017](#_ENREF_307)) | **1993-1995**The treatment of cerebral ischemia has been observed to enhance the function of nitric oxide synthase, which results in the generation of nitric oxide (NO) and consequent activation of guanylate cyclase.([Kader et al., 1993](#_ENREF_170))  **1997-2005**Inhibiting Neuronal nitric oxide synthase (nNOS) can lead to negative consequences such as increased neurotoxicity, altered neurogenesis, and increased infarct size following a stroke.([Willmot and Bath, 2003](#_ENREF_355), [Endres et al., 2004](#_ENREF_91))  **2006-2022** NO plays a crucial role in regulating cerebral blood flow and can also produce peroxynitrite, which has been implicated in mediating cerebral ischemia/reperfusion (I/R) injury. This injury can cause the destruction of the blood-brain barrier and nerve cell death.([Wang et al., 2022d](#_ENREF_347)) |
| Oxidative stress | SHH signaling pathway | **1980**Wieschaus, E. et al. were responsible for identifying the hedgehog gene (hh) in Drosophila melanogaster.([Nüsslein-Volhard and Wieschaus, 1980](#_ENREF_255))  **1993**Three homologs of hh were discovered, and it was determined that SHH is specifically associated with the central nervous system.([Echelard et al., 1993](#_ENREF_90))  **1997-1999** Targets of the SHH signaling pathway, known as Gil 1-3, were discovered, and it was found that Gli 2-3 are the active forms that activate downstream factors, including Gli 1.([Sasaki et al., 1999](#_ENREF_280)) | The SHH signaling pathway is comprised of several components, including the signaling molecule Hedgehog, two transmembrane receptors (Ptch and Smo), and nuclear transcription factors.  **1998-2009**The SHH signaling pathway is a critical pathway in both developmental biology and cancer, and its components, such as Gli, have been found to be involved in tumorigenesis and have received significant attention in research.([Matise and Joyner, 1999](#_ENREF_232))The role of the SHH signaling pathway in vascular biology was initially discovered during embryonic development.([Pepicelli et al., 1998](#_ENREF_265))  **2009-2014**The SHH signaling pathway can be impacted by the inhibition of Gli signaling. SMO antagonists, Vismodegib, have been developed and marketed, and the SHH signaling pathway is now being utilized for cancer treatment and has entered the stage of clinical application.([Infante et al., 2015](#_ENREF_154))  **2015-NOW** Inhibitors of SHH signaling pathway focus on inhibiting SMO, but few clinical treatment methods are approved. At the same time, it is necessary to pay attention to drug resistance and precise treatment of specific diseases.([Wu et al., 2017a](#_ENREF_356)) | **1998-2017**The SHH signaling pathway is associated with stroke and has been found to play a protective role by promoting anti-oxidation, anti-apoptosis, neurogenesis, and angiogenesis, ultimately leading to stroke protection.([Huang et al., 2013](#_ENREF_149))  **2018-NOW**Resveratrol, an important component, has been found to protect against stroke by improving stroke and neurological function through the SHH signaling pathway.([Yu et al., 2021](#_ENREF_380), [Yu et al., 2017](#_ENREF_381))Although the relationship between the SHH signaling pathway and stroke has been established, further research is needed to fully understand it. Additionally, more investigation is required to develop therapeutic drugs and methods based on this pathway, and researchers are actively pursuing this avenue of study. |
|  | Nrf2/ARE signaling pathway | **1994**P. Moi et al. isolated and identified the Nrf2 gene.([Moi et al., 1994](#_ENREF_241))  **1996-1998**Mutations in Nrf2 cause anemia and cell death in mice.([Chan et al., 1998](#_ENREF_40))  **1999**The upstream factor Keap1 has been identified as a mediator of oxidative stress through antioxidant response elements with Nrf2.([Itoh et al., 1999](#_ENREF_160))  **1999**Nrf2 is responsible for inducing the expression of heme oxygenase-1.([Alam et al., 1999](#_ENREF_5)) | **2000-2004**The Nrf2 signaling pathway has been found to cross-talk with other signaling pathways, such as the MAPK signaling pathway, PKC kinase, PI3K signaling pathway, among others.([Alfieri et al., 2011](#_ENREF_7))  **2004-2008**The Nrf2/ARE signaling pathway is involved in the onset and progression of various diseases, such as cancer, neurodegenerative disorders, cardiovascular diseases, autoimmune diseases, and inflammation.([Hayes et al., 2010](#_ENREF_126), [Zhang, 2006](#_ENREF_392))The Nrf2/ARE signaling pathway has also been found to have a "dark side" in cancer.([Lau et al., 2008](#_ENREF_197))  **2008-2012** In the development of clinical trials, dimethyl fumarate was found to be effective in treating multiple sclerosis during Phase III clinical trials, and it was subsequently marketed in 2013.([Gold et al., 2012](#_ENREF_109))  **2016**Nrf2 plays a role in regulating mRNA translation in pancreatic cancer and has been found to be associated with both lung and pancreatic cancer.([Chio et al., 2016](#_ENREF_59))  **NOW** The Nrf2/ARE signaling pathway has been found to be associated with the inflammatory response, and the dual role of this pathway in disease should be taken into account.([Ahmed et al., 2017](#_ENREF_3)) | **2004-2007**Astrocytes expressing Nrf2 have been shown to provide protection to neurons from oxidative stress.([Kraft et al., 2004](#_ENREF_186))  Activation of Nrf2 in vivo has been shown to have a protective effect against ischemic stroke by reducing infarct size and penumbra.([Shih et al., 2005](#_ENREF_291))  **2008-2018**In preclinical studies using cell and animal models, it has been demonstrated that Nrf2 and its downstream factor HO-1 are activated in response to stroke, and pharmacological interventions targeting this pathway can protect against stroke through the Nrf2/HO-1 defense mechanism.([Alfieri et al., 2013](#_ENREF_8), [Alfieri et al., 2011](#_ENREF_7))  **2018-2020**The Phase II clinical trial aimed to investigate the effects of dimethyl fumarate on infarct size and NIHSS score in patients with acute ischemic stroke, compared to placebo. The trial is registered at en.irct.ir/trial/34326. |
|  | HIF-1α signaling pathway | **1991** Gregg L. Semenza discovered the HIF (hypoxia-inducible factor) protein complex which binds to a specific DNA sequence and responds to changes in oxygen levels.([Semenza et al., 1991](#_ENREF_286))  **1998**HIF-1α deletion can severely hinder vascular development and the expression of oxygen-dependent genes has been demonstrated.([Carmeliet et al., 1998](#_ENREF_38)) | **2001**HIF-1 was shown to be hydroxylated by proline hydroxylase and further lead to proteasome degradation.([Ivan et al., 2001](#_ENREF_161), [Jaakkola et al., 2001](#_ENREF_162))  **2016**The research on hypoxia-inducing factors won the Lasker Prize.([Thompson, 2016](#_ENREF_318))  **2019**The Nobel Prize in Physiology or Medicine, the most prestigious honor in the scientific community, was bestowed upon William G. Kaelin Jr. from the United States, Peter J. Ratcliffe from the United Kingdom, and Gregg L. Semenza from the United States.(https://www.nobelprize.org/) | **2003-2005**HIF-1α and its target genes are known to play a role in regulating brain injury after stroke.([Helton et al., 2005](#_ENREF_129))  **2011** Studies suggest that HIF-1α plays a biphasic role in both in vivo and in vitro ischemic stroke models.([Yeh et al., 2011](#_ENREF_375))  **2014-2020** The HIF-1α signaling pathway has been linked to various processes involved in stroke, including inflammatory response, angiogenesis, and neuroprotection.([He et al., 2021](#_ENREF_127), [Cheng et al., 2014](#_ENREF_56)) |
|  | PPAR-γ signaling pathway | **1990**Issemann et al. first discovered that a class of fatty acid-like compounds called peroxisome proliferators (PP) can activate a type of enzyme, which was named PPAR (PP-activating receptor).([Issemann and Green, 1990](#_ENREF_158)) | **2007**Discovery of PPAR agonist, type 2 diabetes drug enters clinical trials.([Culman et al., 2007](#_ENREF_73))  **2010-2020** PPARs play a crucial role in regulating lipid, glucose, and amino acid metabolism, as well as mediating inflammatory responses, apoptosis, and differentiation. They can be regulated by non-genetic crosstalk with various signaling pathways, including phosphatases and kinases. Dysregulation of PPAR signaling has been implicated in several diseases, including cancer.([Zolezzi et al., 2017](#_ENREF_412)) | **2006-2010**PPAR-α agonists have the potential to decrease oxidative stress, cell apoptosis, and inflammatory response, making them a promising target for stroke treatment.([Fong et al., 2010](#_ENREF_98), [Culman et al., 2007](#_ENREF_73))  **2014-2020**PPAR-α agonists have demonstrated efficacy against stroke.([Liu and Wang, 2017](#_ENREF_221)) |
| Autopha-gy | Pink1/Parkin signaling pathway | **1998**The protein mutated in Parkinson's disease is named Parkin.([Kitada et al., 1998](#_ENREF_182))  **2004**Mutations in Pink1 have been linked to Parkinson's disease.([Valente et al., 2004](#_ENREF_325)) | **2006** Pink1/Parkin has been shown to co-regulate mitochondrial autophagy in Drosophila.([Clark et al., 2006](#_ENREF_62))  **2010**The protein Pink1 is a crucial sensor of mitochondrial damage.([Matsuda et al., 2010](#_ENREF_233))  **2010-2014**Pink1 recruits and phosphorylates Parkin, which in turn mediates mitochondrial autophagy.([Geisler et al., 2010](#_ENREF_107), [Fedorowicz et al., 2014](#_ENREF_94))  **2014-2017**Pink1 is also involved in mediating mitochondrial autophagy that is independent of Parkin..([Dave et al., 2014](#_ENREF_76))  **2022**Pink1 and Parkin are two key proteins associated with the pathogenesis of Parkinson's disease, and their encoding genes have become the main hereditary risk factors for neurodegenerative diseases.([Klein and Westenberger, 2012](#_ENREF_184), [Zhong et al., 2022](#_ENREF_404)) | **2002** Transient focal cerebral ischemia leads to a significant decrease in Parkin protein levels, increased neuronal vulnerability to endoplasmic reticulum dysfunction, and accumulation of ubiquitinated proteins, which ultimately results in cell damage.([Mengesdorf et al., 2002](#_ENREF_237))  **2013-2016**Increasing the levels of Parkin and Pink1 has been shown to decrease the protective effect of Drp1 against cerebral ischemia.([Zhao et al., 2013](#_ENREF_402), [Tang et al., 2016](#_ENREF_313))  **2018-2022** Hydrogen, electroacupuncture, thyroid hormone, and docosahexaenoic acid have been shown to reduce mitochondrial dysfunction by modulating the Pink1/Parkin signaling pathway, promoting mitochondrial autophagy function, and protecting against neuronal apoptosis in stroke.([Wu et al., 2018](#_ENREF_359), [Wang et al., 2019](#_ENREF_336), [Chang et al., 2022](#_ENREF_42), [Sun et al., 2022](#_ENREF_303)) |
|  | Bnip3 signaling pathway | **1994**Bnip3 (formerly NIX3), three cDNAs of unknown function initially isolated from adenoviruses.([Boyd et al., 1994](#_ENREF_28))  **1997**Bnip3 (NIX3) is associated with apoptosis.([Chen et al., 1997](#_ENREF_45))  **1998**BNIP3L was isolated, identified, and found to be associated with inhibiting tumor growth.([Matsushima et al., 1998](#_ENREF_234)) | **2006-2010**BNIP3 is a pro-apoptotic factor that is upregulated under hypoxic conditions, and it has been shown to play a role in both the cardiovascular system and cancer.([Chinnadurai et al., 2008](#_ENREF_58), [Burton and Gibson, 2009](#_ENREF_33))  **2010-2020**BNIP3/BNIP3L, downstream targets of hypoxia-inducible factor (HIF), have the ability to induce both mitochondrial autophagy and apoptosis.([Zhang et al., 2019](#_ENREF_396)) | **2007-2011** Bnip3 is involved in hypoxia and the process of delayed neuronal death after stroke.([Zhang et al., 2007](#_ENREF_398))  **2017**BNIP3/NIX induces autophagy in response to stroke.([Yuan et al., 2017](#_ENREF_384))  **2017-2020** The HIF-1α cascade and Bnip3 play a role in apoptosis and autophagy after stroke.([Zhang et al., 2019](#_ENREF_396)) |
|  | P53 signaling pathway | **1979**Lane and Crawford identified a protein that interacts with SV40 large T antigen in cells of SV40-infected mice and named it p53 (the corresponding gene in humans is called TP53).([DeLeo et al., 1979](#_ENREF_78))  **1991**P53 has the ability to trigger apoptosis.([Yonish-Rouach et al., 1991](#_ENREF_377)) | In the field of P53 research, over 50,000 papers have been published, making it an essential topic for researchers studying cancer signaling pathways.([Levine, 2020](#_ENREF_203))  In the first decade of research, the P53 gene was found to be one of the most commonly mutated genes in humans, with the p53 protein being identified as a tumor suppressor.([Harris, 1993](#_ENREF_122), [Hollstein et al., 1991](#_ENREF_137), [Levine et al., 1991](#_ENREF_204))  During the second decade of research, it was discovered that p53 functions as a transcription factor that can be activated in response to cellular stress, leading to cell cycle arrest, apoptosis, or senescence.([Herr and Debatin, 2001](#_ENREF_131), [Vousden and Lu, 2002](#_ENREF_330), [Aylon and Oren, 2007](#_ENREF_19))  In the third decade, studies suggested that p53 may have an oncogenic role, and researchers began focusing on developing anticancer drugs that target the p53 protein.([Bartlett et al., 2014](#_ENREF_23))  In the fourth decade, P53 is a carcinogenic process involving multiple proteins, which presents challenges for the development of effective drug therapies and underscores the need for further research.([Muller and Vousden, 2014](#_ENREF_244), [Wang et al., 2023](#_ENREF_337)) | **1994**The expression of P53 protein increases following a focal ischemic stroke.([Li et al., 1994](#_ENREF_213))Peg3/Pw1, a downstream factor of P53, participates in the signaling pathway of p53-mediated cell death.([Yamaguchi et al., 2002](#_ENREF_369))  **2001-2010** Inhibitors of P53 can decrease the number of apoptotic cells in the ischemic brain and improve neurological function.([Leker et al., 2004](#_ENREF_199), [Luo et al., 2009](#_ENREF_226))  **2010-2020**Death-associated protein kinase 1 (DAPK1) could act as a protective mediator in cerebral ischemia, potentially mediated by P53.([Wang et al., 2017](#_ENREF_342), [Wang et al., 2014](#_ENREF_346)) |
| Inflamm-atory | NF-κB signaling pathway | **1986**David Baltimore and Sen were the pioneers who discovered a new transcription factor called NF-κB (nuclear factor kappa-light-chain-enhancer of activated B cells), which attaches to immunoglobulin enhancers in activated B cells.([Sen and Baltimore, 1986](#_ENREF_287)) | **1996-2004**The NF-κB signaling pathway is closely associated with inflammatory responses, immunity, and apoptosis,and its involvement has been observed in various diseases such as cancer, arthritis, atherosclerosis, and diabetes.([Brand et al., 1996](#_ENREF_29), [Pahl, 1999](#_ENREF_260), [Kumar et al., 2004](#_ENREF_191))The specific regulatory mechanisms of the NF-κB pathway have been elucidated, including the involvement of convertors TRAFs, key adapter RIPs, and the IKK complex within the pathway.([Hayden and Ghosh, 2008](#_ENREF_125))  **2004-2012**In 2004, groundbreaking research and substantial evidence revealed that NF-κB signaling can be activated by inflammatory cytokines in cancer.([DiDonato et al., 2012](#_ENREF_79), [Greten et al., 2004](#_ENREF_116), [Pikarsky et al., 2004](#_ENREF_267)) Following the approval of bortezomib (Velcade), which targets NF-κB, for treating multiple myeloma, there has been a growing interest in developing immunotherapies that inhibit NF-κB signaling for cancer treatment.([Kim et al., 2006](#_ENREF_179), [Couzin-Frankel, 2013](#_ENREF_68))  **2013-NOW**NF-κB is a critical signaling molecule involved in inflammation, immunity, and cancer development. Further investigation is necessary to explore how to translate the role of inflammation in tumors into clinical immunotherapies. | **1998-2005**The NF-κB signaling pathway is closely linked to the pathology of neurodegenerative diseases, and its activation following a stroke can lead to cell death and contribute to the formation of infarction.([Lezoualc'h and Behl, 1998](#_ENREF_205), [Schneider et al., 1999](#_ENREF_284))  **2006-2018**The mechanism underlying the NF-κB signaling pathway after a stroke has been gradually elucidated, involving the inflammatory response following a stroke, regulation of inflammasomes, anti-apoptotic proteins, and its activation having a time-dependent dual effect.([Yang et al., 2007](#_ENREF_371)) |
|  | TLRs signaling pathway | **1985**Anderson, K. V. et al. established the dorsoventral pattern of Drosophila embryos and discovered the Toll gene, which they subsequently named.([Anderson et al., 1985](#_ENREF_11))  **1988**Hashimoto, C. et al. characterized the structure of the Toll protein.([Hashimoto et al., 1988](#_ENREF_124))  **1994**Nomura et al. were the first to report on Toll-like receptors in humans.([Nomura et al., 1994](#_ENREF_250)) | Toll-like receptors (TLRs) are a type of pathogen pattern recognition receptor that can identify specific pathogen-associated molecular patterns (PAMPs). TLR signaling pathways include both MyD88 and TRIF-dependent pathways.  **1996**This indicates that Toll receptors and their signaling are significant in the field of immunology.([Lemaitre et al., 1996](#_ENREF_200))  **1997-2004**TLR signaling pathways induce inflammatory responses, coordinate dendritic cells, sense foreign invasion, and connect innate immunity to induce adaptive immunity, producing a series of host defense responses. TLRs are involved in sepsis, immune deficiency, atherosclerosis, asthma, and other diseases.([Cook et al., 2004](#_ENREF_65))  **2004-2010** Targeted manipulation of TLR signaling is used as an adjunct to therapy, and the NF-κB signaling pathway is partially linked to the TLR signaling pathway.([Kawai and Akira, 2007](#_ENREF_175))  **2010-2020**TLR signaling mediates malignant transformation, tumor progression, immune evasion processes, and plays a crucial role in the tumor microenvironment.([Ridnour et al., 2013](#_ENREF_272))  **2020** The TLR signaling pathway has also garnered significant attention in the field of tumor immunotherapy, and a connection has been discovered between immunity and metabolism.([Chou et al., 2022](#_ENREF_60)) | **2004** Research has shown that blocking Toll-like receptor and proinflammatory cytokine signaling can contribute to inducing ischemic tolerance in the brain.([Karikó et al., 2004](#_ENREF_174))  **2007-2012**Studies have shown that TLR2 or TLR4-deficient mice exhibit reduced brain damage and neurological deficits caused by ischemia-reperfusion injury, indicating the potential protective role of TLR inhibition in stroke.([Lehnardt et al., 2007](#_ENREF_198)) Additionally, TLR8 has been found to play a deleterious role in post-stroke inflammation and neuronal death.([Tang et al., 2013](#_ENREF_314))  **2020**The TLRs signaling pathway-mediated inflammatory response after stroke has both beneficial and detrimental effects, and its translation into clinical applications needs further investigation.([Nalamolu et al., 2021](#_ENREF_246)) |
|  | IL-33/ST2 signaling pathway | **2005**In 2005, Baekkevold and colleagues made a breakthrough discovery in identifying IL-33, a cytokine with multifunctional properties.([Schmitz et al., 2005](#_ENREF_283))  **2007-2008**The signaling pathway for IL-33, a cytokine, involves the activation of the ST2 receptor.([Carriere et al., 2007](#_ENREF_39)) | **2008-2010**IL-33 plays a crucial role in various diseases, including inflammation and infection. It is known to function not only as a transcription factor within the nucleus, but also to activate mast cells, lymphocytes, and eosinophils via its membrane receptor ST2, leading to the production of Th2 cytokines.([Palmer and Gabay, 2011](#_ENREF_261))  **2012-2020**The IL-33/ST2 pathway plays a critical role in the initiation, progression, and prognosis of various types of tumors.([Jovanovic et al., 2012](#_ENREF_167)) | **2015-2018** The IL-33/ST2 axis is known to promote Th2-type immune response, thus playing a key role in the immune regulatory mechanism following ischemic stroke, and ultimately reducing the cerebral infarction area and providing neuroprotection. Celastrol has been shown to decrease the expression of inflammatory cytokines following ischemic stroke via the IL-33/ST2 axis.([Jiang et al., 2018a](#_ENREF_164)) |
| Apoptosis | Notch signaling pathway | **1910-1920**The NOTCH gene was initially discovered and characterized through studies of the notch-winged mutant of Drosophila melanogaster, which served as the inspiration for its naming and subsequent research.([Bridges, 1916](#_ENREF_31), [Metz and Bridges, 1917](#_ENREF_238), [Yochem et al., 1988](#_ENREF_376))  **1980s**The Notch gene was successfully isolated and sequenced in Drosophila melanogaster, and subsequent research revealed that the Notch protein is capable of transmembrane signaling.([Artavanis-Tsakonas et al., 1983](#_ENREF_14), [Wharton et al., 1985](#_ENREF_353), [Kidd et al., 1986](#_ENREF_178)) | **1988-1990** LIN-12, GLP-1, and XOTCH were identified as homologs of the Notch gene, while cdnas in mammals were isolated and cloned.([Coffman et al., 1990](#_ENREF_64), [Austin and Kimble, 1989](#_ENREF_18), [Yochem et al., 1988](#_ENREF_376))  **1991-1997**The Notch gene was initially linked to acute T cell lymphoblastic leukemia, and coding mutations in Notch have also been identified in Alagille syndrome (AGS). As such, the Notch gene is considered to be an important gene associated with human health.([Oda et al., 1997](#_ENREF_258))  **2000**The Notch signaling pathway plays a critical function in several biological systems, including the heart and brain, immune hematopoiesis, and hepatobiliary metabolic system. In recent times, it has received considerable attention in cancer research as well.([Andersson and Lendahl, 2014](#_ENREF_12))  **2010-2012** The central regulatory mechanisms of Notch signaling are described in detail.([Andersson et al., 2011](#_ENREF_13))  **2019-NOW**There are also potential crosstalk between Notch signaling pathway and other Wnt signaling pathways, TGF-β/BMP signaling pathway, GSK-3β signaling pathway, Ras/MAPK signaling pathway and autophagy signaling pathway.([Sarin and Marcel, 2017](#_ENREF_279), [Hansson et al., 2004](#_ENREF_121), [Andersson et al., 2011](#_ENREF_13)) | **2000-2004**Notch signaling pathway is increasingly related to organogenesis and angiogenesis. Notch gene mutations are increasingly understood as one of the causes of stroke.([Campos et al., 2002](#_ENREF_34), [Ito et al., 2002](#_ENREF_159))  **2006** The esteemed journal Nat Med highlighted in 2006 that the Notch signaling pathway could serve as a therapeutic target for stroke and other neurodegenerative diseases.([Arumugam et al., 2006](#_ENREF_15))  **2006-2010**Although Notch signaling can have therapeutic benefits for stroke, in certain situations it may also be detrimental after a stroke.([Wei et al., 2011](#_ENREF_350), [Albéri et al., 2010](#_ENREF_6))  **2011-2022** The Notch signaling pathway plays a crucial role in promoting angiogenesis and protecting the blood-brain barrier after stroke when Danhong injection is combined with tPA. Furthermore, lipoxin A4 can regulate the polarization of microglia after stroke.([Chen et al., 2022d](#_ENREF_52), [Li et al., 2021c](#_ENREF_212)) |
|  | Hippo signaling pathway | **1995**The first crucial genes of the Hippo pathway in Drosophila, Wts (Warts or Lats), were identified through genetic screening.([Justice et al., 1995](#_ENREF_168), [Xu et al., 1995](#_ENREF_365))  **2002**The laboratories of Hariharan and Halder identified a second gene in the Hippo pathway, Sav, which is connected to the Wts gene and promotes apoptosis.([Kango-Singh et al., 2002](#_ENREF_172), [Tapon et al., 2002](#_ENREF_316)) | The Hippo signaling pathway, also recognized as the Salvador/Warts/Hippo (SWH) pathway, has emerged as a prevalent research focus in recent times. It was initially detected in tissues of Drosophila melanogaster.**2003-2005**The tumor suppressor Mat was discovered to interact with the Wts gene, enhancing Wts kinase activity.([Lai et al., 2005](#_ENREF_194)) The Hpo gene program links the Wts gene with the Sav gene to control growth.([Wu et al., 2003](#_ENREF_358), [Harvey et al., 2003](#_ENREF_123), [Udan et al., 2003](#_ENREF_322)) Hpo can phosphorylate Sav to facilitate its interaction with Wts.([Pantalacci et al., 2003](#_ENREF_264)) In downstream transduction of the Wts gene, the Yap gene, identified through yeast hybridization, serves as a compensatory link.([Huang et al., 2005](#_ENREF_147))  **2004-2010**The Hippo signaling pathway is responsible for regulating the balance between apoptosis and proliferation during embryonic development.([Harvey et al., 2003](#_ENREF_123), [Varelas et al., 2008](#_ENREF_328), [Lian et al., 2010](#_ENREF_217))  **2011-2020**With more than 30 components, the Hippo signaling pathway has been studied from Drosophila to mammals. It impacts tissue regeneration, regulates organ volume, and plays a crucial role in cancer and cardiovascular diseases.([Moya and Halder, 2019](#_ENREF_243), [Zanconato et al., 2016](#_ENREF_386), [Tao et al., 2016](#_ENREF_315)) | **2016-2018**Hippo/Mst1 has been found to induce cell death via oxidative stress following cerebral ischemia.([Zhao et al., 2016](#_ENREF_400)) It also plays a role in neuronal apoptosis and microglia activation.([Li et al., 2018a](#_ENREF_207))  **2018-2022**Following cerebral ischemia/reperfusion (I/R) injury, Hippo/Yap is activated, and Yap has been shown to repair the blood-brain barrier to protect the injured brain.([Gong et al., 2021](#_ENREF_110)) The nuclear localization of Yap can reduce the release of inflammatory factors, thereby lessening brain injury caused by I/R.([Huang et al., 2020](#_ENREF_148)) |
|  | ErbB signaling pathway | **1970**The discovery of the EGF-binding protein (EGFR), which can stimulate organ growth in laboratory animals, led to the origins of research in this area.([Taylor et al., 1970](#_ENREF_317))  **1999**Scientists discovered that EGF activates the receptor by binding to EGFR, which triggers cell growth and division.([Wells, 1999](#_ENREF_352)) | **2000**The ErbB/HER signaling pathway receptors can activate membrane-bound protein kinases, promoting cell proliferation, differentiation, migration, and survival.([Wells, 1999](#_ENREF_352))  **2014** The ErbB/HER signaling pathway plays a crucial role in several diseases, including cancer, cardiovascular disease, neurological disorders, and immune-related conditions.([Tomas et al., 2014](#_ENREF_319))  **NOW** Several drugs that inhibit EGFR activity have been developed to treat lung, gastric, and other cancers. Additionally, scientists are exploring other therapeutic methods for the ErbB signaling pathway, including inhibiting the activity of other key molecules within the pathway, to improve patient outcomes. | **2000-2001**ErbB plays a critical role in promoting the survival of microglia and neurons in the nervous system, which is especially important in the context of brain injury.([Erlich et al., 2000](#_ENREF_92))  **2005-2010**The ErbB signaling pathway plays a crucial role in the repair and regeneration process after a stroke.([Xu and Ford, 2005](#_ENREF_367))  **2020**Scientists are developing agents that target EGFR to help restore function after a stroke. |
|  | TGF-β1/Smad3 signaling pathway | **1984**TGF-β1 was found and isolated.([Tam et al., 1984](#_ENREF_309))  **1986-1988**TGF-β1 has been found to have a dual effect, promoting both cell apoptosis and cell proliferation.([Roberts et al., 1985](#_ENREF_275))  **1996**The discovery of Smad3 was a significant breakthrough in the understanding of TGF-β signaling.([Lagna et al., 1996](#_ENREF_192), [Liu et al., 1996](#_ENREF_219)) | **1997**TGF-β1 operates via the Smad3 receptor, which is one of the two vital molecules in the TGF-β signaling pathway. This pathway can control several cellular functions such as cell proliferation, differentiation, migration, and apoptosis.([Attisano and Wrana, 2002](#_ENREF_17))  **2000-2010**The TGF-β1/Smad3 signaling pathway performs a pivotal function in numerous physiological processes such as inflammation, tissue repair, and cancer, along with the pathogenesis of several illnesses, including diabetes, cardiovascular diseases, and neurological disorders.([Kang et al., 2009](#_ENREF_171), [Gough et al., 2021](#_ENREF_111)) | **1996**After a stroke, the mRNA expression of TGF-β1 is increased.([Krupinski et al., 1996](#_ENREF_190))  **2001-2010**The anomalous functioning of the TGF-β/Smad signaling pathway may be linked to the onset of stroke.([Ali et al., 2001](#_ENREF_9))  **2018**Smad3 and TGF-β1 are involved in a protective mechanism against ischemic stroke.([Yu et al., 2018](#_ENREF_382)) |
| New cell death | Ferroptosis | Before the term ferroptosis was coined, there were descriptions of unique forms of cell death. These included cell death resulting from cystine deprivation due to reduced glutathione, non-apoptotic ROS-dependent cell death induced by ceramide, and the involvement of polyunsaturated fatty acids in cell death resulting from GPX4 knock-out.([Hirschhorn and Stockwell, 2019](#_ENREF_135))  **2012** Dixon et al. first described ferroptosis, a unique type of cell death that differs from apoptosis and necrosis, as a cell death pathway triggered by RSL3.([Dixon et al., 2012](#_ENREF_83)) | **2013** The induction of ferroptosis has been shown to enhance the anti-tumor activity of sorafenib in hepatocellular carcinoma (HCC).([Louandre et al., 2013](#_ENREF_224)) Furthermore, channelized influx of Ca^2+^ has been found to increase iron-induced ferroptosis in hippocampal HT22 cells.([Henke et al., 2013](#_ENREF_130)) Additionally, iron chelates have been found to inhibit iron-induced ferroptosis in primary neurons by targeting HIF prolyl hydroxylase.([Speer et al., 2013](#_ENREF_300))  **2014-2019** Researchers have found the key targets of ferroptosis, such as GPX4 and ASCL4.([Yang et al., 2014](#_ENREF_373), [Doll et al., 2017](#_ENREF_84))  **2020-NOW** Ferroptosis has been linked to various diseases, including tumors, nervous system disorders, blood disorders, and immune inflammation.([Stockwell et al., 2017](#_ENREF_302), [Chen et al., 2021b](#_ENREF_54), [Chen et al., 2021a](#_ENREF_53), [Li et al., 2021b](#_ENREF_211)) Several compounds have been discovered that induce ferroptosis, including experimental reagents like erastin and RSL3, approved drugs such as sorafenib, sulfasalazine, and statins, as well as cytokines like IFNγ and TGFβ1.([Chen et al., 2021a](#_ENREF_53)) Despite these findings, the molecular mechanisms underlying ferroptosis, its molecular recognition, and its potential advantages and disadvantages are still not entirely clear, and further research is needed to translate the knowledge of ferroptosis into clinical treatments.([Hirschhorn and Stockwell, 2019](#_ENREF_135)) | **2017** There is a correlation between ferroptosis and neurodegenerative diseases, and studies have shown that markers of ferroptosis increase after stroke both in vitro and in vivo.([Stockwell et al., 2017](#_ENREF_302), [Zille et al., 2017](#_ENREF_411))  **2019** Ferroptosis is linked to stroke, and after a stroke, there is an increase in GPX4 levels, iron accumulation, as well as depletion of oxygen and nutrients that contribute to the occurrence of ferroptosis.([Weiland et al., 2019](#_ENREF_351))  **2021-2022** Both knockout and overexpression of ASCL4 have been shown to impact the extent of injury in ischemic stroke.([Cui et al., 2021](#_ENREF_72), [Tuo et al., 2022](#_ENREF_321)) |
|  | Cuprotosis | **2022**The buildup of copper inside cells can cause the clustering of mitochondrial lipoproteins and the destabilization of Fe-S cluster proteins, leading to a specific type of cell demise known as Cuprotosis.([Tang et al., 2022](#_ENREF_312)) | **2022** The study of copper death is currently focused on bioinformatics analysis of tumors and Alzheimer's disease, and no cell or animal experiments, or clinical trials have been conducted yet.([Lv et al., 2022](#_ENREF_227)) An article entitled "Copper Homeostasis and Cuproptosis in Health and Disease" highlights the importance of copper homeostasis and cuproptosis in maintaining the balance of copper levels in the body, and its potential implications in disease.([Chen et al., 2022c](#_ENREF_49)) | **2016** Studies have shown that copper levels are elevated in patients who have had a stroke.([Lai et al., 2016](#_ENREF_193))  **2022** Nevertheless, the specific association between Cuprotosis and stroke has not yet been extensively investigated. |
| Star signaling pathway | PI3K/AKT signaling pathway | **1988**Phosphatidylinositol 3-kinase (PI3K) is an enzyme that generates phosphatidylinositol lipids in cells, which can stimulate cell growth and division.([Whitman et al., 1988](#_ENREF_354))  **1995-1997**Researchers have discovered that PI3K can activate tumor suppressor kinase (AKT), which is another enzyme. AKT is a protein kinase that impedes apoptosis, and stimulates cell growth and division.([Burgering and Coffer, 1995](#_ENREF_32), [Franke et al., 1995](#_ENREF_101), [Khwaja et al., 1997](#_ENREF_177)) | **2000-2002**The PI3K/AKT signaling pathway is regulated and carried out by three Akt isoforms (Akt1, Akt2, Akt3), which mediate many downstream pathways. The regulation and conduction mechanism of this signaling pathway has been elucidated.([Cantley, 2002](#_ENREF_36))  **2002** Research has demonstrated that the PI3K/AKT signaling pathway is frequently activated in various types of tumors, and its activation may contribute to tumor growth, metastasis, and resistance to drugs.([Cantley, 2002](#_ENREF_36))  **2004**Numerous studies have shown that the activation of the PI3K/AKT signaling pathway is a prevalent characteristic of many tumor types, and its activation is linked to the facilitation of tumor growth, metastasis, and resistance to various drugs.([Fresno Vara et al., 2004](#_ENREF_102))  **2005-2020**The involvement of the PI3K/AKT signaling pathway in various diseases, such as cardiovascular diseases, diabetes, obesity, and neurodegenerative diseases, suggests its significant implications. Therefore, it has been identified as a promising therapeutic target for the treatment of several diseases.([Ghigo et al., 2017](#_ENREF_108), [Durrant and Hers, 2020](#_ENREF_89), [Zhao et al., 2021](#_ENREF_403), [Xu et al., 2020](#_ENREF_364))  **2022**The overactivation of the PI3K/AKT signaling pathway, triggered by certain factors, can result in abnormal cell growth and ultimately lead to the development of tumors. Consequently, investigating the involvement of this pathway in tumorigenesis has become a crucial area of focus in cancer research.([Klempner et al., 2013](#_ENREF_185)) | The PI3K-AKT pathway is implicated in oxidative stress, with activated AKT providing protection against oxidative damage following stroke via Nrf2/ARE effectors.([Chan, 2005](#_ENREF_41))  Moreover, the activation of the PI3K/AKT signaling pathway has been shown to have anti-inflammatory effects by suppressing the expression of pro-inflammatory factors induced by NF-κB. It also plays a role in regulating cell apoptosis or survival following transient focal cerebral ischemia.([Xian et al., 2021](#_ENREF_360), [Chen et al., 2020](#_ENREF_51))  Furthermore, PI3K/AKT signaling promotes VEGF production to induce angiogenesis after stroke, while also playing a crucial role in autophagy through the mTOR pathway.([Chen et al., 2019a](#_ENREF_48))  mTOR can both activate and inhibit autophagy in the context of ischemic stroke.([Yang et al., 2021](#_ENREF_370)) |
|  | JAK/STAT signaling pathway | **1958**Alick Isaacs and Jean Lindenmann's discovery of "viral interference" sparked research into how cells respond to IFN production and defend against viruses. This led to the study of JAK/STAT signaling, which is crucial for the antiviral response of cells.([Isaacs and Burke, 1958](#_ENREF_156))  **1990**Novel families of TYK were discovered, such as TYK2, JAK1, and JAK2. The name JAK was inspired by the Roman god Janus, who is depicted as having two faces, reflecting the characteristic two-sided structure of the kinase family.  **1992-1993**The Darnell laboratory successfully purified STAT proteins (including STAT1 and STAT2 as well as interferon regulatory factor 9). | **1994**Cytokine receptors bind to JAKs, causing activation and phosphorylation of tyrosine residues in the JAK receptor tail. This creates cytoplasmic STAT binding sites that become part of the receptor complex through autophosphorylation and binding to the SH2 domain.([Darnell et al., 1994](#_ENREF_74), [Greenlund et al., 1994](#_ENREF_114))  **2000-2008**The JAK/STAT signaling pathway participates in numerous cellular processes, such as cell proliferation, metabolism, movement, immune response, defense against infections, inflammatory response, and cancer.([Schindler, 2002](#_ENREF_282), [O'Shea and Murray, 2008](#_ENREF_257))  **2019-2022**At present, eleven JAK inhibitors have been approved for treating autoimmune diseases like rheumatoid arthritis, psoriasis, atopic dermatitis, graft-versus-host disease, and myelofibrosis. In addition, they have also been approved for the treatment of cancer, alopecia areata, and other ailments.([Barrat et al., 2019](#_ENREF_22), [Gracey et al., 2020](#_ENREF_112), [Agashe et al., 2022](#_ENREF_2))  **2022** One of the most significant challenges today is to comprehend how cells employ a limited number of JAK and STAT proteins to relay signals from numerous cytokines. The JAK-STAT pathway possesses various properties that may aid in solving this biological conundrum.([Philips et al., 2022](#_ENREF_266)) For further elaboration, refer to "The JAK-STAT Pathway at Twenty" (2012) and "The JAK-STAT pathway at 30: Much learned, much more to do" (2022).([Stark and Darnell, 2012](#_ENREF_301), [Philips et al., 2022](#_ENREF_266)) | **2001-2002** Basal brain activity and selective activation of JAK-STAT have been observed after cerebral ischemia, and this pathway is involved in the astrocyte response to focal cerebral ischemia.([Justicia et al., 2000](#_ENREF_169))Following cerebral ischemia, STAT3 has been discovered to participate in the inflammatory process.([Suzuki et al., 2001](#_ENREF_306))  **2016**The induction of VEGF expression through the JAK2/STAT3 signaling pathway is one of the mechanisms by which catalpol exerts its effects on neuroprotection and angiogenesis after a stroke.([Dong et al., 2016](#_ENREF_85))  **2017** Activation of the JAK2/STAT3 signaling pathway by nicotine has been observed to offer protection against cell apoptosis caused by cerebral ischemia/reperfusion injury.([Hu et al., 2017](#_ENREF_142))  **2018-2020**The JAK/STAT signaling pathway has been shown to have neuroprotective effects in stroke by reducing the inflammatory response and improving neurological deficits.([Ding et al., 2019b](#_ENREF_82)) |
|  | AMPK signaling pathway | The involvement of AMP in glycogen phosphorylation was discovered as far back as 1930, but the research was hindered by experimental artifacts. Fortunately, the discovery of acetyl-CoA oxygenase and HMG-CoA reductase helped to overcome these challenges and advance the research in this field.  **1987-1988**AMP-dependent protein kinase (AMPK) was discovered and named by Sim and D G Hardie, among others.([Carling et al., 1987](#_ENREF_37), [Munday et al., 1988](#_ENREF_245), [Sim and Hardie, 1988](#_ENREF_297)) | **2000**Human AMPK is a crucial heterotrimeric protein made up of three subunits: α, β, and γ. It serves as a pivotal regulator of energy metabolism, functioning as a switch that orchestrates cellular responses to energy stress and mitochondrial damage. By doing so, AMPK plays a central role in maintaining cellular energy homeostasis.([Kemp et al., 1999](#_ENREF_176))  **2001**Metformin, a first-line drug for diabetes, has been found to inhibit AMPK signaling pathway, which is a novel mechanistic finding.([Zhou et al., 2001](#_ENREF_405), [Fu et al., 2013](#_ENREF_103))  **2010-2020**The AMPK signaling pathway serves various functions in the cell, including regulating cell metabolism, responding to cell stress, controlling autophagy, and maintaining mitochondrial homeostasis. Recent research has highlighted the central role of the AMPK signaling pathway in immune inflammation, aging, cancer, and diabetes.([Grahame Hardie, 2016](#_ENREF_113), [Herzig and Shaw, 2018](#_ENREF_132), [O'Neill and Hardie, 2013](#_ENREF_256), [Salminen and Kaarniranta, 2012](#_ENREF_277))  **2022**Additionally, the potential of the AMPK signaling pathway as a target for cancer therapy has been extensively studied, with various strategies proposed for targeting this pathway in cancer treatment.([Hsu et al., 2022](#_ENREF_141)) | **2005-2007**In a focal stroke model, AMPK activation has been found to be detrimental.([McCullough et al., 2005](#_ENREF_235)) Therefore, pharmacological inhibition of AMPK has been suggested as a means of mediating neuroprotective effects.([Li et al., 2007](#_ENREF_208))  **2010-2015**Inhibition of AMPK has been shown to reduce infarct size, apoptosis, and improve neurological function, ultimately exerting neuroprotective effects.([Jia et al., 2015](#_ENREF_163), [Jiang et al., 2015](#_ENREF_166)) Studies have also demonstrated that the AMPK inhibitor metformin can reduce blood-brain barrier disruption in a stroke model.([Liu et al., 2014](#_ENREF_222))  **2016-2020** AMPK-mediated autophagy,neuroinflammation, apoptosis and angiogenesis contribute to neuroprotection in cerebral ischemia, suggesting a potential strategy for future stroke treatment.([Jiang et al., 2018b](#_ENREF_165)) |
|  | MAPK signaling pathway | **1982**Cooper's research showed that platelet-derived growth factor and epidermal growth factor can lead to the phosphorylation of specific tyrosine residues in cellular proteins.([Cooper et al., 1982](#_ENREF_66))  **1988-1989**Ray et al. named MAPKs and synthesized previous studies to establish the MAPK signaling pathway.([Ray and Sturgill, 1988](#_ENREF_270), [Boulton et al., 1990](#_ENREF_26))  **1990**Boulton was the first to clone the cDNA encoding MAPKs.([Boulton et al., 1990](#_ENREF_26)) | **1993-1995**The MAPK signaling pathway can be summarized as follows: extracellular signal → membrane receptor → RAS → MAP3K → MAP2K → MAPK, which subsequently activates downstream target genes. The key pathways in this process are the ERK kinase and JNK signaling pathways.([Seger and Krebs, 1995](#_ENREF_285), [Davis, 1994](#_ENREF_77))  **1995-2000**The MAPK pathway can be subdivided into four primary branches: ERK, JNK, p38/MAPK, and ERK5.([Cano and Mahadevan, 1995](#_ENREF_35))  **2002-2020**The MAPK signaling pathway plays a critical role in a wide range of cellular functions, such as proliferation, differentiation, apoptosis, and survival. Furthermore, this pathway is implicated in various physiological processes, including inflammation, apoptosis, and cancer development.([Boutros et al., 2008](#_ENREF_27), [Wagner and Nebreda, 2009](#_ENREF_332), [Bermudez et al., 2010](#_ENREF_24))  **2022**Understanding the MAPK signaling pathway and its mechanisms is crucial for developing targeted intervention drugs for related diseases. This knowledge can offer new opportunities for treating malignant tumors, as well as acute and chronic inflammatory conditions, including cytokine storms and rheumatoid arthritis.([Ronkina and Gaestel, 2022](#_ENREF_276)) | **1999** Inhibition of the p38-MAPK pathway can decrease the production of inflammatory mediators and cytokines, while the ERK1/2 pathway has been shown to protect neurons and oligodendrocytes. These findings suggest that targeting these pathways may hold promise as a potential approach for treating stroke.([Barone and Feuerstein, 1999](#_ENREF_20), [Irving et al., 2000](#_ENREF_155), [Barone et al., 2001](#_ENREF_21))  **2008**The ERK1/2 pathway exhibits a dual effect following stroke, and inhibition of the MAPK pathway has been shown to reduce neuroinflammation following ischemic stroke.([Sawe et al., 2008](#_ENREF_281), [Fann et al., 2018](#_ENREF_93))  **2021**Studies have demonstrated that baicalin has the potential to activate both the PI3K/AKT and MAPK signaling pathways, resulting in improvements in oxidative stress, apoptosis, and inflammation.([Li et al., 2021a](#_ENREF_206)) |

Table 4: Summarize the historical process, advantages and disadvantages of existing major western medical treatments for stroke([Powers et al., 2019](#_ENREF_268))

| **Classification of treatments** | **Methods** | **Course of history** | **Advantages** | **Disadvantages** | **References** |
| --- | --- | --- | --- | --- | --- |
| Vascular interventional therapy | Mechanical thrombectomy | **2013**The clinical trials for mechanical thrombectomy did not yield positive results.  **2015** Recommended by clinical guidelines, mechanical thrombectomy technology has developed rapidly.  **2020** New technology and the use of artificial intelligence have given mechanical thrombectomy new capabilities. | Methods for saving lives and improving the course of acute ischemic stroke. | bleeding, edema, reocclusion, and complications related to accessing the site. | Krishnan, R. et al.([Krishnan et al., 2021](#_ENREF_187)) |
|  | Arterial thrombolysis | Intra-arterial thrombolysis involves using catheter technology to administer thrombolytic drugs directly to the site of the clot, facilitating its dissolution.  **2020** The advancements in new technology and artificial intelligence have given rise to new capabilities. | Improve the recanalization rate | Currently, there is insufficient evidence to confirm the benefits of intra-arterial thrombolysis. | Hlavica, M. et al.([Hlavica et al., 2015](#_ENREF_136)) |
|  | Angioplasty | **1995** Angioplasty can be utilized to treat secondary vasospasm in stroke cases. | Improved cerebral blood flow | The safety and efficacy of angioplasty for stroke treatment remain unclear, and the procedure carries a high risk. | Higashida, R. T. et al.([Higashida et al., 1995](#_ENREF_133))  Wabnitz, A. et al.([Wabnitz and Chimowitz, 2017](#_ENREF_331)) |
| Intravenous thrombolytic therapy | Urokinase | **1951**Willams discovered urokinase in urine.  **1978**Urokinase has been shown to be safe and effective in treating ischemic stroke in both phase I and phase II clinical trials conducted in China. | Urokinase has a comparable safety profile to alteplase and has been shown to decrease the incidence of stroke. | The clinical studies have a limited basis, are non-selective, and indicate a risk of severe bleeding. | Furlan, A. et al.([Furlan et al., 1999](#_ENREF_106))  Wardlaw, J. M. et al.([Wardlaw et al., 2014](#_ENREF_348))  Wang, X. et al.([Wang et al., 2021](#_ENREF_345)) |
|  | rt-PA | **1979**Rijken et al. were successful in discovering t-PA, which marked the beginning of a new era in thrombolytic therapy.  **1987-1995**The ECASS rt-PA study established the cornerstone of the conduct of the trial.  **1995-2020** Explain the practical value of using rt-PA in clinical applications with specific actions. | Using rt-PA to treat acute cerebral infarction is a classic, effective, and safe treatment method that can improve patient outcomes. | Potential risks of using rt-PA include bleeding and vasogenic edema. | Rijken, D. C. et al.([Rijken and Collen, 1981](#_ENREF_273))  Foster-Goldman, A. et al.([Foster-Goldman and McCarthy, 2013](#_ENREF_99))  Wardlaw, J. M. et al.([Wardlaw et al., 2014](#_ENREF_348)) |
|  | TNK-tPA  (Tenecteplase) | **2000** TNK-tPA is a third-generation thrombolytic agent that was developed..  **2005** The clinical study of TNK-tPA was started.  **2020** There is controversy over whether to move beyond rt-PA. | TNK-tPA has a similar safety profile to alteplase, but is available at a lower cost. | There is debate regarding whether to progress beyond the use of rt-PA. | Lapchak, P. A. et al.([Lapchak, 2002](#_ENREF_195))  Hlavica, M. et al.([Hlavica et al., 2015](#_ENREF_136))  Singh, N. et al.([Singh et al., 2023](#_ENREF_298)) |
| Anti platelet agents | Aspirin | **1897-1899**Aspirin, which was synthesized by Hofmann in Germany, is a type of synthetic acetylsalicylic acid.  **1980**The FDA has approved aspirin for secondary prevention of transient ischemic attack (TIA) or stroke.  **2020** The use of aspirin may lead to resistance and potentially cause kidney and liver damage. | Decreasing the likelihood of recurrent strokes and death. | Bleeding, kidney injury, liver injury, and aspirin resistance | Greer, D. M. et al.([Greer, 2010](#_ENREF_115))  Minhas, J. S. et al.([Minhas et al., 2022](#_ENREF_240))  Cochrane, A. et al.([Cochrane et al., 2023](#_ENREF_63)) |
| Anticoagulation | | Although acute-phase anticoagulation has been used for over 60 years, its use remains a topic of controversy. | Anticoagulation therapy has the potential to reduce the risk of recurrent stroke and may be a safe and effective option. | It is important to carefully consider the potential risks, such as an increased risk of bleeding. | Sandercock, P. A. et al.([Sandercock et al., 2008](#_ENREF_278)) |
| Defibration | Defibrase | **1894** Kraemer and Spilker were the first to discover enzymes capable of breaking down cellulose.  **1940-1960** the mechanism of defibrinogenation was discovered, and defibrinogen was extracted and developed.  **The 1970s** Scientists have been continuously improving the production technology and degradation mechanism of defibrinogen, and discovering new defibrinogens. | Reduce the recurrence rate of stroke | There may be a tendency to bleed | Cooperative Group for Reassessment([Cooperative Group for Reassessment of, 2005](#_ENREF_67)) |
|  | Batroxobin | Batroxobin is the generic name given by the World Health Organization (WHO) to the fibrin coagulant protease found in the venom of Bothrops atrox. | For acute ischemic cerebrovascular disease and sudden deafness, Batroxobin has shown effective hemostatic properties. | Common adverse effects of the treatment include bleeding, as well as gastrointestinal symptoms such as nausea, vomiting, and diarrhea, and headache. | Ding, J. Y. et al.([Ding et al., 2019a](#_ENREF_80)) |
|  | Other | The clinical application of lumbrokinase and agkistrodon acutus enzyme requires further study. | / | / | / |
| Expansion of capacity | | / | / | Targeted, the effect is controversial | Chang, T. S. et al.([Chang and Jensen, 2014](#_ENREF_43)) |
| Dilation of blood vessels | | / | / | Large sample clinical trials are needed | / |
| Statins | | / | There may be no adverse effects | There is currently insufficient clinical evidence to support the effectiveness of this intervention. | Tsivgoulis, G. et al([Tsivgoulis et al., 2015](#_ENREF_320)) |
| Neuroprotective Drugs | Edaravone | **1989** Researchers at Osaka City University in Japan discovered Edaravone.  **1999** It was approved in Japan as the first neuroprotective drug for the treatment of acute ischemic stroke.  **2017** Edaravone received FDA approval for the treatment of amyotrophic lateral sclerosis. | Inhibiting ferroptosis and scavenging free radicals; Preventing cerebral infarction and its progression;  Inhibition of nerve death. | Abnormal liver function, disseminated intravascular coagulation (DIC), and renal failure. | Lapchak, P. A. et al.([Lapchak, 2010](#_ENREF_196))  Cochrane Database Syst Rev et al.([Feng et al., 2011](#_ENREF_95))  Homma, T. et al.([Homma et al., 2019](#_ENREF_138)) |
|  | Citicoline | **1982** Researchers discovered citicoline in lecithin.  **2005** It was approved by the FDA for clinical trials in the treatment of stroke and brain injury.  **NOW** Citicoline has become increasingly used in the field of neuroprotection and repair. | It can promote brain function recovery and protection. | Shock, insomnia, rash, limited therapeutic effect | Dávalos, A. et al.([Dávalos and Secades, 2011](#_ENREF_75))  Martí-Carvajal, A. J. et al.([Martí-Carvajal et al., 2020](#_ENREF_230)) |
| Other Medications | DL-3-n-butylphthalide | Drugs developed in China for the treatment of ischemic stroke | Increased angiogenesis and cerebral blood flow in the ischemic area | Bleeding tendency, insufficient evidence | Wang, H. et al.([Wang et al., 2022b](#_ENREF_339)) |
|  | Human urinary kallidinogenase | Class I chemical new drugs developed in China in recent years | It can improve cerebral arterial circulation, reduce nerve function damage, and has good safety and tolerance. | Transient hypotension | Zhang, C. et al.([Zhang et al., 2012](#_ENREF_391))  Ni, J. et al.([Ni et al., 2021](#_ENREF_248)) |
| Other Therapies | Oxygen treatment | / | / | There was no benefit to the outcome | / |

Table 5: Summarize the current status of western medicine in the research and development stage in the treatment of stroke

| **Aspects** | **Pathway** | **Western medicine** | **Type of study or model** | **Results** | **References** |
| --- | --- | --- | --- | --- | --- |
| Angiogenesis | Rho/Rock signaling pathway | Fasudil | Animal experiments and clinical trials | Fasudil, a ROCK inhibitor, has been shown to improve neurological function and clinical outcomes in patients with ischemic stroke. The drug has been approved for human use in Japan and China since 1995. However, caution should be exercised as there have been reports of side effects, including hypotension, intracranial hemorrhage, and liver and kidney dysfunction. | Rikitake et al.([Rikitake et al., 2005](#_ENREF_274))  Shimokawa et al.([Shimokawa and Takeshita, 2005](#_ENREF_293))  Shibuya et al.([Shibuya et al., 2005](#_ENREF_290)) |
|  | Wnt/β-catenin signaling pathway | Dickkopf-1 | MCAO | Dkk-1 is induced in neurons located in both the ischemic core and penumbral zone, and antagonists of Dkk-1 have shown potential in protecting against stroke. | Mastroiacovo, F et al.([Mastroiacovo et al., 2009](#_ENREF_231)) |
|  |  | TWS3 | MCAO | TSW119, a GSK-3β inhibitor, activates the Wnt/β-catenin signaling pathway and attenuates the RT-PA-induced blood-brain barrier disruption. | Wang W et al.([Wang et al., 2016b](#_ENREF_344)) |
| Oxidative stress | SHH signaling pathway | Guanxinning | OGD/R | Axon-directed signals can prevent damage from oxygen-sugar deprivation/reoxygenation, promote axon growth, and stimulate synaptogenesis in damaged neurons. | Xiao, G et al.([Xiao et al., 2021](#_ENREF_361)) |
|  | Nrf2/ARE signaling pathway | Dimethyl Fumarate | ICH model | Nrf2 knockout mice showed a reduced incidence of hematoma, an extended treatment time window, and decreased brain edema. | Zhao, X et al.([Zhao et al., 2015](#_ENREF_401)) |
|  |  | tBHQ | MACO/R | Nrf2 knockout mice exhibited decreased brain damage, cortical damage, and sensorimotor loss. | Shih, A. Y. et al.([Shih et al., 2005](#_ENREF_291)) |
|  | HIF-1α signaling pathway | IOX3 | MACO/R | Elevated HIF-1α levels upregulate EPO expression, leading to reduced blood-brain barrier disruption and cerebral infarction. | Chen, R. L. et al.([Chen et al., 2014](#_ENREF_50)) |
|  |  | DPP-4 | Cerebral microvascular endothelial cell ischemia model | Promotion of cell proliferation and migration in injured tissue is associated with increased levels of VEGF, eNOS, HIF-1α, and SIRT1. | Mi, D. H. et al.([Mi et al., 2019](#_ENREF_239)) |
|  |  | Empagliflozin | I/R | Administration of the treatment after cerebral ischemia can reduce infarct size, prevent neuronal death, and enhance neural function, possibly through the upregulation of VEGF and HIF-1α. | Abdel-Latif, R. G. et al.([Abdel-Latif et al., 2020](#_ENREF_1)) |
|  |  | Fluoxetine | tMACO/R | Upregulation of the HIF-1α/VEGF pathway was observed, which was associated with improved neural function. | Hu, Q. et al.([Hu et al., 2020](#_ENREF_143)) |
|  | PPAR-γ signaling pathway | RSG | MACO/R, clinical trials | PPAR-γ activation has been shown to improve neurological function, reduce pro-inflammatory cytokine release such as TNF-α and IL-6, and attenuate the inflammatory response. Moreover, it may mitigate the injury caused by recurrent stroke and facilitate the transformation of microglia to an anti-inflammatory phenotype. | Culman, J.([Culman et al., 2007](#_ENREF_73))  Li, Y.([Li et al., 2019](#_ENREF_215)) |
| Autophagy | Bnip3 signaling pathway | Roxadustat | MACO/R, OGD/R | The HIF-1α/Bnip3 signaling pathway can enhance autophagy levels, inhibit apoptosis following cerebral ischemia, and promote nerve recovery. | Chen, J. et al.([Chen et al., 2022b](#_ENREF_46)) |
|  | P53 signaling pathway | Pifithrin α | tMACO | Inhibition of the binding of the P53 gene to DNA sites can reduce the expression of the related gene p21, which in turn reduces apoptosis and promotes neurogenesis. | Leker, R. R. et al.([Leker et al., 2004](#_ENREF_199))  Luo, Y. et al.([Luo et al., 2009](#_ENREF_226)) |
| Inflammation | NF-κB signaling pathway | Dexmedetomidine | ICH model | Reducing the expression of inflammatory cytokines and protecting the integrity of the blood-brain barrier can be achieved by inhibiting the NF-κB signaling pathway. | Guo, H. et al.([Guo et al., 2022](#_ENREF_119)) |
| Apoptosis | Notch signaling pathway | Atorvastatin | MACO | In addition, it upregulated the expression of PS1 and Notch signaling, thereby promoting the proliferation of neural precursor cells in the subventricular zone. | Chen, J. et al.([Chen et al., 2008](#_ENREF_47)) |
|  |  | Simvastatin | MCAO | Upregulating the expression of PS1 and Notch signaling promotes arterial cell migration and thereby enhances angiogenesis. | Zacharek, A. et al.([Zacharek et al., 2009](#_ENREF_385)) |
|  | Hippo signaling pathway | Verteporfin | MCAO/R | Inhibiting the nuclear translocation of YAP can improve the integrity of the blood-brain barrier. | Gong, S. et al.([Gong et al., 2021](#_ENREF_110)) |
|  |  | Dexamethasone | I/R | Reducing brain injury and infarct size, improving nerve function, and decreasing blood-brain barrier permeability are potential benefits of this treatment after stroke. | Gong, P. et al. ^[53]^ |
|  | TGF-β/Smad signaling pathway | isoflurane | MACO/R | Upregulating TGF-β1 and Smad3 expression and downregulating p-JNK expression are potential mechanisms that can reduce brain injury. | Wang, S. et al.([Wang et al., 2016a](#_ENREF_343)) |
| Ferroptosis signaling pathway | | Selenium | ICH model | Pharmacological Se has been shown to effectively inhibit GPX4-dependent ferroptosis and ER stress-induced cell death. | Alim, I. et al.([Alim et al., 2019](#_ENREF_10)) |
|  |  | DFO | MCAO/R | Intranasal administration has been shown to reduce infarct size, decrease neuronal death, and has been approved for the treatment of iron overload. | Hanson, L. R. et al.([Hanson et al., 2009](#_ENREF_120)) |
| Star signaling pathway | PI3K/AKTsignaling pathway | Selenium | MCAO/R | The PI3K/AKT/mTOR signaling pathway has been shown to activate cellular processes that inhibit autophagy, decrease blood-brain barrier permeability, and increase the expression of tight junction proteins. | Yang, B. et al.([Yang et al., 2021](#_ENREF_370)) |
|  | JAK/STAT signaling pathway | Ruxolitinib | MCAO, OGD/R | The inhibition of JAK2/STAT3 pathway activation and NLRP3 inflammasome secretion has been found to reduce the production of pro-inflammatory cytokines, such as TNF-α, IFN-γ, HMGB1, IL-1β, IL-2, and IL-6. | Zhu, H. et al.([Zhu et al., 2021](#_ENREF_409)) |
|  | AMPK signaling pathway | Metformin | tMCAO/R | The activation of AMPK phosphorylation and the inhibition of NF-κB activation have been shown to reduce the incidence of stroke and the levels of pro-inflammatory cytokines, such as IL-6, IL-1β, TNF-α, and ICAM-1. | Liu, Y. et al.([Liu et al., 2014](#_ENREF_222)) |
|  |  | Apelin 13 | In vivo I/R and PC12 in vitro models | Activation of the AMPK/GSK-3β/Nrf2 pathway can inhibit inflammation and oxidative stress. | Duan, J. et al.([Duan et al., 2019](#_ENREF_87)) |
|  |  | Ezetimibe | MCAO | Activation of AMPK/Nrf2 pathway can lead to upregulation of HO-1, which acts as an antioxidant and reduces oxidative stress. Meanwhile, it can also lead to increased expression of NLRP3, Caspase-1, and IL-1β, which are related to inflammatory response. | Yu, J. et al.([Yu et al., 2020a](#_ENREF_378)) |
|  | MAPK signaling pathway | maraviroc | MCAO, OGD/R | It has been employed for the treatment of HIV and to decrease the area of cerebral infarction. It works by increasing the expression of IL-1β, IL-6, and TNF-α while inhibiting the NF-κB signaling pathway, thereby exhibiting anti-inflammatory and anti-apoptotic effects. | Chen, B. et al.([Chen et al., 2022a](#_ENREF_44)) |

Table 6: Summarize the current research on TCM and its active ingredients for stroke treatment

| **Aspects** | **Pathway** | **TCM** | **Model** | **Results** | **References** |
| --- | --- | --- | --- | --- | --- |
| Angiog-enesis | Rho/Rock signaling pathway | Nolo Xintong | MCAO/R | The production and expression levels of the Nogo-A/Rho/Rock signaling pathway were reduced, resulting in an anti-cerebral ischemic effect, along with a decrease in the methylation level of Nogo-A. | Hong L et al.([Hong et al., 2021](#_ENREF_139)) |
|  | Wnt/β-catenin signaling pathway | Morroniside | MCAO/R | Activating the Wnt/β-catenin signaling pathway enhances neural stem cell proliferation, neural expression, and upregulation of the T cell transcription factor-4 (TCF-4). | Fang-Ling Sun et al.([Sun et al., 2014](#_ENREF_304)) |
|  |  | Cornin | MCAO/R | The regulation of angiogenesis and enhancement of functional recovery following stroke are achieved through the activation of the Ang 1/Tie 2 axis and Wnt/β-catenin signaling pathway. | Yangyang Xu et al.([Xu et al., 2016](#_ENREF_366)) |
|  |  | XQ-1H | MCAO/R | The activity of GSK-3β/Caspase3 decreased, resulting in an increase in the expression of proteins related to neurogenesis. This inhibition of apoptosis is associated with an upregulation of Wnt, β-catenin, Bcl-xl, and Cyclin D1. | Dan Xu et al.([Xu et al., 2019](#_ENREF_363)) |
|  |  | Astragaloside IV | Photochemical ischemia model | Promote neurogenesis and proliferation of neural stem cells after stroke | Sun L et al.([Sun et al., 2020](#_ENREF_305)) |
| Oxidative stress | SHH signaling pathway | Resveratrol | MCAO/R | Activation of the SHH pathway and the upregulation of Gli-1 protein expression have been shown to promote nerve cell and oligodendrocyte activation, decrease brain damage, and improve neurological function. | Yu P et al.([Yu et al., 2017](#_ENREF_381), [Yu et al., 2021](#_ENREF_380)) |
|  | Nrf2/ARE signaling pathway | Withaferin A | ICH model | Increasing the translocation of Nrf2 into the nucleus can enhance the expression of HO-1, resulting in decreased brain injury and oxidative stress following brain damage. | Zhou, Z. X et al.([Zhou et al., 2023](#_ENREF_408)) |
|  |  | Rosmarinic acid | MCAO | By reducing cell apoptosis, increasing the expression levels of anti-apoptotic protein Bcl-1, heme oxygenase-1 (HO-1), nuclear factor erythroid 2-related factor 2 (Nrf2), and superoxide dismutase (SOD), while decreasing the expression level of pro-apoptotic protein Bax, it can decrease the area of cerebral infarction. | Cui, H. Y. et al.([Cui et al., 2018](#_ENREF_71)) |
|  |  | Plumbagin | MCAO/R | Activation of the Nrf2/ARE signaling pathway can reduce brain damage and improve neurological deficits. | Son, T. G et al.([Son et al., 2010](#_ENREF_299)) |
|  |  | 11-Keto-β-boswellic acid | MCAO/R, OGD/R | KBA administration reduced the ischemia-reperfusion-induced cerebral infarction area, lowered malondialdehyde (MDA) levels, and increased superoxide dismutase (SOD) activity. Furthermore, KBA upregulated the expression of Nrf2 and HO-1. | Ding, Y. et al.([Ding et al., 2015](#_ENREF_81)) |
|  |  | Baicalin | tMCAO, pMCAO | The Nrf2-HO-1 signaling pathway activation can reduce the production of reactive oxygen species (ROS) and alleviate brain injury. | Huang, Z. et al.([Huang et al., 2021a](#_ENREF_150)) |
|  |  | Curcumin | MCAO/R | Upregulation of Nrf2 and its downstream antioxidant genes can inhibit oxidative stress and promote the recovery of brain nerve function, which ultimately reduces brain injury. This process is associated with a decrease in the levels of reactive oxygen species (ROS) and malondialdehyde (MDA). | Duan, C. et al.([Duan et al., 2022](#_ENREF_86)) |
|  |  | Geraniol | MCAO/R, OGD/R | The activation of the Nrf2/ARE signaling pathway can reduce cell apoptosis by inhibiting oxidative stress, which results in an increase in the expression levels of SOD, NO, nNOS, Nrf2, HO-1, and a decrease in the levels of MDA. | Yang, Y et al.([Yang et al., 2022](#_ENREF_374)) |
|  | HIF-1α signaling pathway | Catalpol | MCAO/R, OGD/R | The HIF-1α/VEGF signaling pathway can enhance brain recovery, stimulate the proliferation and migration of brain microvascular endothelial cells, and facilitate angiogenesis to improve brain injury. | Wang, H et al.([Wang et al., 2020](#_ENREF_338)) |
| Autophagy | Pink1/Parkin signaling pathway | Ligustractone | MCAO/R, OGD/R | The Pink1/Parkin signaling pathway promotes mitophagy and reduces neuronal damage. | Mao, Z. et al.([Mao et al., 2022](#_ENREF_229)) |
|  | Bnip3 signaling pathway | Hydroxysafflor Yellow A | OGD/R | The activation of the HIF-1α/BNIP3 signaling pathway induces autophagy and leads to a reduction in apoptosis, with a consequent decrease in CASP3 and an increase in HIF-1α levels. | Wei, R.([Wei et al., 2022](#_ENREF_349)) |
|  | P53 signaling pathway | rosmarinic acid n-butyl ester | OGD/R | The treatment exerts its anti-ischemic effect by decreasing the apoptosis rate and suppressing the expression of Bax, p53, TNF-α, IL-1β, NO, PGE(2), iNOS, and COX-2, and by increasing the expression of DAPK. | Wu, L.([Wu et al., 2017b](#_ENREF_357)) |
| Inflammation | NF-κB signaling pathway | Pterostilbene | MCAO/R, OGD/R | Inhibition of NF-κB targets can help to reduce inflammation and oxidative damage. This leads to a decrease in the levels of MDA, ROS, TNF-α, IL-1β, and IL-6, while increasing the levels of SOD. | Liu, H. et al.([Liu et al., 2019](#_ENREF_220)) |
|  |  | Curcumin | MCAO/R | The suppression of the NF-κB signaling pathway activation leads to a decrease in the expression levels of GSDMD-N, cleaved caspase-1, NLRP3, IL-1β, and IL-18, which results in a reduction in inflammation and oxidative damage. | Ran, Y. et al.([Ran et al., 2021](#_ENREF_269)) |
|  |  | Syringin | MCAO/R | Reduced nuclear translocation of NF-κB can attenuate brain injury and decrease levels of NF-κB, IL-1β, IL-6, TNF-α, and MPO. | Tan, J. et al.([Tan et al., 2021](#_ENREF_310)) |
|  |  | Xueshuantong injection | MCAO/R | Inhibition of JAK, STAT3, IκB, NF-κB, and JNK phosphorylation can effectively suppress the inflammatory response, and improve vascular structural damage. | Wang, G. et al.([Wang et al., 2022a](#_ENREF_335)) |
|  |  | Salvianolic Acid D | MCAO/R | Promoting the translocation of HMGB1 from the nucleus to the cytoplasm can inhibit the TLR4/MyD88/NF-κB signaling pathway, resulting in an anti-inflammatory effect. This leads to an upregulation of Bcl-2 expression and a downregulation of Bax and cleaved-caspase-3 levels. | Zhang, W. et al.([Zhang et al., 2020](#_ENREF_393)) |
|  | IL-33/ST2 signaling pathway | Celastrol | MCAO/R, OGD/R | Activation of the IL33/ST2 axis mediates microglia/macrophage polarization, resulting in reduced expression of inflammatory factors. This promotes an anti-inflammatory response and facilitates tissue recovery. | Jiang, M. et al.([Jiang et al., 2018a](#_ENREF_164)) |
| Apoptosis | Notch signaling pathway | Astragaloside IV | MCAO/R | Activation of the HIF/VEGF/Notch signaling pathway increases miRNA-210 expression, promoting angiogenesis and cell proliferation, while significantly reducing the size of the infarct. | Liang, C. et al.([Liang et al., 2020](#_ENREF_218)) |
| Ferroptosis signaling pathway | | Baicalin | ICH model and PC12 cell | Enhanced cell viability, inhibited ferroptosis, and alleviated brain damage | Duan, L. et al.([Duan et al., 2021](#_ENREF_88)) |
|  |  | Rehmanosine A | MCAO/R | Activation of the PI3K/AKT/Nrf2 and SLC7A11/GPX4 signaling pathways has been shown to improve cognitive dysfunction following cerebral ischemia, protect nerves, and inhibit ferroptosis. | Fu, Y. et al.([Fu et al., 2022](#_ENREF_104)) |
|  |  | Baicalein | tMCAO/R, OGD/R | This treatment modulates the GPX4/ACSL4/ACSL3 axis to inhibit ferroptosis. | Li, M. et al.([Li et al., 2022](#_ENREF_210)) |
|  |  | Astragaloside IV | SAH model | Activation of the Nrf2/HO-1 signaling pathway enhances antioxidant capacity, inhibits lipid peroxidation, and upregulates SLC7A11, GPX4, and ROS. | Liu, Z. et al.([Liu et al., 2022](#_ENREF_223)) |
|  |  | Kaempferol | OGD/R | Activation of the Nrf2/SLC7A11/GPX4 signaling pathway can decrease the levels of SLC7A11, GPX4, NADPH, GSH, and SOD, enhance antioxidant capacity, and prevent ferroptosis. | Yuan, Y. et al.([Yuan et al., 2021](#_ENREF_383)) |
|  |  | β-lycopene | MCAO/R, OGD/R | Activation of the Nrf2/HO-1 signaling pathway can prevent ferroptosis, improve neurological function, and reduce the size of the infarct. | Hu, Q et al.([Hu et al., 2022a](#_ENREF_144)) |
|  |  | Danhong injection | pMCAO/R | Activation of SATB1/SLC7A11/HO-1 signaling pathway can reduce infarct size and brain damage, and reduce ferroptosis. | Zhan, S. et al.([Zhan et al., 2022](#_ENREF_390)) |
| Star signaling pathway | PI3K/AKTsignaling pathway | Rg1 | dMCAO, OGD/R | Activation of the PI3K/AKT/mTOR, VEGF, and HIF-1α signaling pathways can increase the expression of p-PI3K, p-Akt, and p-mTOR, which enhances cell survival and promotes angiogenesis, ultimately leading to improved neurological function and reduced cerebral infarction area. | Chen, J. et al.([Chen et al., 2019a](#_ENREF_48)) |
|  |  | Aloe emodin | MCAO/R, OGD/R | The therapy decreases the levels of TNF-α, MDA, LDH, and Caspase 3, and enhances SOD activity and the Bcl-2/Bax ratio, resulting in an activation of the PI3K/AKT/mTOR signaling pathway and a inhibition of the NF-κB signaling pathway. Consequently, neurological function is improved, and the size of cerebral infarction is reduced. | Xian, M. et al.([Xian et al., 2021](#_ENREF_360)) |
|  |  | Resveratrol | MCAO | The JAK2/STAT3 and PI3K/AKT/mTOR signaling pathways can be activated to reduce the expression of cleaved caspase-3 and Bax while increasing the expression of Bcl-2, thereby decreasing neuronal apoptosis and improving neurological function, and reducing the area of cerebral infarction. | Hou, Y. et al.([Hou et al., 2018](#_ENREF_140)) |
|  |  | Baicalein | MCAO | Inhibiting the NF-κB signaling pathway can reduce levels of pro-inflammatory cytokines including IL-6, IL-18, and TNF-α, and lower caspase-3 activity, while increasing the Bcl-2/Bax ratio to reduce neuronal apoptosis. Additionally, activation of the PI3K/AKT/mTOR signaling pathway through phosphorylation can promote neuronal survival and reduce the size of cerebral infarction area. | Yang, S. et al.([Yang et al., 2019](#_ENREF_372)) |
|  |  | Puerarin | Ich-induced EBI | The possible mechanism behind the attenuation of early brain injury induced by intracerebral hemorrhage by puerarin could be the activation of the PI3K/Akt signaling pathway, which may suppress the NF-κB pathway. | Zeng, J. et al.([Zeng et al., 2021](#_ENREF_388)) |
|  |  | Salvianolic acid A | MCAO/R | Blocking the NF-κB signaling pathway can prevent the inflammatory response and upregulate the PI3K/AKT signaling pathway, leading to a reduction in GSK/3β activity and an increase in neurogenesis. Consequently, the expression levels of β-catenin and Bcl-2 are increased. | Chien, M. Y. et al.([Chien et al., 2016](#_ENREF_57)) |
|  |  | Loureirin B | MCAO/R | The treatment activates the PI3K/AKT/Nrf2 and CREB signaling pathways, which in turn increase the expression of HO-1 and Bcl-2. This leads to a reduction in cerebral infarction area, improvement in neurological function, and regulation of oxidative stress. | Xin, N. et al.([Xin et al., 2013](#_ENREF_362)) |
|  |  | DGMI | MCAO/R, OGD/R | It activates PI3K/AKT/Nrf2 and AKT/CREB signaling pathways. | Zhang, W. et al.([Zhang et al., 2018](#_ENREF_394)) |
|  |  | Panax notoginseng saponin | OGD/R | Activation of PI3K/AKT/Nrf2 signaling pathway exerts antioxidant activity and reduces the destruction of blood-brain barrier. | Hu, S. et al.([Hu et al., 2018](#_ENREF_145)) |
|  |  | Baicalin | Ischemic stroke model | Activation of PI3K/AKT signaling pathway, up-regulation of glutamate transporter 1, inhibition of cell apoptosis, reduction of cerebral infarction area and protection of rats from neuronal damage | Zhou, Z. Q. et al.([Zhou et al., 2017](#_ENREF_407)) |
|  | JAK/STATsignaling pathway | Catalpol | pMCAO | By activating the JAK2/STAT3 signaling pathway, the expression of VEGF is increased and the inhibition of STST3 nuclear translocation by AG490 is reversed. This results in both angiogenesis and neuroprotection. | Dong, W. et al.([Dong et al., 2016](#_ENREF_85)) |
|  |  | Hydroxysafflor Yellow A | MCAO | It can inhibit the activation of JAK/STAT signaling pathway in ischemic injury, regulate the crosstalk between SOCS3 signaling pathway and JAK signaling pathway, and protect neurological function. | Yu, L. et al.([Yu et al., 2020b](#_ENREF_379)) |
|  |  | Nicotiflorin | MCAO/R | The proposed treatment modality reduces cellular apoptosis, protects neurons, and suppresses immune activity, resulting in increased levels of Bcl-2 and decreased levels of pJAK-2, pSTAT-3, Bax, and Caspase-3. | Hu, G. Q. et al.([Hu et al., 2017](#_ENREF_142)) |
|  |  | Atractylenolide III | MCAO, OGD/R | The suppression of the JAK2/STAT3 signaling pathway can promote the restoration of cerebral blood flow, decrease mitochondrial fission and the phosphorylation of Drp1, as well as lower the levels of pro-inflammatory cytokines such as IL-1β, TNF-α, and IL-6. These effects can lead to a reduction in the cerebral infarction area. | Zhou, K. et al.([Zhou et al., 2019](#_ENREF_406)) |
|  |  | Stachydrine | OGD/R | The treatment resulted in improved cell survival rate, decreased levels of inflammatory factors such as IL-1β, TNF-α, SOD, and MDA, as well as inhibition of the JAK2/STAT3 signaling pathway. These effects ultimately led to an improvement in nerve function. | Li, L. et al.([Li et al., 2020](#_ENREF_209)) |
|  | AMPKsignaling pathway | Berberine | tMCAO | Activation of the AMPK pathway can lead to microglia polarization, which can improve post-stroke function and increase angiogenesis. | Zhu, J. et al.([Zhu et al., 2019](#_ENREF_410)) |
|  |  | Resveratrol | MCAO/R | ATP, phosphorylated MAPK (p-MAPK), SIRT1, and cAMP levels are increased, which contribute to the neuroprotective effects. | Wan, D. et al.([Wan et al., 2016](#_ENREF_334)) |
|  |  | Ginkgolide | OGD | It enhances autophagy and increases the expression of Beclin-1 and IL3, while decreasing the expression of p62. This is achieved by activating the AMPK/mTOR/ULK1 signaling pathway, which promotes cell proliferation and migration, and ultimately provides protection against stroke. | Zhang, Y. et al.([Zhang and Miao, 2018](#_ENREF_397)) |
|  |  | Palmatine | MCAO | Activating the AMPK/Nrf2 signaling pathway can help decrease oxidative stress, inflammation, and neuronal apoptosis. | Tang, C. et al.([Tang et al., 2021](#_ENREF_311)) |
|  |  | Pien-Tze-Huang | MCAO | To decrease the occurrence of infarction and stimulate the activity of microglia, one can activate the AMPK/mTOR/ULK signaling pathway, which enhances autophagy. Additionally, reducing NLRP3 levels can also be beneficial. | Huang, Z. et al.([Huang et al., 2021b](#_ENREF_151)) |
|  | MAPKsignaling pathway | Emodin | MCAO, OGD | Activation of the ERK1/2 signaling pathway, increase in Bcl-2 levels, decrease in caspase-3 levels, and reduction in ROS can all contribute to a neuroprotective effect. | Leung, S. W. et al.([Leung et al., 2020](#_ENREF_202)) |
|  |  | Ginsenoside Rg1 | OGD | This therapeutic approach functions by regulating apoptosis, which involves increasing Bcl-2 levels, decreasing Bax levels, and inhibiting Caspase-3 activity. Moreover, it can also reduce oxidative stress and inhibit the phosphorylation of p38 and JNK signaling pathways to achieve its goal of regulating apoptosis. | Li, Y. et al.([Li et al., 2017](#_ENREF_214)) |
|  |  | Baicalin | OGD/R | This therapeutic approach also promotes the release of BDNF, TrKB, PI3K/AKT, and MAPK/ERK, which have anti-oxidative, anti-inflammatory, and neuroprotective effects. | Li, C. et al.([Li et al., 2021a](#_ENREF_206)) |
|  |  | Longxuetongluo | OGD/R | This approach involves inhibiting the MAPK signaling pathway, which can help to inhibit infarction, apoptosis, and endoplasmic reticulum stress. | Pan, B. et al.([Pan et al., 2021](#_ENREF_263)) |
|  |  | Huang-Lian-Jie-Du-Decotion | MCAO/R | The goal of this approach is to protect nerves by regulating the MAPK signaling pathway to induce autophagy, which involves increasing the expression of Beclin-1, ERK, Akt, PDK1, NO, MDA, GSSG, T-SOD, and GSH-px, while decreasing the expression of mTOR, JNK, P38, and PTEN. | Wang, P. R. et al.([Wang et al., 2013](#_ENREF_341)) |
|  |  | Astragaloside VI | MCAO | By targeting the EGF-mediated MAPK signaling pathway, the EGFR/MAPK cascade can be activated, which promotes nerve cell proliferation, neurogenesis, and functional repair. | Chen, X. et al.([Chen et al., 2019b](#_ENREF_55)) |

# Reference

ABDEL-LATIF, R. G., RIFAAI, R. A. & AMIN, E. F. 2020. Empagliflozin alleviates neuronal apoptosis induced by cerebral ischemia/reperfusion injury through HIF-1α/VEGF signaling pathway. *Arch Pharm Res,* 43**,** 514-525.

AGASHE, R. P., LIPPMAN, S. M. & KURZROCK, R. 2022. JAK: Not Just Another Kinase. *Mol Cancer Ther,* 21**,** 1757-1764.

AHMED, S. M., LUO, L., NAMANI, A., WANG, X. J. & TANG, X. 2017. Nrf2 signaling pathway: Pivotal roles in inflammation. *Biochim Biophys Acta Mol Basis Dis,* 1863**,** 585-597.

AKTORIES, K., WELLER, U. & CHHATWAL, G. S. 1987. Clostridium botulinum type C produces a novel ADP-ribosyltransferase distinct from botulinum C2 toxin. *FEBS Lett,* 212**,** 109-13.

ALAM, J., STEWART, D., TOUCHARD, C., BOINAPALLY, S., CHOI, A. M. & COOK, J. L. 1999. Nrf2, a Cap'n'Collar transcription factor, regulates induction of the heme oxygenase-1 gene. *J Biol Chem,* 274**,** 26071-8.

ALBéRI, L., CHI, Z., KADAM, S. D., MULHOLLAND, J. D., DAWSON, V. L., GAIANO, N. & COMI, A. M. 2010. Neonatal stroke in mice causes long-term changes in neuronal Notch-2 expression that may contribute to prolonged injury. *Stroke,* 41**,** S64-71.

ALFIERI, A., SRIVASTAVA, S., SIOW, R. C., MODO, M., FRASER, P. A. & MANN, G. E. 2011. Targeting the Nrf2-Keap1 antioxidant defence pathway for neurovascular protection in stroke. *J Physiol,* 589**,** 4125-36.

ALFIERI, A., SRIVASTAVA, S., SIOW, R. C. M., CASH, D., MODO, M., DUCHEN, M. R., FRASER, P. A., WILLIAMS, S. C. R. & MANN, G. E. 2013. Sulforaphane preconditioning of the Nrf2/HO-1 defense pathway protects the cerebral vasculature against blood-brain barrier disruption and neurological deficits in stroke. *Free Radic Biol Med,* 65**,** 1012-1022.

ALI, C., DOCAGNE, F., NICOLE, O., LESNé, S., TOUTAIN, J., YOUNG, A., CHAZALVIEL, L., DIVOUX, D., CALY, M., CABAL, P., DERLON, J. M., MACKENZIE, E. T., BUISSON, A. & VIVIEN, D. 2001. Increased expression of transforming growth factor-beta after cerebral ischemia in the baboon: an endogenous marker of neuronal stress? *J Cereb Blood Flow Metab,* 21**,** 820-7.

ALIM, I., CAULFIELD, J. T., CHEN, Y., SWARUP, V., GESCHWIND, D. H., IVANOVA, E., SERAVALLI, J., AI, Y., SANSING, L. H., STE MARIE, E. J., HONDAL, R. J., MUKHERJEE, S., CAVE, J. W., SAGDULLAEV, B. T., KARUPPAGOUNDER, S. S. & RATAN, R. R. 2019. Selenium Drives a Transcriptional Adaptive Program to Block Ferroptosis and Treat Stroke. *Cell,* 177**,** 1262-1279.e25.

ANDERSON, K. V., JüRGENS, G. & NüSSLEIN-VOLHARD, C. 1985. Establishment of dorsal-ventral polarity in the Drosophila embryo: genetic studies on the role of the Toll gene product. *Cell,* 42**,** 779-89.

ANDERSSON, E. R. & LENDAHL, U. 2014. Therapeutic modulation of Notch signalling--are we there yet? *Nat Rev Drug Discov,* 13**,** 357-78.

ANDERSSON, E. R., SANDBERG, R. & LENDAHL, U. 2011. Notch signaling: simplicity in design, versatility in function. *Development,* 138**,** 3593-612.

ARTAVANIS-TSAKONAS, S., MUSKAVITCH, M. A. & YEDVOBNICK, B. 1983. Molecular cloning of Notch, a locus affecting neurogenesis in Drosophila melanogaster. *Proc Natl Acad Sci U S A,* 80**,** 1977-81.

ARUMUGAM, T. V., CHAN, S. L., JO, D. G., YILMAZ, G., TANG, S. C., CHENG, A., GLEICHMANN, M., OKUN, E., DIXIT, V. D., CHIGURUPATI, S., MUGHAL, M. R., OUYANG, X., MIELE, L., MAGNUS, T., POOSALA, S., GRANGER, D. N. & MATTSON, M. P. 2006. Gamma secretase-mediated Notch signaling worsens brain damage and functional outcome in ischemic stroke. *Nat Med,* 12**,** 621-3.

ARVIN, B., NEVILLE, L. F., BARONE, F. C. & FEUERSTEIN, G. Z. 1996. The role of inflammation and cytokines in brain injury. *Neurosci Biobehav Rev,* 20**,** 445-52.

ATTISANO, L. & WRANA, J. L. 2002. Signal transduction by the TGF-beta superfamily. *Science,* 296**,** 1646-7.

AUSTIN, J. & KIMBLE, J. 1989. Transcript analysis of glp-1 and lin-12, homologous genes required for cell interactions during development of C. elegans. *Cell,* 58**,** 565-71.

AYLON, Y. & OREN, M. 2007. Living with p53, dying of p53. *Cell,* 130**,** 597-600.

BARONE, F. C. & FEUERSTEIN, G. Z. 1999. Inflammatory mediators and stroke: new opportunities for novel therapeutics. *J Cereb Blood Flow Metab,* 19**,** 819-34.

BARONE, F. C., IRVING, E. A., RAY, A. M., LEE, J. C., KASSIS, S., KUMAR, S., BADGER, A. M., LEGOS, J. J., ERHARDT, J. A., OHLSTEIN, E. H., HUNTER, A. J., HARRISON, D. C., PHILPOTT, K., SMITH, B. R., ADAMS, J. L. & PARSONS, A. A. 2001. Inhibition of p38 mitogen-activated protein kinase provides neuroprotection in cerebral focal ischemia. *Med Res Rev,* 21**,** 129-45.

BARRAT, F. J., CROW, M. K. & IVASHKIV, L. B. 2019. Interferon target-gene expression and epigenomic signatures in health and disease. *Nat Immunol,* 20**,** 1574-1583.

BARTLETT, J. D., CLOSE, G. L., DRUST, B. & MORTON, J. P. 2014. The emerging role of p53 in exercise metabolism. *Sports Med,* 44**,** 303-9.

BERMUDEZ, O., PAGèS, G. & GIMOND, C. 2010. The dual-specificity MAP kinase phosphatases: critical roles in development and cancer. *Am J Physiol Cell Physiol,* 299**,** C189-202.

BHANOT, P., BRINK, M., SAMOS, C. H., HSIEH, J. C., WANG, Y., MACKE, J. P., ANDREW, D., NATHANS, J. & NUSSE, R. 1996. A new member of the frizzled family from Drosophila functions as a Wingless receptor. *Nature,* 382**,** 225-30.

BOULTON, T. G., YANCOPOULOS, G. D., GREGORY, J. S., SLAUGHTER, C., MOOMAW, C., HSU, J. & COBB, M. H. 1990. An insulin-stimulated protein kinase similar to yeast kinases involved in cell cycle control. *Science,* 249**,** 64-7.

BOUTROS, T., CHEVET, E. & METRAKOS, P. 2008. Mitogen-activated protein (MAP) kinase/MAP kinase phosphatase regulation: roles in cell growth, death, and cancer. *Pharmacol Rev,* 60**,** 261-310.

BOYD, J. M., MALSTROM, S., SUBRAMANIAN, T., VENKATESH, L. K., SCHAEPER, U., ELANGOVAN, B., D'SA-EIPPER, C. & CHINNADURAI, G. 1994. Adenovirus E1B 19 kDa and Bcl-2 proteins interact with a common set of cellular proteins. *Cell,* 79**,** 341-51.

BRAND, K., PAGE, S., ROGLER, G., BARTSCH, A., BRANDL, R., KNUECHEL, R., PAGE, M., KALTSCHMIDT, C., BAEUERLE, P. A. & NEUMEIER, D. 1996. Activated transcription factor nuclear factor-kappa B is present in the atherosclerotic lesion. *J Clin Invest,* 97**,** 1715-22.

BREDESEN, D. E. 1995. Neural apoptosis. *Ann Neurol,* 38**,** 839-51.

BRIDGES, C. B. 1916. Non-Disjunction as Proof of the Chromosome Theory of Heredity (Concluded). *Genetics,* 1**,** 107-63.

BURGERING, B. M. & COFFER, P. J. 1995. Protein kinase B (c-Akt) in phosphatidylinositol-3-OH kinase signal transduction. *Nature,* 376**,** 599-602.

BURTON, T. R. & GIBSON, S. B. 2009. The role of Bcl-2 family member BNIP3 in cell death and disease: NIPping at the heels of cell death. *Cell Death Differ,* 16**,** 515-23.

CAMPOS, A. H., WANG, W., POLLMAN, M. J. & GIBBONS, G. H. 2002. Determinants of Notch-3 receptor expression and signaling in vascular smooth muscle cells: implications in cell-cycle regulation. *Circ Res,* 91**,** 999-1006.

CANO, E. & MAHADEVAN, L. C. 1995. Parallel signal processing among mammalian MAPKs. *Trends Biochem Sci,* 20**,** 117-22.

CANTLEY, L. C. 2002. The phosphoinositide 3-kinase pathway. *Science,* 296**,** 1655-7.

CARLING, D., ZAMMIT, V. A. & HARDIE, D. G. 1987. A common bicyclic protein kinase cascade inactivates the regulatory enzymes of fatty acid and cholesterol biosynthesis. *FEBS Lett,* 223**,** 217-22.

CARMELIET, P., DOR, Y., HERBERT, J. M., FUKUMURA, D., BRUSSELMANS, K., DEWERCHIN, M., NEEMAN, M., BONO, F., ABRAMOVITCH, R., MAXWELL, P., KOCH, C. J., RATCLIFFE, P., MOONS, L., JAIN, R. K., COLLEN, D. & KESHERT, E. 1998. Role of HIF-1alpha in hypoxia-mediated apoptosis, cell proliferation and tumour angiogenesis. *Nature,* 394**,** 485-90.

CARRIERE, V., ROUSSEL, L., ORTEGA, N., LACORRE, D. A., AMERICH, L., AGUILAR, L., BOUCHE, G. & GIRARD, J. P. 2007. IL-33, the IL-1-like cytokine ligand for ST2 receptor, is a chromatin-associated nuclear factor in vivo. *Proc Natl Acad Sci U S A,* 104**,** 282-7.

CHAN, J. Y., KWONG, M., LU, R., CHANG, J., WANG, B., YEN, T. S. & KAN, Y. W. 1998. Targeted disruption of the ubiquitous CNC-bZIP transcription factor, Nrf-1, results in anemia and embryonic lethality in mice. *Embo j,* 17**,** 1779-87.

CHAN, P. H. 2005. Mitochondrial dysfunction and oxidative stress as determinants of cell death/survival in stroke. *Ann N Y Acad Sci,* 1042**,** 203-9.

CHANG, H., LIN, C., LI, Z., SHEN, Y., ZHANG, G., MAO, L., MA, C., LIU, N. & LU, H. 2022. T3 alleviates neuroinflammation and reduces early brain injury after subarachnoid haemorrhage by promoting mitophagy via PINK 1-parkin pathway. *Exp Neurol,* 357**,** 114175.

CHANG, T. S. & JENSEN, M. B. 2014. Haemodilution for acute ischaemic stroke. *Cochrane Database Syst Rev,* 2014**,** Cd000103.

CHEN, B., CAO, P., GUO, X., YIN, M., LI, X., JIANG, L., SHAO, J., CHEN, X., JIANG, C., TAO, L., ZHOU, L. & YU, H. 2022a. Maraviroc, an inhibitor of chemokine receptor type 5, alleviates neuroinflammatory response after cerebral Ischemia/reperfusion injury via regulating MAPK/NF-κB signaling. *Int Immunopharmacol,* 108**,** 108755.

CHEN, G., RAY, R., DUBIK, D., SHI, L., CIZEAU, J., BLEACKLEY, R. C., SAXENA, S., GIETZ, R. D. & GREENBERG, A. H. 1997. The E1B 19K/Bcl-2-binding protein Nip3 is a dimeric mitochondrial protein that activates apoptosis. *J Exp Med,* 186**,** 1975-83.

CHEN, J., LIN, X., YAO, C., BINGWA, L. A., WANG, H., LIN, Z., JIN, K., ZHUGE, Q. & YANG, S. 2022b. Transplantation of Roxadustat-preconditioned bone marrow stromal cells improves neurological function recovery through enhancing grafted cell survival in ischemic stroke rats. *CNS Neurosci Ther,* 28**,** 1519-1531.

CHEN, J., ZACHAREK, A., LI, A., CUI, X., ROBERTS, C., LU, M. & CHOPP, M. 2008. Atorvastatin promotes presenilin-1 expression and Notch1 activity and increases neural progenitor cell proliferation after stroke. *Stroke,* 39**,** 220-6.

CHEN, J., ZHANG, X., LIU, X., ZHANG, C., SHANG, W., XUE, J., CHEN, R., XING, Y., SONG, D. & XU, R. 2019a. Ginsenoside Rg1 promotes cerebral angiogenesis via the PI3K/Akt/mTOR signaling pathway in ischemic mice. *Eur J Pharmacol,* 856**,** 172418.

CHEN, L., MIN, J. & WANG, F. 2022c. Copper homeostasis and cuproptosis in health and disease. *Signal Transduct Target Ther,* 7**,** 378.

CHEN, R. L., OGUNSHOLA, O. O., YEOH, K. K., JANI, A., PAPADAKIS, M., NAGEL, S., SCHOFIELD, C. J. & BUCHAN, A. M. 2014. HIF prolyl hydroxylase inhibition prior to transient focal cerebral ischaemia is neuroprotective in mice. *J Neurochem,* 131**,** 177-89.

CHEN, S., PENG, J., SHERCHAN, P., MA, Y., XIANG, S., YAN, F., ZHAO, H., JIANG, Y., WANG, N., ZHANG, J. H. & ZHANG, H. 2020. TREM2 activation attenuates neuroinflammation and neuronal apoptosis via PI3K/Akt pathway after intracerebral hemorrhage in mice. *J Neuroinflammation,* 17**,** 168.

CHEN, S., ZHANG, J., LI, M., ZHOU, J. & ZHANG, Y. 2022d. Danhong injection combined with tPA protects the BBB through Notch-VEGF signaling pathway on long-term outcomes of thrombolytic therapy. *Biomed Pharmacother,* 153**,** 113288.

CHEN, X., KANG, R., KROEMER, G. & TANG, D. 2021a. Broadening horizons: the role of ferroptosis in cancer. *Nat Rev Clin Oncol,* 18**,** 280-296.

CHEN, X., KANG, R., KROEMER, G. & TANG, D. 2021b. Ferroptosis in infection, inflammation, and immunity. *J Exp Med,* 218.

CHEN, X., WU, H., CHEN, H., WANG, Q., XIE, X. J. & SHEN, J. 2019b. Astragaloside VI Promotes Neural Stem Cell Proliferation and Enhances Neurological Function Recovery in Transient Cerebral Ischemic Injury via Activating EGFR/MAPK Signaling Cascades. *Mol Neurobiol,* 56**,** 3053-3067.

CHENG, Y. L., PARK, J. S., MANZANERO, S., CHOI, Y., BAIK, S. H., OKUN, E., GELDERBLOM, M., FANN, D. Y., MAGNUS, T., LAUNIKONIS, B. S., MATTSON, M. P., SOBEY, C. G., JO, D. G. & ARUMUGAM, T. V. 2014. Evidence that collaboration between HIF-1α and Notch-1 promotes neuronal cell death in ischemic stroke. *Neurobiol Dis,* 62**,** 286-95.

CHIEN, M. Y., CHUANG, C. H., CHERN, C. M., LIOU, K. T., LIU, D. Z., HOU, Y. C. & SHEN, Y. C. 2016. Salvianolic acid A alleviates ischemic brain injury through the inhibition of inflammation and apoptosis and the promotion of neurogenesis in mice. *Free Radic Biol Med,* 99**,** 508-519.

CHINNADURAI, G., VIJAYALINGAM, S. & GIBSON, S. B. 2008. BNIP3 subfamily BH3-only proteins: mitochondrial stress sensors in normal and pathological functions. *Oncogene,* 27 Suppl 1**,** S114-27.

CHIO, I. I. C., JAFARNEJAD, S. M., PONZ-SARVISE, M., PARK, Y., RIVERA, K., PALM, W., WILSON, J., SANGAR, V., HAO, Y., ÖHLUND, D., WRIGHT, K., FILIPPINI, D., LEE, E. J., DA SILVA, B., SCHOEPFER, C., WILKINSON, J. E., BUSCAGLIA, J. M., DENICOLA, G. M., TIRIAC, H., HAMMELL, M., CRAWFORD, H. C., SCHMIDT, E. E., THOMPSON, C. B., PAPPIN, D. J., SONENBERG, N. & TUVESON, D. A. 2016. NRF2 Promotes Tumor Maintenance by Modulating mRNA Translation in Pancreatic Cancer. *Cell,* 166**,** 963-976.

CHOU, W. C., RAMPANELLI, E., LI, X. & TING, J. P. 2022. Impact of intracellular innate immune receptors on immunometabolism. *Cell Mol Immunol,* 19**,** 337-351.

CINELLI, M. A., DO, H. T., MILEY, G. P. & SILVERMAN, R. B. 2020. Inducible nitric oxide synthase: Regulation, structure, and inhibition. *Med Res Rev,* 40**,** 158-189.

CLARK, I. E., DODSON, M. W., JIANG, C., CAO, J. H., HUH, J. R., SEOL, J. H., YOO, S. J., HAY, B. A. & GUO, M. 2006. Drosophila pink1 is required for mitochondrial function and interacts genetically with parkin. *Nature,* 441**,** 1162-6.

COCHRANE, A., CHEN, C., STEPHEN, J., RøNNING, O. M., ANDERSON, C. S., HANKEY, G. J. & AL-SHAHI SALMAN, R. 2023. Antithrombotic treatment after stroke due to intracerebral haemorrhage. *Cochrane Database Syst Rev,* 1**,** Cd012144.

COFFMAN, C., HARRIS, W. & KINTNER, C. 1990. Xotch, the Xenopus homolog of Drosophila notch. *Science,* 249**,** 1438-41.

COOK, D. N., PISETSKY, D. S. & SCHWARTZ, D. A. 2004. Toll-like receptors in the pathogenesis of human disease. *Nat Immunol,* 5**,** 975-9.

COOPER, J. A., BOWEN-POPE, D. F., RAINES, E., ROSS, R. & HUNTER, T. 1982. Similar effects of platelet-derived growth factor and epidermal growth factor on the phosphorylation of tyrosine in cellular proteins. *Cell,* 31**,** 263-73.

COOPERATIVE GROUP FOR REASSESSMENT OF, D. 2005. Reassessment of defibrase in treatment of acute cerebral infarction: a multicenter, randomized, double-blind, placebo-controlled trial. *Chin Med Sci J,* 20**,** 151-8.

COUZIN-FRANKEL, J. 2013. Breakthrough of the year 2013. Cancer immunotherapy. *Science,* 342**,** 1432-3.

COYLE, J. T. & PUTTFARCKEN, P. 1993. Oxidative stress, glutamate, and neurodegenerative disorders. *Science,* 262**,** 689-95.

CROLL, S. D. & WIEGAND, S. J. 2001. Vascular growth factors in cerebral ischemia. *Mol Neurobiol,* 23**,** 121-35.

CUI, H. Y., ZHANG, X. J., YANG, Y., ZHANG, C., ZHU, C. H., MIAO, J. Y. & CHEN, R. 2018. Rosmarinic acid elicits neuroprotection in ischemic stroke via Nrf2 and heme oxygenase 1 signaling. *Neural Regen Res,* 13**,** 2119-2128.

CUI, Y., ZHANG, Y., ZHAO, X., SHAO, L., LIU, G., SUN, C., XU, R. & ZHANG, Z. 2021. ACSL4 exacerbates ischemic stroke by promoting ferroptosis-induced brain injury and neuroinflammation. *Brain Behav Immun,* 93**,** 312-321.

CULMAN, J., ZHAO, Y., GOHLKE, P. & HERDEGEN, T. 2007. PPAR-gamma: therapeutic target for ischemic stroke. *Trends Pharmacol Sci,* 28**,** 244-9.

DARNELL, J. E., JR., KERR, I. M. & STARK, G. R. 1994. Jak-STAT pathways and transcriptional activation in response to IFNs and other extracellular signaling proteins. *Science,* 264**,** 1415-21.

DáVALOS, A. & SECADES, J. 2011. Citicoline preclinical and clinical update 2009-2010. *Stroke,* 42**,**S36-9.

DAVE, K. D., DE SILVA, S., SHETH, N. P., RAMBOZ, S., BECK, M. J., QUANG, C., SWITZER, R. C., 3RD, AHMAD, S. O., SUNKIN, S. M., WALKER, D., CUI, X., FISHER, D. A., MCCOY, A. M., GAMBER, K., DING, X., GOLDBERG, M. S., BENKOVIC, S. A., HAUPT, M., BAPTISTA, M. A., FISKE, B. K., SHERER, T. B. & FRASIER, M. A. 2014. Phenotypic characterization of recessive gene knockout rat models of Parkinson's disease. *Neurobiol Dis,* 70**,** 190-203.

DAVIS, R. J. 1994. MAPKs: new JNK expands the group. *Trends Biochem Sci,* 19**,** 470-3.

DELEO, A. B., JAY, G., APPELLA, E., DUBOIS, G. C., LAW, L. W. & OLD, L. J. 1979. Detection of a transformation-related antigen in chemically induced sarcomas and other transformed cells of the mouse. *Proc Natl Acad Sci U S A,* 76**,** 2420-4.

DIDONATO, J. A., MERCURIO, F. & KARIN, M. 2012. NF-κB and the link between inflammation and cancer. *Immunol Rev,* 246**,** 379-400.

DING, J. Y., PAN, L. Q., HU, Y. Y., RAJAH, G. B., ZHOU, D., BAI, C. B., YA, J. Y., WANG, Z. A., JIN, K. X., GUAN, J. W., DING, Y. C., JI, X. M. & MENG, R. 2019a. Batroxobin in combination with anticoagulation may promote venous sinus recanalization in cerebral venous thrombosis: A real-world experience. *CNS Neurosci Ther,* 25**,** 638-646.

DING, Y., CHEN, M., WANG, M., LI, Y. & WEN, A. 2015. Posttreatment with 11-Keto-β-Boswellic Acid Ameliorates Cerebral Ischemia-Reperfusion Injury: Nrf2/HO-1 Pathway as a Potential Mechanism. *Mol Neurobiol,* 52**,** 1430-1439.

DING, Y., QIAN, J., LI, H., SHEN, H., LI, X., KONG, Y., XU, Z. & CHEN, G. 2019b. Effects of SC99 on cerebral ischemia-perfusion injury in rats: Selective modulation of microglia polarization to M2 phenotype via inhibiting JAK2-STAT3 pathway. *Neurosci Res,* 142**,** 58-68.

DIXON, S. J., LEMBERG, K. M., LAMPRECHT, M. R., SKOUTA, R., ZAITSEV, E. M., GLEASON, C. E., PATEL, D. N., BAUER, A. J., CANTLEY, A. M., YANG, W. S., MORRISON, B., 3RD & STOCKWELL, B. R. 2012. Ferroptosis: an iron-dependent form of nonapoptotic cell death. *Cell,* 149**,** 1060-72.

DOLL, S., PRONETH, B., TYURINA, Y. Y., PANZILIUS, E., KOBAYASHI, S., INGOLD, I., IRMLER, M., BECKERS, J., AICHLER, M., WALCH, A., PROKISCH, H., TRüMBACH, D., MAO, G., QU, F., BAYIR, H., FüLLEKRUG, J., SCHEEL, C. H., WURST, W., SCHICK, J. A., KAGAN, V. E., ANGELI, J. P. & CONRAD, M. 2017. ACSL4 dictates ferroptosis sensitivity by shaping cellular lipid composition. *Nat Chem Biol,* 13**,** 91-98.

DONG, W., XIAN, Y., YUAN, W., HUIFENG, Z., TAO, W., ZHIQIANG, L., SHAN, F., YA, F., HONGLI, W., JINGHUAN, W., LEI, Q., LI, Z. & HONGYI, Q. 2016. Catalpol stimulates VEGF production via the JAK2/STAT3 pathway to improve angiogenesis in rats' stroke model. *J Ethnopharmacol,* 191**,** 169-179.

DUAN, C., WANG, H., JIAO, D., GENG, Y., WU, Q., YAN, H. & LI, C. 2022. Curcumin Restrains Oxidative Stress of After Intracerebral Hemorrhage in Rat by Activating the Nrf2/HO-1 Pathway. *Front Pharmacol,* 13**,** 889226.

DUAN, J., CUI, J., YANG, Z., GUO, C., CAO, J., XI, M., WENG, Y., YIN, Y., WANG, Y., WEI, G., QIAO, B. & WEN, A. 2019. Neuroprotective effect of Apelin 13 on ischemic stroke by activating AMPK/GSK-3β/Nrf2 signaling. *J Neuroinflammation,* 16**,** 24.

DUAN, L., ZHANG, Y., YANG, Y., SU, S., ZHOU, L., LO, P. C., CAI, J., QIAO, Y., LI, M., HUANG, S., WANG, H., MO, Y. & WANG, Q. 2021. Baicalin Inhibits Ferroptosis in Intracerebral Hemorrhage. *Front Pharmacol,* 12**,** 629379.

DURRANT, T. N. & HERS, I. 2020. PI3K inhibitors in thrombosis and cardiovascular disease. *Clin Transl Med,* 9**,** 8.

ECHELARD, Y., EPSTEIN, D. J., ST-JACQUES, B., SHEN, L., MOHLER, J., MCMAHON, J. A. & MCMAHON, A. P. 1993. Sonic hedgehog, a member of a family of putative signaling molecules, is implicated in the regulation of CNS polarity. *Cell,* 75**,** 1417-30.

ENDRES, M., LAUFS, U., LIAO, J. K. & MOSKOWITZ, M. A. 2004. Targeting eNOS for stroke protection. *Trends Neurosci,* 27**,** 283-9.

ERLICH, S., SHOHAMI, E. & PINKAS-KRAMARSKI, R. 2000. Closed head injury induces up-regulation of ErbB-4 receptor at the site of injury. *Mol Cell Neurosci,* 16**,** 597-608.

FANN, D. Y., LIM, Y. A., CHENG, Y. L., LOK, K. Z., CHUNDURI, P., BAIK, S. H., DRUMMOND, G. R., DHEEN, S. T., SOBEY, C. G., JO, D. G., CHEN, C. L. & ARUMUGAM, T. V. 2018. Evidence that NF-κB and MAPK Signaling Promotes NLRP Inflammasome Activation in Neurons Following Ischemic Stroke. *Mol Neurobiol,* 55**,** 1082-1096.

FEDOROWICZ, M. A., DE VRIES-SCHNEIDER, R. L., RüB, C., BECKER, D., HUANG, Y., ZHOU, C., ALESSI WOLKEN, D. M., VOOS, W., LIU, Y. & PRZEDBORSKI, S. 2014. Cytosolic cleaved PINK1 represses Parkin translocation to mitochondria and mitophagy. *EMBO Rep,* 15**,** 86-93.

FENG, S., YANG, Q., LIU, M., LI, W., YUAN, W., ZHANG, S., WU, B. & LI, J. 2011. Edaravone for acute ischaemic stroke. *Cochrane Database Syst Rev***,** Cd007230.

FERRARA, N. 2001. Role of vascular endothelial growth factor in regulation of physiological angiogenesis. *Am J Physiol Cell Physiol,* 280**,** C1358-66.

FERRARA, N. & HENZEL, W. J. 1989. Pituitary follicular cells secrete a novel heparin-binding growth factor specific for vascular endothelial cells. *Biochem Biophys Res Commun,* 161**,** 851-8.

FONG, W. H., TSAI, H. D., CHEN, Y. C., WU, J. S. & LIN, T. N. 2010. Anti-apoptotic actions of PPAR-gamma against ischemic stroke. *Mol Neurobiol,* 41**,** 180-6.

FOSTER-GOLDMAN, A. & MCCARTHY, D. 2013. Angioedema from recombinant TPA administration: case report and pathophysiology review. *Am J Ther,* 20**,** 691-3.

FRAME, S. & COHEN, P. 2001. GSK3 takes centre stage more than 20 years after its discovery. *Biochem J,* 359**,** 1-16.

FRANKE, T. F., YANG, S. I., CHAN, T. O., DATTA, K., KAZLAUSKAS, A., MORRISON, D. K., KAPLAN, D. R. & TSICHLIS, P. N. 1995. The protein kinase encoded by the Akt proto-oncogene is a target of the PDGF-activated phosphatidylinositol 3-kinase. *Cell,* 81**,** 727-36.

FRESNO VARA, J. A., CASADO, E., DE CASTRO, J., CEJAS, P., BELDA-INIESTA, C. & GONZáLEZ-BARóN, M. 2004. PI3K/Akt signalling pathway and cancer. *Cancer Treat Rev,* 30**,** 193-204.

FU, A., EBERHARD, C. E. & SCREATON, R. A. 2013. Role of AMPK in pancreatic beta cell function. *Mol Cell Endocrinol,* 366**,** 127-34.

FU, C., WU, Y., LIU, S., LUO, C., LU, Y., LIU, M., WANG, L., ZHANG, Y. & LIU, X. 2022. Rehmannioside A improves cognitive impairment and alleviates ferroptosis via activating PI3K/AKT/Nrf2 and SLC7A11/GPX4 signaling pathway after ischemia. *J Ethnopharmacol,* 289**,** 115021.

FURCHGOTT, R. F. & ZAWADZKI, J. V. 1980. The obligatory role of endothelial cells in the relaxation of arterial smooth muscle by acetylcholine. *Nature,* 288**,** 373-6.

FURLAN, A., HIGASHIDA, R., WECHSLER, L., GENT, M., ROWLEY, H., KASE, C., PESSIN, M., AHUJA, A., CALLAHAN, F., CLARK, W. M., SILVER, F. & RIVERA, F. 1999. Intra-arterial prourokinase for acute ischemic stroke. The PROACT II study: a randomized controlled trial. Prolyse in Acute Cerebral Thromboembolism. *Jama,* 282**,** 2003-11.

GEISLER, S., HOLMSTRöM, K. M., SKUJAT, D., FIESEL, F. C., ROTHFUSS, O. C., KAHLE, P. J. & SPRINGER, W. 2010. PINK1/Parkin-mediated mitophagy is dependent on VDAC1 and p62/SQSTM1. *Nat Cell Biol,* 12**,** 119-31.

GHIGO, A., LAFFARGUE, M., LI, M. & HIRSCH, E. 2017. PI3K and Calcium Signaling in Cardiovascular Disease. *Circ Res,* 121**,** 282-292.

GOLD, R., KAPPOS, L., ARNOLD, D. L., BAR-OR, A., GIOVANNONI, G., SELMAJ, K., TORNATORE, C., SWEETSER, M. T., YANG, M., SHEIKH, S. I. & DAWSON, K. T. 2012. Placebo-controlled phase 3 study of oral BG-12 for relapsing multiple sclerosis. *N Engl J Med,* 367**,** 1098-107.

GONG, S., MA, H., ZHENG, F., HUANG, J., ZHANG, Y., YU, B., LI, F. & KOU, J. 2021. Inhibiting YAP in Endothelial Cells From Entering the Nucleus Attenuates Blood-Brain Barrier Damage During Ischemia-Reperfusion Injury. *Front Pharmacol,* 12**,** 777680.

GOUGH, N. R., XIANG, X. & MISHRA, L. 2021. TGF-β Signaling in Liver, Pancreas, and Gastrointestinal Diseases and Cancer. *Gastroenterology,* 161**,** 434-452.e15.

GRACEY, E., HROMADOVá, D., LIM, M., QAIYUM, Z., ZENG, M., YAO, Y., SRINATH, A., BAGLAENKO, Y., YEREMENKO, N., WESTLIN, W., MASSE, C., MüLLER, M., STROBL, B., MIAO, W. & INMAN, R. D. 2020. TYK2 inhibition reduces type 3 immunity and modifies disease progression in murine spondyloarthritis. *J Clin Invest,* 130**,** 1863-1878.

GRAHAME HARDIE, D. 2016. Regulation of AMP-activated protein kinase by natural and synthetic activators. *Acta Pharm Sin B,* 6**,** 1-19.

GREENLUND, A. C., FARRAR, M. A., VIVIANO, B. L. & SCHREIBER, R. D. 1994. Ligand-induced IFN gamma receptor tyrosine phosphorylation couples the receptor to its signal transduction system (p91). *Embo j,* 13**,** 1591-600.

GREER, D. M. 2010. Aspirin and antiplatelet agent resistance: implications for prevention of secondary stroke. *CNS Drugs,* 24**,** 1027-40.

GRETEN, F. R., ECKMANN, L., GRETEN, T. F., PARK, J. M., LI, Z. W., EGAN, L. J., KAGNOFF, M. F. & KARIN, M. 2004. IKKbeta links inflammation and tumorigenesis in a mouse model of colitis-associated cancer. *Cell,* 118**,** 285-96.

GRIFFITH, T. M., EDWARDS, D. H., LEWIS, M. J., NEWBY, A. C. & HENDERSON, A. H. 1984. The nature of endothelium-derived vascular relaxant factor. *Nature,* 308**,** 645-7.

GROSS, S. S. & WOLIN, M. S. 1995. Nitric oxide: pathophysiological mechanisms. *Annu Rev Physiol,* 57**,** 737-69.

GUO, H., ZHANG, W., WANG, Z., LI, Z., ZHOU, J. & YANG, Z. 2022. Dexmedetomidine post-conditioning protects blood-brain barrier integrity by modulating microglia/macrophage polarization via inhibiting NF-κB signaling pathway in intracerebral hemorrhage. *Front Mol Neurosci,* 15**,** 977941.

HANSON, L. R., ROEYTENBERG, A., MARTINEZ, P. M., COPPES, V. G., SWEET, D. C., RAO, R. J., MARTI, D. L., HOEKMAN, J. D., MATTHEWS, R. B., FREY, W. H., 2ND & PANTER, S. S. 2009. Intranasal deferoxamine provides increased brain exposure and significant protection in rat ischemic stroke. *J Pharmacol Exp Ther,* 330**,** 679-86.

HANSSON, E. M., LENDAHL, U. & CHAPMAN, G. 2004. Notch signaling in development and disease. *Semin Cancer Biol,* 14**,** 320-8.

HARRIS, C. C. 1993. p53: at the crossroads of molecular carcinogenesis and risk assessment. *Science,* 262**,** 1980-1.

HARVEY, K. F., PFLEGER, C. M. & HARIHARAN, I. K. 2003. The Drosophila Mst ortholog, hippo, restricts growth and cell proliferation and promotes apoptosis. *Cell,* 114**,** 457-67.

HASHIMOTO, C., HUDSON, K. L. & ANDERSON, K. V. 1988. The Toll gene of Drosophila, required for dorsal-ventral embryonic polarity, appears to encode a transmembrane protein. *Cell,* 52**,** 269-79.

HAYDEN, M. S. & GHOSH, S. 2008. Shared principles in NF-kappaB signaling. *Cell,* 132**,** 344-62.

HAYES, J. D., MCMAHON, M., CHOWDHRY, S. & DINKOVA-KOSTOVA, A. T. 2010. Cancer chemoprevention mechanisms mediated through the Keap1-Nrf2 pathway. *Antioxid Redox Signal,* 13**,** 1713-48.

HE, Q., MA, Y., LIU, J., ZHANG, D., REN, J., ZHAO, R., CHANG, J., GUO, Z. N. & YANG, Y. 2021. Biological Functions and Regulatory Mechanisms of Hypoxia-Inducible Factor-1α in Ischemic Stroke. *Front Immunol,* 12**,** 801985.

HE, Q. W., XIA, Y. P., CHEN, S. C., WANG, Y., HUANG, M., HUANG, Y., LI, J. Y., LI, Y. N., GAO, Y., MAO, L., MEI, Y. W. & HU, B. 2013. Astrocyte-derived sonic hedgehog contributes to angiogenesis in brain microvascular endothelial cells via RhoA/ROCK pathway after oxygen-glucose deprivation. *Mol Neurobiol,* 47**,** 976-87.

HELTON, R., CUI, J., SCHEEL, J. R., ELLISON, J. A., AMES, C., GIBSON, C., BLOUW, B., OUYANG, L., DRAGATSIS, I., ZEITLIN, S., JOHNSON, R. S., LIPTON, S. A. & BARLOW, C. 2005. Brain-specific knock-out of hypoxia-inducible factor-1alpha reduces rather than increases hypoxic-ischemic damage. *J Neurosci,* 25**,** 4099-107.

HENKE, N., ALBRECHT, P., BOUCHACHIA, I., RYAZANTSEVA, M., KNOLL, K., LEWERENZ, J., KAZNACHEYEVA, E., MAHER, P. & METHNER, A. 2013. The plasma membrane channel ORAI1 mediates detrimental calcium influx caused by endogenous oxidative stress. *Cell Death Dis,* 4**,** e470.

HERR, I. & DEBATIN, K. M. 2001. Cellular stress response and apoptosis in cancer therapy. *Blood,* 98**,** 2603-14.

HERZIG, S. & SHAW, R. J. 2018. AMPK: guardian of metabolism and mitochondrial homeostasis. *Nat Rev Mol Cell Biol,* 19**,** 121-135.

HIGASHIDA, R. T., TSAI, F. Y., HALBACH, V. V., BARNWELL, S. L., DOWD, C. F. & HIESHIMA, G. B. 1995. Interventional neurovascular techniques in the treatment of stroke--state-of-the-art therapy. *J Intern Med,* 237**,** 105-15.

HIROOKA, Y. & SHIMOKAWA, H. 2005. Therapeutic potential of rho-kinase inhibitors in cardiovascular diseases. *Am J Cardiovasc Drugs,* 5**,** 31-9.

HIRSCHHORN, T. & STOCKWELL, B. R. 2019. The development of the concept of ferroptosis. *Free Radical Biology and Medicine,* 133**,** 130-143.

HLAVICA, M., DIEPERS, M., GARCIA-ESPERON, C., INEICHEN, B. V., NEDELTCHEV, K., KAHLES, T. & REMONDA, L. 2015. Pharmacological recanalization therapy in acute ischemic stroke - evolution, current state and perspectives of intravenous and intra-arterial thrombolysis. *J Neuroradiol,* 42**,** 30-46.

HOLLSTEIN, M., SIDRANSKY, D., VOGELSTEIN, B. & HARRIS, C. C. 1991. p53 mutations in human cancers. *Science,* 253**,** 49-53.

HOMMA, T., KOBAYASHI, S., SATO, H. & FUJII, J. 2019. Edaravone, a free radical scavenger, protects against ferroptotic cell death in vitro. *Exp Cell Res,* 384**,** 111592.

HONG, L., CHEN, W., HE, L., TAN, H., PENG, D., ZHAO, G., SHI, X., WANG, L., LIU, M. & JIANG, H. 2021. Effect of Naoluoxintong on the NogoA/RhoA/ROCK pathway by down-regulating DNA methylation in MCAO rats. *J Ethnopharmacol,* 281**,** 114559.

HOU, Y., WANG, K., WAN, W., CHENG, Y., PU, X. & YE, X. 2018. Resveratrol provides neuroprotection by regulating the JAK2/STAT3/PI3K/AKT/mTOR pathway after stroke in rats. *Genes Dis,* 5**,** 245-255.

HSU, C. C., PENG, D., CAI, Z. & LIN, H. K. 2022. AMPK signaling and its targeting in cancer progression and treatment. *Semin Cancer Biol,* 85**,** 52-68.

HU, G. Q., DU, X., LI, Y. J., GAO, X. Q., CHEN, B. Q. & YU, L. 2017. Inhibition of cerebral ischemia/reperfusion injury-induced apoptosis: nicotiflorin and JAK2/STAT3 pathway. *Neural Regen Res,* 12**,** 96-102.

HU, Q., LIU, L., ZHOU, L., LU, H., WANG, J., CHEN, X. & WANG, Q. 2020. Effect of fluoxetine on HIF-1α- Netrin/VEGF cascade, angiogenesis and neuroprotection in a rat model of transient middle cerebral artery occlusion. *Exp Neurol,* 329**,** 113312.

HU, Q., ZUO, T., DENG, L., CHEN, S., YU, W., LIU, S., LIU, J., WANG, X., FAN, X. & DONG, Z. 2022a. β-Caryophyllene suppresses ferroptosis induced by cerebral ischemia reperfusion via activation of the NRF2/HO-1 signaling pathway in MCAO/R rats. *Phytomedicine,* 102**,** 154112.

HU, S., WU, Y., ZHAO, B., HU, H., ZHU, B., SUN, Z., LI, P. & DU, S. 2018. Panax notoginseng Saponins Protect Cerebral Microvascular Endothelial Cells against Oxygen-Glucose Deprivation/Reperfusion-Induced Barrier Dysfunction via Activation of PI3K/Akt/Nrf2 Antioxidant Signaling Pathway. *Molecules,* 23.

HU, Y., ZHENG, Y., WANG, T., JIAO, L. & LUO, Y. 2022b. VEGF, a Key Factor for Blood Brain Barrier Injury After Cerebral Ischemic Stroke. *Aging Dis,* 13**,** 647-654.

HUANG, J., WU, S., BARRERA, J., MATTHEWS, K. & PAN, D. 2005. The Hippo signaling pathway coordinately regulates cell proliferation and apoptosis by inactivating Yorkie, the Drosophila Homolog of YAP. *Cell,* 122**,** 421-34.

HUANG, L., LI, S., DAI, Q., ZHANG, A., YU, Q., DU, W., ZHAO, P., MO, Y., XU, K., CHEN, S. & WANG, J. 2020. Astrocytic Yes-associated protein attenuates cerebral ischemia-induced brain injury by regulating signal transducer and activator of transcription 3 signaling. *Exp Neurol,*333**,** 113431.

HUANG, S. S., CHENG, H., TANG, C. M., NIEN, M. W., HUANG, Y. S., LEE, I. H., YIN, J. H., KUO, T. B., YANG, C. C., TSAI, S. K. & YANG, D. I. 2013. Anti-oxidative, anti-apoptotic, and pro-angiogenic effects mediate functional improvement by sonic hedgehog against focal cerebral ischemia in rats. *Exp Neurol,* 247**,** 680-8.

HUANG, Z., GUO, L., HUANG, L., SHI, Y., LIANG, J. & ZHAO, L. 2021a. Baicalin-loaded macrophage-derived exosomes ameliorate ischemic brain injury via the antioxidative pathway. *Mater Sci Eng C Mater Biol Appl,* 126**,** 112123.

HUANG, Z., ZHOU, X., ZHANG, X., HUANG, L., SUN, Y., CHENG, Z., XU, W., LI, C. G., ZHENG, Y. & HUANG, M. 2021b. Pien-Tze-Huang, a Chinese patent formula, attenuates NLRP3 inflammasome-related neuroinflammation by enhancing autophagy via the AMPK/mTOR/ULK1 signaling pathway. *Biomed Pharmacother,* 141**,** 111814.

HUBER, O., KORN, R., MCLAUGHLIN, J., OHSUGI, M., HERRMANN, B. G. & KEMLER, R. 1996. Nuclear localization of beta-catenin by interaction with transcription factor LEF-1. *Mech Dev,* 59**,** 3-10.

IGNARRO, L. J., BYRNS, R. E., BUGA, G. M. & WOOD, K. S. 1987. Endothelium-derived relaxing factor from pulmonary artery and vein possesses pharmacologic and chemical properties identical to those of nitric oxide radical. *Circ Res,* 61**,** 866-79.

INFANTE, P., ALFONSI, R., BOTTA, B., MORI, M. & DI MARCOTULLIO, L. 2015. Targeting GLI factors to inhibit the Hedgehog pathway. *Trends Pharmacol Sci,* 36**,** 547-58.

IRVING, E. A., BARONE, F. C., REITH, A. D., HADINGHAM, S. J. & PARSONS, A. A. 2000. Differential activation of MAPK/ERK and p38/SAPK in neurones and glia following focal cerebral ischaemia in the rat. *Brain Res Mol Brain Res,* 77**,** 65-75.

ISAACS, A. & BURKE, D. C. 1958. Mode of action of interferon. *Nature,* 182**,** 1073-4.

ISHIKAWA, H., TAJIRI, N., SHINOZUKA, K., VASCONCELLOS, J., KANEKO, Y., LEE, H. J., MIMURA, O., DEZAWA, M., KIM, S. U. & BORLONGAN, C. V. 2013. Vasculogenesis in experimental stroke after human cerebral endothelial cell transplantation. *Stroke,* 44**,** 3473-81.

ISSEMANN, I. & GREEN, S. 1990. Activation of a member of the steroid hormone receptor superfamily by peroxisome proliferators. *Nature,* 347**,** 645-50.

ITO, D., TANAHASHI, N., MURATA, M., SATO, H., SAITO, I., WATANABE, K. & FUKUUCHI, Y. 2002. Notch3 gene polymorphism and ischaemic cerebrovascular disease. *J Neurol Neurosurg Psychiatry,* 72**,** 382-4.

ITOH, K., WAKABAYASHI, N., KATOH, Y., ISHII, T., IGARASHI, K., ENGEL, J. D. & YAMAMOTO, M. 1999. Keap1 represses nuclear activation of antioxidant responsive elements by Nrf2 through binding to the amino-terminal Neh2 domain. *Genes Dev,* 13**,** 76-86.

IVAN, M., KONDO, K., YANG, H., KIM, W., VALIANDO, J., OHH, M., SALIC, A., ASARA, J. M., LANE, W. S. & KAELIN, W. G., JR. 2001. HIFalpha targeted for VHL-mediated destruction by proline hydroxylation: implications for O2 sensing. *Science,* 292**,** 464-8.

JAAKKOLA, P., MOLE, D. R., TIAN, Y. M., WILSON, M. I., GIELBERT, J., GASKELL, S. J., VON KRIEGSHEIM, A., HEBESTREIT, H. F., MUKHERJI, M., SCHOFIELD, C. J., MAXWELL, P. H., PUGH, C. W. & RATCLIFFE, P. J. 2001. Targeting of HIF-alpha to the von Hippel-Lindau ubiquitylation complex by O2-regulated prolyl hydroxylation. *Science,*292**,** 468-72.

JIA, J., CHENG, J., NI, J. & ZHEN, X. 2015. Neuropharmacological Actions of Metformin in Stroke. *Curr Neuropharmacol,* 13**,** 389-94.

JIANG, M., LIU, X., ZHANG, D., WANG, Y., HU, X., XU, F., JIN, M., CAO, F. & XU, L. 2018a. Celastrol treatment protects against acute ischemic stroke-induced brain injury by promoting an IL-33/ST2 axis-mediated microglia/macrophage M2 polarization. *J Neuroinflammation,* 15**,** 78.

JIANG, S., LI, T., JI, T., YI, W., YANG, Z., WANG, S., YANG, Y. & GU, C. 2018b. AMPK: Potential Therapeutic Target for Ischemic Stroke. *Theranostics,* 8**,** 4535-4551.

JIANG, T., YU, J. T., ZHU, X. C., ZHANG, Q. Q., TAN, M. S., CAO, L., WANG, H. F., SHI, J. Q., GAO, L., QIN, H., ZHANG, Y. D. & TAN, L. 2015. Ischemic preconditioning provides neuroprotection by induction of AMP-activated protein kinase-dependent autophagy in a rat model of ischemic stroke. *Mol Neurobiol,* 51**,** 220-9.

JOVANOVIC, I. P., PEJNOVIC, N. N., RADOSAVLJEVIC, G. D., ARSENIJEVIC, N. N. & LUKIC, M. L. 2012. IL-33/ST2 axis in innate and acquired immunity to tumors. *Oncoimmunology,* 1**,** 229-231.

JUSTICE, R. W., ZILIAN, O., WOODS, D. F., NOLL, M. & BRYANT, P. J. 1995. The Drosophila tumor suppressor gene warts encodes a homolog of human myotonic dystrophy kinase and is required for the control of cell shape and proliferation. *Genes Dev,* 9**,** 534-46.

JUSTICIA, C., GABRIEL, C. & PLANAS, A. M. 2000. Activation of the JAK/STAT pathway following transient focal cerebral ischemia: signaling through Jak1 and Stat3 in astrocytes. *Glia,* 30**,** 253-70.

KADER, A., FRAZZINI, V. I., SOLOMON, R. A. & TRIFILETTI, R. R. 1993. Nitric oxide production during focal cerebral ischemia in rats. *Stroke,* 24**,** 1709-16.

KANG, J. S., LIU, C. & DERYNCK, R. 2009. New regulatory mechanisms of TGF-beta receptor function. *Trends Cell Biol,* 19**,** 385-94.

KANGO-SINGH, M., NOLO, R., TAO, C., VERSTREKEN, P., HIESINGER, P. R., BELLEN, H. J. & HALDER, G. 2002. Shar-pei mediates cell proliferation arrest during imaginal disc growth in Drosophila. *Development,* 129**,** 5719-30.

KARAMAN, S., LEPPäNEN, V. M. & ALITALO, K. 2018. Vascular endothelial growth factor signaling in development and disease. *Development,* 145.

KARIKó, K., WEISSMAN, D. & WELSH, F. A. 2004. Inhibition of toll-like receptor and cytokine signaling--a unifying theme in ischemic tolerance. *J Cereb Blood Flow Metab,* 24**,** 1288-304.

KAWAI, T. & AKIRA, S. 2007. Signaling to NF-kappaB by Toll-like receptors. *Trends Mol Med,* 13**,** 460-9.

KEMP, B. E., MITCHELHILL, K. I., STAPLETON, D., MICHELL, B. J., CHEN, Z. P. & WITTERS, L. A. 1999. Dealing with energy demand: the AMP-activated protein kinase. *Trends Biochem Sci,* 24**,** 22-5.

KHWAJA, A., RODRIGUEZ-VICIANA, P., WENNSTRöM, S., WARNE, P. H. & DOWNWARD, J. 1997. Matrix adhesion and Ras transformation both activate a phosphoinositide 3-OH kinase and protein kinase B/Akt cellular survival pathway. *Embo j,* 16**,** 2783-93.

KIDD, S., KELLEY, M. R. & YOUNG, M. W. 1986. Sequence of the notch locus of Drosophila melanogaster: relationship of the encoded protein to mammalian clotting and growth factors. *Mol Cell Biol,* 6**,** 3094-108.

KIM, H. J., HAWKE, N. & BALDWIN, A. S. 2006. NF-kappaB and IKK as therapeutic targets in cancer. *Cell Death Differ,* 13**,** 738-47.

KISHI, K., SASAKI, T., KURODA, S., ITOH, T. & TAKAI, Y. 1993. Regulation of cytoplasmic division of Xenopus embryo by rho p21 and its inhibitory GDP/GTP exchange protein (rho GDI). *J Cell Biol,* 120**,** 1187-95.

KISHORE, R., QIN, G., LUEDEMANN, C., BORD, E., HANLEY, A., SILVER, M., GAVIN, M., YOON, Y. S., GOUKASSIAN, D. & LOSORDO, D. W. 2005. The cytoskeletal protein ezrin regulates EC proliferation and angiogenesis via TNF-alpha-induced transcriptional repression of cyclin A. *J Clin Invest,* 115**,** 1785-96.

KITADA, T., ASAKAWA, S., HATTORI, N., MATSUMINE, H., YAMAMURA, Y., MINOSHIMA, S., YOKOCHI, M., MIZUNO, Y. & SHIMIZU, N. 1998. Mutations in the parkin gene cause autosomal recessive juvenile parkinsonism. *Nature,* 392**,** 605-8.

KLAUS, A. & BIRCHMEIER, W. 2008. Wnt signalling and its impact on development and cancer. *Nat Rev Cancer,* 8**,** 387-98.

KLEIN, C. & WESTENBERGER, A. 2012. Genetics of Parkinson's disease. *Cold Spring Harb Perspect Med,* 2**,** a008888.

KLEMPNER, S. J., MYERS, A. P. & CANTLEY, L. C. 2013. What a tangled web we weave: emerging resistance mechanisms to inhibition of the phosphoinositide 3-kinase pathway. *Cancer Discov,* 3**,** 1345-54.

KRAFT, A. D., JOHNSON, D. A. & JOHNSON, J. A. 2004. Nuclear factor E2-related factor 2-dependent antioxidant response element activation by tert-butylhydroquinone and sulforaphane occurring preferentially in astrocytes conditions neurons against oxidative insult. *J Neurosci,* 24**,** 1101-12.

KRISHNAN, R., MAYS, W. & ELIJOVICH, L. 2021. Complications of Mechanical Thrombectomy in Acute Ischemic Stroke. *Neurology,* 97**,** S115-s125.

KRUPINSKI, J., ISSA, R., BUJNY, T., SLEVIN, M., KUMAR, P., KUMAR, S. & KALUZA, J. 1997. A putative role for platelet-derived growth factor in angiogenesis and neuroprotection after ischemic stroke in humans. *Stroke,* 28**,** 564-73.

KRUPINSKI, J., KALUZA, J., KUMAR, P., KUMAR, S. & WANG, J. M. 1994. Role of angiogenesis in patients with cerebral ischemic stroke. *Stroke,* 25**,** 1794-8.

KRUPINSKI, J., KUMAR, P., KUMAR, S. & KALUZA, J. 1996. Increased expression of TGF-beta 1 in brain tissue after ischemic stroke in humans. *Stroke,* 27**,** 852-7.

KUMAR, A., TAKADA, Y., BORIEK, A. M. & AGGARWAL, B. B. 2004. Nuclear factor-kappaB: its role in health and disease. *J Mol Med (Berl),* 82**,** 434-48.

LAGNA, G., HATA, A., HEMMATI-BRIVANLOU, A. & MASSAGUé, J. 1996. Partnership between DPC4 and SMAD proteins in TGF-beta signalling pathways. *Nature,* 383**,** 832-6.

LAI, M., WANG, D., LIN, Z. & ZHANG, Y. 2016. Small Molecule Copper and Its Relative Metabolites in Serum of Cerebral Ischemic Stroke Patients. *J Stroke Cerebrovasc Dis,* 25**,** 214-9.

LAI, Z. C., WEI, X., SHIMIZU, T., RAMOS, E., ROHRBAUGH, M., NIKOLAIDIS, N., HO, L. L. & LI, Y. 2005. Control of cell proliferation and apoptosis by mob as tumor suppressor, mats. *Cell,* 120**,** 675-85.

LAPCHAK, P. A. 2002. Development of thrombolytic therapy for stroke: a perspective. *Expert Opin Investig Drugs,* 11**,** 1623-32.

LAPCHAK, P. A. 2010. A critical assessment of edaravone acute ischemic stroke efficacy trials: is edaravone an effective neuroprotective therapy? *Expert Opin Pharmacother,* 11**,** 1753-63.

LAU, A., VILLENEUVE, N. F., SUN, Z., WONG, P. K. & ZHANG, D. D. 2008. Dual roles of Nrf2 in cancer. *Pharmacol Res,* 58**,** 262-70.

LEHNARDT, S., LEHMANN, S., KAUL, D., TSCHIMMEL, K., HOFFMANN, O., CHO, S., KRUEGER, C., NITSCH, R., MEISEL, A. & WEBER, J. R. 2007. Toll-like receptor 2 mediates CNS injury in focal cerebral ischemia. *J Neuroimmunol,* 190**,** 28-33.

LEKER, R. R., AHARONOWIZ, M., GREIG, N. H. & OVADIA, H. 2004. The role of p53-induced apoptosis in cerebral ischemia: effects of the p53 inhibitor pifithrin alpha. *Exp Neurol,* 187**,** 478-86.

LEMAITRE, B., NICOLAS, E., MICHAUT, L., REICHHART, J. M. & HOFFMANN, J. A. 1996. The dorsoventral regulatory gene cassette spätzle/Toll/cactus controls the potent antifungal response in Drosophila adults. *Cell,* 86**,** 973-83.

LEUNG, D. W., CACHIANES, G., KUANG, W. J., GOEDDEL, D. V. & FERRARA, N. 1989. Vascular endothelial growth factor is a secreted angiogenic mitogen. *Science,* 246**,** 1306-9.

LEUNG, S. W., LAI, J. H., WU, J. C., TSAI, Y. R., CHEN, Y. H., KANG, S. J., CHIANG, Y. H., CHANG, C. F. & CHEN, K. Y. 2020. Neuroprotective Effects of Emodin against Ischemia/Reperfusion Injury through Activating ERK-1/2 Signaling Pathway. *Int J Mol Sci,* 21.

LEVINE, A. J. 2020. p53: 800 million years of evolution and 40 years of discovery. *Nat Rev Cancer,* 20**,** 471-480.

LEVINE, A. J., MOMAND, J. & FINLAY, C. A. 1991. The p53 tumour suppressor gene. *Nature,* 351**,** 453-6.

LEZOUALC'H, F. & BEHL, C. 1998. Transcription factor NF-kappaB: friend or foe of neurons? *Mol Psychiatry,* 3**,** 15-20.

LI, C., SUI, C., WANG, W., YAN, J., DENG, N., DU, X., CHENG, F., MA, X., WANG, X. & WANG, Q. 2021a. Baicalin Attenuates Oxygen-Glucose Deprivation/Reoxygenation-Induced Injury by Modulating the BDNF-TrkB/PI3K/Akt and MAPK/Erk1/2 Signaling Axes in Neuron-Astrocyte Cocultures. *Front Pharmacol,* 12**,** 599543.

LI, D., NI, H., RUI, Q., GAO, R. & CHEN, G. 2018a. Mst1: Function and Mechanism in Brain and Myocardial Ischemia Reperfusion Injury. *Curr Neuropharmacol,* 16**,** 1358-1364.

LI, J., ZENG, Z., VIOLLET, B., RONNETT, G. V. & MCCULLOUGH, L. D. 2007. Neuroprotective effects of adenosine monophosphate-activated protein kinase inhibition and gene deletion in stroke. *Stroke,* 38**,** 2992-9.

LI, L., SUN, L., QIU, Y., ZHU, W., HU, K. & MAO, J. 2020. Protective Effect of Stachydrine Against Cerebral Ischemia-Reperfusion Injury by Reducing Inflammation and Apoptosis Through P65 and JAK2/STAT3 Signaling Pathway. *Front Pharmacol,* 11**,** 64.

LI, M., MENG, Z., YU, S., LI, J., WANG, Y., YANG, W. & WU, H. 2022. Baicalein ameliorates cerebral ischemia-reperfusion injury by inhibiting ferroptosis via regulating GPX4/ACSL4/ACSL3 axis. *Chem Biol Interact,* 366**,** 110137.

LI, N., JIANG, W., WANG, W., XIONG, R., WU, X. & GENG, Q. 2021b. Ferroptosis and its emerging roles in cardiovascular diseases. *Pharmacol Res,* 166**,** 105466.

LI, Q. Q., DING, D. H., WANG, X. Y., SUN, Y. Y. & WU, J. 2021c. Lipoxin A4 regulates microglial M1/M2 polarization after cerebral ischemia-reperfusion injury via the Notch signaling pathway. *Exp Neurol,* 339**,** 113645.

LI, Y., CHOPP, M., ZHANG, Z. G., ZALOGA, C., NIEWENHUIS, L. & GAUTAM, S. 1994. p53-immunoreactive protein and p53 mRNA expression after transient middle cerebral artery occlusion in rats. *Stroke,* 25**,** 849-55; discussion 855-6.

LI, Y., SUO, L., LIU, Y., LI, H. & XUE, W. 2017. Protective effects of ginsenoside Rg1 against oxygen-glucose-deprivation-induced apoptosis in neural stem cells. *J Neurol Sci,* 373**,** 107-112.

LI, Y., ZHU, Z. Y., LU, B. W., HUANG, T. T., ZHANG, Y. M., ZHOU, N. Y., XUAN, W., CHEN, Z. A., WEN, D. X., YU, W. F. & LI, P. Y. 2019. Rosiglitazone ameliorates tissue plasminogen activator-induced brain hemorrhage after stroke. *CNS Neurosci Ther,* 25**,** 1343-1352.

LI, Z., YAN, Z., XU, C., DONG, Y., XIONG, Y. & DAI, Y. 2018b. Acetylshikonin attenuates angiotensin II-induced proliferation and motility of human brain smooth muscle cells by inhibiting Wnt/β-catenin signaling. *Hum Cell,* 31**,** 242-250.

LIAN, I., KIM, J., OKAZAWA, H., ZHAO, J., ZHAO, B., YU, J., CHINNAIYAN, A., ISRAEL, M. A., GOLDSTEIN, L. S., ABUJAROUR, R., DING, S. & GUAN, K. L. 2010. The role of YAP transcription coactivator in regulating stem cell self-renewal and differentiation. *Genes Dev,* 24**,** 1106-18.

LIANG, C., NI, G. X., SHI, X. L., JIA, L. & WANG, Y. L. 2020. Astragaloside IV regulates the HIF/VEGF/Notch signaling pathway through miRNA-210 to promote angiogenesis after ischemic stroke. *Restor Neurol Neurosci,* 38**,** 271-282.

LIU, F., HATA, A., BAKER, J. C., DOODY, J., CáRCAMO, J., HARLAND, R. M. & MASSAGUé, J. 1996. A human Mad protein acting as a BMP-regulated transcriptional activator. *Nature,* 381**,** 620-3.

LIU, H., WU, X., LUO, J., WANG, X., GUO, H., FENG, D., ZHAO, L., BAI, H., SONG, M., LIU, X., GUO, W., LI, X., YUE, L., WANG, B. & QU, Y. 2019. Pterostilbene Attenuates Astrocytic Inflammation and Neuronal Oxidative Injury After Ischemia-Reperfusion by Inhibiting NF-κB Phosphorylation. *Front Immunol,* 10**,** 2408.

LIU, J. & WANG, L. N. 2017. Peroxisome proliferator-activated receptor gamma agonists for preventing recurrent stroke and other vascular events in people with stroke or transient ischaemic attack. *Cochrane Database Syst Rev,* 12**,** Cd010693.

LIU, Y., TANG, G., LI, Y., WANG, Y., CHEN, X., GU, X., ZHANG, Z., WANG, Y. & YANG, G. Y. 2014. Metformin attenuates blood-brain barrier disruption in mice following middle cerebral artery occlusion. *J Neuroinflammation,* 11**,** 177.

LIU, Z., ZHOU, Z., AI, P., ZHANG, C., CHEN, J. & WANG, Y. 2022. Astragaloside IV attenuates ferroptosis after subarachnoid hemorrhage via Nrf2/HO-1 signaling pathway. *Front Pharmacol,* 13**,** 924826.

LOUANDRE, C., EZZOUKHRY, Z., GODIN, C., BARBARE, J. C., MAZIèRE, J. C., CHAUFFERT, B. & GALMICHE, A. 2013. Iron-dependent cell death of hepatocellular carcinoma cells exposed to sorafenib. *Int J Cancer,* 133**,** 1732-42.

LU, W., CHEN, Z. & WEN, J. 2021. RhoA/ROCK signaling pathway and astrocytes in ischemic stroke. *Metab Brain Dis,* 36**,** 1101-1108.

LUO, Y., KUO, C. C., SHEN, H., CHOU, J., GREIG, N. H., HOFFER, B. J. & WANG, Y. 2009. Delayed treatment with a p53 inhibitor enhances recovery in stroke brain. *Ann Neurol,* 65**,** 520-30.

LV, H., LIU, X., ZENG, X., LIU, Y., ZHANG, C., ZHANG, Q. & XU, J. 2022. Comprehensive Analysis of Cuproptosis-Related Genes in Immune Infiltration and Prognosis in Melanoma. *Front Pharmacol,* 13**,** 930041.

MADAULE, P. & AXEL, R. 1985. A novel ras-related gene family. *Cell,* 41**,** 31-40.

MAO, Z., TIAN, L., LIU, J., WU, Q., WANG, N., WANG, G., WANG, Y. & SETO, S. 2022. Ligustilide ameliorates hippocampal neuronal injury after cerebral ischemia reperfusion through activating PINK1/Parkin-dependent mitophagy. *Phytomedicine,* 101**,** 154111.

MARTí-CARVAJAL, A. J., VALLI, C., MARTí-AMARISTA, C. E., SOLà, I., MARTí-FàBREGAS, J. & BONFILL COSP, X. 2020. Citicoline for treating people with acute ischemic stroke. *Cochrane Database Syst Rev,* 8**,** Cd013066.

MASTROIACOVO, F., BUSCETI, C. L., BIAGIONI, F., MOYANOVA, S. G., MEISLER, M. H., BATTAGLIA, G., CARICASOLE, A., BRUNO, V. & NICOLETTI, F. 2009. Induction of the Wnt antagonist, Dickkopf-1, contributes to the development of neuronal death in models of brain focal ischemia. *J Cereb Blood Flow Metab,* 29**,** 264-76.

MATISE, M. P. & JOYNER, A. L. 1999. Gli genes in development and cancer. *Oncogene,* 18**,** 7852-9.

MATSUDA, N., SATO, S., SHIBA, K., OKATSU, K., SAISHO, K., GAUTIER, C. A., SOU, Y. S., SAIKI, S., KAWAJIRI, S., SATO, F., KIMURA, M., KOMATSU, M., HATTORI, N. & TANAKA, K. 2010. PINK1 stabilized by mitochondrial depolarization recruits Parkin to damaged mitochondria and activates latent Parkin for mitophagy. *J Cell Biol,* 189**,** 211-21.

MATSUSHIMA, M., FUJIWARA, T., TAKAHASHI, E., MINAGUCHI, T., EGUCHI, Y., TSUJIMOTO, Y., SUZUMORI, K. & NAKAMURA, Y. 1998. Isolation, mapping, and functional analysis of a novel human cDNA (BNIP3L) encoding a protein homologous to human NIP3. *Genes Chromosomes Cancer,* 21**,** 230-5.

MCCULLOUGH, L. D., ZENG, Z., LI, H., LANDREE, L. E., MCFADDEN, J. & RONNETT, G. V. 2005. Pharmacological inhibition of AMP-activated protein kinase provides neuroprotection in stroke. *J Biol Chem,* 280**,** 20493-502.

MCMAHON, G. 2000. VEGF receptor signaling in tumor angiogenesis. *Oncologist,* 5 Suppl 1**,** 3-10.

MENGESDORF, T., JENSEN, P. H., MIES, G., AUFENBERG, C. & PASCHEN, W. 2002. Down-regulation of parkin protein in transient focal cerebral ischemia: A link between stroke and degenerative disease? *Proc Natl Acad Sci U S A,* 99**,** 15042-7.

METZ, C. W. & BRIDGES, C. B. 1917. Incompatibility of Mutant Races in Drosophila. *Proc Natl Acad Sci U S A,* 3**,** 673-8.

MI, D. H., FANG, H. J., ZHENG, G. H., LIANG, X. H., DING, Y. R., LIU, X. & LIU, L. P. 2019. DPP-4 inhibitors promote proliferation and migration of rat brain microvascular endothelial cells under hypoxic/high-glucose conditions, potentially through the SIRT1/HIF-1/VEGF pathway. *CNS Neurosci Ther,* 25**,** 323-332.

MINHAS, J. S., CHITHIRAMOHAN, T., WANG, X., BARNES, S. C., CLOUGH, R. H., KADICHEENI, M., BEISHON, L. C. & ROBINSON, T. 2022. Oral antiplatelet therapy for acute ischaemic stroke. *Cochrane Database Syst Rev,* 1**,** Cd000029.

MOI, P., CHAN, K., ASUNIS, I., CAO, A. & KAN, Y. W. 1994. Isolation of NF-E2-related factor 2 (Nrf2), a NF-E2-like basic leucine zipper transcriptional activator that binds to the tandem NF-E2/AP1 repeat of the beta-globin locus control region. *Proc Natl Acad Sci U S A,* 91**,** 9926-30.

MORII, N., SEKINE, A., OHASHI, Y., NAKAO, K., IMURA, H., FUJIWARA, M. & NARUMIYA, S. 1988. Purification and properties of the cytosolic substrate for botulinum ADP-ribosyltransferase. Identification as an Mr 22,000 guanine nucleotide-binding protein. *J Biol Chem,* 263**,** 12420-6.

MOYA, I. M. & HALDER, G. 2019. Hippo-YAP/TAZ signalling in organ regeneration and regenerative medicine. *Nat Rev Mol Cell Biol,* 20**,** 211-226.

MULLER, P. A. & VOUSDEN, K. H. 2014. Mutant p53 in cancer: new functions and therapeutic opportunities. *Cancer Cell,* 25**,** 304-17.

MUNDAY, M. R., CAMPBELL, D. G., CARLING, D. & HARDIE, D. G. 1988. Identification by amino acid sequencing of three major regulatory phosphorylation sites on rat acetyl-CoA carboxylase. *Eur J Biochem,* 175**,** 331-8.

NALAMOLU, K. R., CHALLA, S. R., FORNAL, C. A., GRUDZIEN, N. A., JORGENSON, L. C., CHOUDRY, M. M., SMITH, N. J., PALMER, C. J., PINSON, D. M., KLOPFENSTEIN, J. D. & VEERAVALLI, K. K. 2021. Attenuation of the Induction of TLRs 2 and 4 Mitigates Inflammation and Promotes Neurological Recovery After Focal Cerebral Ischemia. *Transl Stroke Res,* 12**,** 923-936.

NARUMIYA, S., SEKINE, A. & FUJIWARA, M. 1988. Substrate for botulinum ADP-ribosyltransferase, Gb, has an amino acid sequence homologous to a putative rho gene product. *J Biol Chem,* 263**,** 17255-7.

NI, J., YAO, M., WANG, L. H., YU, M., LI, R. H., ZHAO, L. H., WANG, J. C., WANG, Y. Z., WANG, X., SONG, H. Q., LUO, B. Y., WANG, J. W., HUANG, Y. N. & CUI, L. Y. 2021. Human urinary kallidinogenase in acute ischemic stroke: A single-arm, multicenter, phase IV study (RESK study). *CNS Neurosci Ther,* 27**,** 1493-1503.

NITATORI, T., SATO, N., WAGURI, S., KARASAWA, Y., ARAKI, H., SHIBANAI, K., KOMINAMI, E. & UCHIYAMA, Y. 1995. Delayed neuronal death in the CA1 pyramidal cell layer of the gerbil hippocampus following transient ischemia is apoptosis. *J Neurosci,* 15**,** 1001-11.

NOMURA, N., MIYAJIMA, N., SAZUKA, T., TANAKA, A., KAWARABAYASI, Y., SATO, S., NAGASE, T., SEKI, N., ISHIKAWA, K. & TABATA, S. 1994. Prediction of the coding sequences of unidentified human genes. I. The coding sequences of 40 new genes (KIAA0001-KIAA0040) deduced by analysis of randomly sampled cDNA clones from human immature myeloid cell line KG-1 (supplement). *DNA Res,* 1**,** 47-56.

NOORDERMEER, J., KLINGENSMITH, J., PERRIMON, N. & NUSSE, R. 1994. dishevelled and armadillo act in the wingless signalling pathway in Drosophila. *Nature,* 367**,** 80-3.

NUSSE, R. 2005. Wnt signaling in disease and in development. *Cell Res,* 15**,** 28-32.

NUSSE, R., BROWN, A., PAPKOFF, J., SCAMBLER, P., SHACKLEFORD, G., MCMAHON, A., MOON, R. & VARMUS, H. 1991. A new nomenclature for int-1 and related genes: the Wnt gene family. *Cell,* 64**,** 231.

NUSSE, R. & VARMUS, H. E. 1982. Many tumors induced by the mouse mammary tumor virus contain a provirus integrated in the same region of the host genome. *Cell,* 31**,** 99-109.

NüSSLEIN-VOLHARD, C. & WIESCHAUS, E. 1980. Mutations affecting segment number and polarity in Drosophila. *Nature,* 287**,** 795-801.

O'NEILL, L. A. & HARDIE, D. G. 2013. Metabolism of inflammation limited by AMPK and pseudo-starvation. *Nature,* 493**,** 346-55.

O'SHEA, J. J. & MURRAY, P. J. 2008. Cytokine signaling modules in inflammatory responses. *Immunity,* 28**,** 477-87.

ODA, T., ELKAHLOUN, A. G., PIKE, B. L., OKAJIMA, K., KRANTZ, I. D., GENIN, A., PICCOLI, D. A., MELTZER, P. S., SPINNER, N. B., COLLINS, F. S. & CHANDRASEKHARAPPA, S. C. 1997. Mutations in the human Jagged1 gene are responsible for Alagille syndrome. *Nat Genet,* 16**,** 235-42.

OHASHI, Y. & NARUMIYA, S. 1987. ADP-ribosylation of a Mr 21,000 membrane protein by type D botulinum toxin. *J Biol Chem,* 262**,** 1430-3.

PAHL, H. L. 1999. Activators and target genes of Rel/NF-kappaB transcription factors. *Oncogene,* 18**,** 6853-66.

PALMER, G. & GABAY, C. 2011. Interleukin-33 biology with potential insights into human diseases. *Nat Rev Rheumatol,* 7**,** 321-9.

PALMER, R. M., FERRIGE, A. G. & MONCADA, S. 1987. Nitric oxide release accounts for the biological activity of endothelium-derived relaxing factor. *Nature,* 327**,** 524-6.

PAN, B., SUN, J., LIU, Z., WANG, L., HUO, H., ZHAO, Y., TU, P., XIAO, W., ZHENG, J. & LI, J. 2021. Longxuetongluo Capsule protects against cerebral ischemia/reperfusion injury through endoplasmic reticulum stress and MAPK-mediated mechanisms. *J Adv Res,* 33**,** 215-225.

PANTALACCI, S., TAPON, N. & LéOPOLD, P. 2003. The Salvador partner Hippo promotes apoptosis and cell-cycle exit in Drosophila. *Nat Cell Biol,* 5**,** 921-7.

PEPICELLI, C. V., LEWIS, P. M. & MCMAHON, A. P. 1998. Sonic hedgehog regulates branching morphogenesis in the mammalian lung. *Curr Biol,* 8**,** 1083-6.

PHILIPS, R. L., WANG, Y., CHEON, H., KANNO, Y., GADINA, M., SARTORELLI, V., HORVATH, C. M., DARNELL, J. E., JR., STARK, G. R. & O'SHEA, J. J. 2022. The JAK-STAT pathway at 30: Much learned, much more to do. *Cell,* 185**,** 3857-3876.

PIKARSKY, E., PORAT, R. M., STEIN, I., ABRAMOVITCH, R., AMIT, S., KASEM, S., GUTKOVICH-PYEST, E., URIELI-SHOVAL, S., GALUN, E. & BEN-NERIAH, Y. 2004. NF-kappaB functions as a tumour promoter in inflammation-associated cancer. *Nature,* 431**,** 461-6.

POWERS, W. J., RABINSTEIN, A. A., ACKERSON, T., ADEOYE, O. M., BAMBAKIDIS, N. C., BECKER, K., BILLER, J., BROWN, M., DEMAERSCHALK, B. M., HOH, B., JAUCH, E. C., KIDWELL, C. S., LESLIE-MAZWI, T. M., OVBIAGELE, B., SCOTT, P. A., SHETH, K. N., SOUTHERLAND, A. M., SUMMERS, D. V. & TIRSCHWELL, D. L. 2019. Guidelines for the Early Management of Patients With Acute Ischemic Stroke: 2019 Update to the 2018 Guidelines for the Early Management of Acute Ischemic Stroke: A Guideline for Healthcare Professionals From the American Heart Association/American Stroke Association. *Stroke,* 50**,** e344-e418.

RAN, Y., SU, W., GAO, F., DING, Z., YANG, S., YE, L., CHEN, X., TIAN, G., XI, J. & LIU, Z. 2021. Curcumin Ameliorates White Matter Injury after Ischemic Stroke by Inhibiting Microglia/Macrophage Pyroptosis through NF-κB Suppression and NLRP3 Inflammasome Inhibition. *Oxid Med Cell Longev,* 2021**,** 1552127.

RAY, L. B. & STURGILL, T. W. 1988. Insulin-stimulated microtubule-associated protein kinase is phosphorylated on tyrosine and threonine in vivo. *Proc Natl Acad Sci U S A,* 85**,** 3753-7.

RIDLEY, A. J. & HALL, A. 1992. The small GTP-binding protein rho regulates the assembly of focal adhesions and actin stress fibers in response to growth factors. *Cell,* 70**,** 389-99.

RIDNOUR, L. A., CHENG, R. Y., SWITZER, C. H., HEINECKE, J. L., AMBS, S., GLYNN, S., YOUNG, H. A., TRINCHIERI, G. & WINK, D. A. 2013. Molecular pathways: toll-like receptors in the tumor microenvironment--poor prognosis or new therapeutic opportunity. *Clin Cancer Res,* 19**,** 1340-6.

RIJKEN, D. C. & COLLEN, D. 1981. Purification and characterization of the plasminogen activator secreted by human melanoma cells in culture. *J Biol Chem,* 256**,** 7035-41.

RIKITAKE, Y., KIM, H. H., HUANG, Z., SETO, M., YANO, K., ASANO, T., MOSKOWITZ, M. A. & LIAO, J. K. 2005. Inhibition of Rho kinase (ROCK) leads to increased cerebral blood flow and stroke protection. *Stroke,* 36**,** 2251-7.

ROBERTS, A. B., ANZANO, M. A., WAKEFIELD, L. M., ROCHE, N. S., STERN, D. F. & SPORN, M. B. 1985. Type beta transforming growth factor: a bifunctional regulator of cellular growth. *Proc Natl Acad Sci U S A,* 82**,** 119-23.

RONKINA, N. & GAESTEL, M. 2022. MAPK-Activated Protein Kinases: Servant or Partner? *Annu Rev Biochem,* 91**,** 505-540.

SALMINEN, A. & KAARNIRANTA, K. 2012. AMP-activated protein kinase (AMPK) controls the aging process via an integrated signaling network. *Ageing Res Rev,* 11**,** 230-41.

SANDERCOCK, P. A., COUNSELL, C. & KAMAL, A. K. 2008. Anticoagulants for acute ischaemic stroke. *Cochrane Database Syst Rev***,** Cd000024.

SARIN, A. & MARCEL, N. 2017. The NOTCH1-autophagy interaction: Regulating self-eating for survival. *Autophagy,* 13**,** 446-447.

SASAKI, H., NISHIZAKI, Y., HUI, C., NAKAFUKU, M. & KONDOH, H. 1999. Regulation of Gli2 and Gli3 activities by an amino-terminal repression domain: implication of Gli2 and Gli3 as primary mediators of Shh signaling. *Development,* 126**,** 3915-24.

SAWE, N., STEINBERG, G. & ZHAO, H. 2008. Dual roles of the MAPK/ERK1/2 cell signaling pathway after stroke. *J Neurosci Res,* 86**,** 1659-69.

SCHINDLER, C. W. 2002. Series introduction. JAK-STAT signaling in human disease. *J Clin Invest,* 109**,** 1133-7.

SCHMITZ, J., OWYANG, A., OLDHAM, E., SONG, Y., MURPHY, E., MCCLANAHAN, T. K., ZURAWSKI, G., MOSHREFI, M., QIN, J., LI, X., GORMAN, D. M., BAZAN, J. F. & KASTELEIN, R. A. 2005. IL-33, an interleukin-1-like cytokine that signals via the IL-1 receptor-related protein ST2 and induces T helper type 2-associated cytokines. *Immunity,* 23**,** 479-90.

SCHNEIDER, A., MARTIN-VILLALBA, A., WEIH, F., VOGEL, J., WIRTH, T. & SCHWANINGER, M. 1999. NF-kappaB is activated and promotes cell death in focal cerebral ischemia. *Nat Med,* 5**,** 554-9.

SEGER, R. & KREBS, E. G. 1995. The MAPK signaling cascade. *Faseb j,* 9**,** 726-35.

SEMENZA, G. L., NEJFELT, M. K., CHI, S. M. & ANTONARAKIS, S. E. 1991. Hypoxia-inducible nuclear factors bind to an enhancer element located 3' to the human erythropoietin gene. *Proc Natl Acad Sci U S A,* 88**,** 5680-4.

SEN, R. & BALTIMORE, D. 1986. Multiple nuclear factors interact with the immunoglobulin enhancer sequences. *Cell,* 46**,** 705-16.

SENGER, D. R., GALLI, S. J., DVORAK, A. M., PERRUZZI, C. A., HARVEY, V. S. & DVORAK, H. F. 1983. Tumor cells secrete a vascular permeability factor that promotes accumulation of ascites fluid. *Science,* 219**,** 983-5.

SHI, S., WANG, M., LIU, X., HAN, S. & ZHU, P. 2022. Scalp Electroacupuncture Promotes Angiogenesis after Stroke in Rats by Activation of Wnt/β-Catenin Signal Pathway. *Evid Based Complement Alternat Med,* 2022**,** 1649605.

SHIBUYA, M., HIRAI, S., SETO, M., SATOH, S. & OHTOMO, E. 2005. Effects of fasudil in acute ischemic stroke: results of a prospective placebo-controlled double-blind trial. *J Neurol Sci,* 238**,** 31-9.

SHIH, A. Y., LI, P. & MURPHY, T. H. 2005. A small-molecule-inducible Nrf2-mediated antioxidant response provides effective prophylaxis against cerebral ischemia in vivo. *J Neurosci,* 25**,** 10321-35.

SHIM, J. W. & MADSEN, J. R. 2018. VEGF Signaling in Neurological Disorders. *Int J Mol Sci,* 19.

SHIMOKAWA, H. & TAKESHITA, A. 2005. Rho-kinase is an important therapeutic target in cardiovascular medicine. *Arterioscler Thromb Vasc Biol,* 25**,** 1767-75.

SHIN, H. K., SALOMONE, S., POTTS, E. M., LEE, S. W., MILLICAN, E., NOMA, K., HUANG, P. L., BOAS, D. A., LIAO, J. K., MOSKOWITZ, M. A. & AYATA, C. 2007. Rho-kinase inhibition acutely augments blood flow in focal cerebral ischemia via endothelial mechanisms. *J Cereb Blood Flow Metab,* 27**,** 998-1009.

SHRUSTER, A., BEN-ZUR, T., MELAMED, E. & OFFEN, D. 2012. Wnt signaling enhances neurogenesis and improves neurological function after focal ischemic injury. *PLoS One,* 7**,** e40843.

SIEGFRIED, E., WILDER, E. L. & PERRIMON, N. 1994. Components of wingless signalling in Drosophila. *Nature,* 367**,** 76-80.

SIM, A. T. & HARDIE, D. G. 1988. The low activity of acetyl-CoA carboxylase in basal and glucagon-stimulated hepatocytes is due to phosphorylation by the AMP-activated protein kinase and not cyclic AMP-dependent protein kinase. *FEBS Lett,* 233**,** 294-8.

SINGH, N., MENON, B. K., DMYTRIW, A. A., REGENHARDT, R. W., HIRSCH, J. A. & GANESH, A. 2023. Replacing Alteplase with Tenecteplase: Is the Time Ripe? *J Stroke,* 25**,** 72-80.

SON, T. G., CAMANDOLA, S., ARUMUGAM, T. V., CUTLER, R. G., TELLJOHANN, R. S., MUGHAL, M. R., MOORE, T. A., LUO, W., YU, Q. S., JOHNSON, D. A., JOHNSON, J. A., GREIG, N. H. & MATTSON, M. P. 2010. Plumbagin, a novel Nrf2/ARE activator, protects against cerebral ischemia. *J Neurochem,* 112**,** 1316-26.

SPEER, R. E., KARUPPAGOUNDER, S. S., BASSO, M., SLEIMAN, S. F., KUMAR, A., BRAND, D., SMIRNOVA, N., GAZARYAN, I., KHIM, S. J. & RATAN, R. R. 2013. Hypoxia-inducible factor prolyl hydroxylases as targets for neuroprotection by "antioxidant" metal chelators: From ferroptosis to stroke. *Free Radic Biol Med,* 62**,** 26-36.

STARK, G. R. & DARNELL, J. E., JR. 2012. The JAK-STAT pathway at twenty. *Immunity,* 36**,** 503-14.

STOCKWELL, B. R., FRIEDMANN ANGELI, J. P., BAYIR, H., BUSH, A. I., CONRAD, M., DIXON, S. J., FULDA, S., GASCóN, S., HATZIOS, S. K., KAGAN, V. E., NOEL, K., JIANG, X., LINKERMANN, A., MURPHY, M. E., OVERHOLTZER, M., OYAGI, A., PAGNUSSAT, G. C., PARK, J., RAN, Q., ROSENFELD, C. S., SALNIKOW, K., TANG, D., TORTI, F. M., TORTI, S. V., TOYOKUNI, S., WOERPEL, K. A. & ZHANG, D. D. 2017. Ferroptosis: A Regulated Cell Death Nexus Linking Metabolism, Redox Biology, and Disease. *Cell,* 171**,** 273-285.

SUN, E., ZHANG, J., DENG, Y., WANG, J., WU, Q., CHEN, W., MA, X., CHEN, S., XIANG, X., CHEN, Y., WU, T., YANG, Y. & CHEN, B. 2022. Docosahexaenoic Acid Alleviates Brain Damage by Promoting Mitophagy in Mice with Ischaemic Stroke. *Oxid Med Cell Longev,* 2022**,** 3119649.

SUN, F. L., WANG, W., ZUO, W., XUE, J. L., XU, J. D., AI, H. X., ZHANG, L., WANG, X. M. & JI, X. M. 2014. Promoting neurogenesis via Wnt/β-catenin signaling pathway accounts for the neurorestorative effects of morroniside against cerebral ischemia injury. *Eur J Pharmacol,* 738**,** 214-21.

SUN, L., ZHANG, H., WANG, W., CHEN, Z., WANG, S., LI, J., LI, G., GAO, C. & SUN, X. 2020. Astragaloside IV Exerts Cognitive Benefits and Promotes Hippocampal Neurogenesis in Stroke Mice by Downregulating Interleukin-17 Expression via Wnt Pathway. *Front Pharmacol,* 11**,** 421.

SUZUKI, S., TANAKA, K., NOGAWA, S., DEMBO, T., KOSAKAI, A. & FUKUUCHI, Y. 2001. Phosphorylation of signal transducer and activator of transcription-3 (Stat3) after focal cerebral ischemia in rats. *Exp Neurol,* 170**,** 63-71.

SZABO, C. 2017. Hydrogen sulfide, an enhancer of vascular nitric oxide signaling: mechanisms and implications. *Am J Physiol Cell Physiol,* 312**,** C3-C15.

TACHIBANA, E., HARADA, T., SHIBUYA, M., SAITO, K., TAKAYASU, M., SUZUKI, Y. & YOSHIDA, J. 1999. Intra-arterial infusion of fasudil hydrochloride for treating vasospasm following subarachnoid haemorrhage. *Acta Neurochir (Wien),* 141**,** 13-9.

TAM, J. P., MARQUARDT, H., ROSBERGER, D. F., WONG, T. W. & TODARO, G. J. 1984. Synthesis of biologically active rat transforming growth factor I. *Nature,* 309**,** 376-8.

TAN, J., LUO, J., MENG, C., JIANG, N., CAO, J. & ZHAO, J. 2021. Syringin exerts neuroprotective effects in a rat model of cerebral ischemia through the FOXO3a/NF-κB pathway. *Int Immunopharmacol,* 90**,** 107268.

TANG, C., HONG, J., HU, C., HUANG, C., GAO, J., HUANG, J., WANG, D., GENG, Q. & DONG, Y. 2021. Palmatine Protects against Cerebral Ischemia/Reperfusion Injury by Activation of the AMPK/Nrf2 Pathway. *Oxid Med Cell Longev,* 2021**,** 6660193.

TANG, D., CHEN, X. & KROEMER, G. 2022. Cuproptosis: a copper-triggered modality of mitochondrial cell death. *Cell Res,* 32**,** 417-418.

TANG, J., HU, Z., TAN, J., YANG, S. & ZENG, L. 2016. Parkin Protects against Oxygen-Glucose Deprivation/Reperfusion Insult by Promoting Drp1 Degradation. *Oxid Med Cell Longev,* 2016**,** 8474303.

TANG, S. C., YEH, S. J., LI, Y. I., WANG, Y. C., BAIK, S. H., SANTRO, T., WIDIAPRADJA, A., MANZANERO, S., SOBEY, C. G., JO, D. G., ARUMUGAM, T. V. & JENG, J. S. 2013. Evidence for a detrimental role of TLR8 in ischemic stroke. *Exp Neurol,* 250**,** 341-7.

TAO, G., KAHR, P. C., MORIKAWA, Y., ZHANG, M., RAHMANI, M., HEALLEN, T. R., LI, L., SUN, Z., OLSON, E. N., AMENDT, B. A. & MARTIN, J. F. 2016. Pitx2 promotes heart repair by activating the antioxidant response after cardiac injury. *Nature,* 534**,** 119-23.

TAPON, N., HARVEY, K. F., BELL, D. W., WAHRER, D. C., SCHIRIPO, T. A., HABER, D. & HARIHARAN, I. K. 2002. salvador Promotes both cell cycle exit and apoptosis in Drosophila and is mutated in human cancer cell lines. *Cell,* 110**,** 467-78.

TAYLOR, J. M., COHEN, S. & MITCHELL, W. M. 1970. Epidermal growth factor: high and low molecular weight forms. *Proc Natl Acad Sci U S A,* 67**,** 164-71.

THOMPSON, C. B. 2016. Into Thin Air: How We Sense and Respond to Hypoxia. *Cell,* 167**,** 9-11.

TOMAS, A., FUTTER, C. E. & EDEN, E. R. 2014. EGF receptor trafficking: consequences for signaling and cancer. *Trends Cell Biol,* 24**,** 26-34.

TSIVGOULIS, G., KADLECOVá, P., KOBAYASHI, A., CZLONKOWSKA, A., BROZMAN, M., ŠVIGELJ, V., CSIBA, L., KõRV, J., DEMARIN, V., VILIONSKIS, A., JATUZIS, D., KATSANOS, A. H., RUDOLF, J., KRESPI, Y. & MIKULIK, R. 2015. Safety of Statin Pretreatment in Intravenous Thrombolysis for Acute Ischemic Stroke. *Stroke,* 46**,** 2681-4.

TUO, Q. Z., LIU, Y., XIANG, Z., YAN, H. F., ZOU, T., SHU, Y., DING, X. L., ZOU, J. J., XU, S., TANG, F., GONG, Y. Q., LI, X. L., GUO, Y. J., ZHENG, Z. Y., DENG, A. P., YANG, Z. Z., LI, W. J., ZHANG, S. T., AYTON, S., BUSH, A. I., XU, H., DAI, L., DONG, B. & LEI, P. 2022. Thrombin induces ACSL4-dependent ferroptosis during cerebral ischemia/reperfusion. *Signal Transduct Target Ther,* 7**,** 59.

UDAN, R. S., KANGO-SINGH, M., NOLO, R., TAO, C. & HALDER, G. 2003. Hippo promotes proliferation arrest and apoptosis in the Salvador/Warts pathway. *Nat Cell Biol,* 5**,** 914-20.

UEHATA, M., ISHIZAKI, T., SATOH, H., ONO, T., KAWAHARA, T., MORISHITA, T., TAMAKAWA, H., YAMAGAMI, K., INUI, J., MAEKAWA, M. & NARUMIYA, S. 1997. Calcium sensitization of smooth muscle mediated by a Rho-associated protein kinase in hypertension. *Nature,* 389**,** 990-4.

UGOLINI, F., CHARAFE-JAUFFRET, E., BARDOU, V. J., GENEIX, J., ADéLAïDE, J., LABAT-MOLEUR, F., PENAULT-LLORCA, F., LONGY, M., JACQUEMIER, J., BIRNBAUM, D. & PéBUSQUE, M. J. 2001. WNT pathway and mammary carcinogenesis: loss of expression of candidate tumor suppressor gene SFRP1 in most invasive carcinomas except of the medullary type. *Oncogene,* 20**,** 5810-7.

VALENTE, E. M., ABOU-SLEIMAN, P. M., CAPUTO, V., MUQIT, M. M., HARVEY, K., GISPERT, S., ALI, Z., DEL TURCO, D., BENTIVOGLIO, A. R., HEALY, D. G., ALBANESE, A., NUSSBAUM, R., GONZáLEZ-MALDONADO, R., DELLER, T., SALVI, S., CORTELLI, P., GILKS, W. P., LATCHMAN, D. S., HARVEY, R. J., DALLAPICCOLA, B., AUBURGER, G. & WOOD, N. W. 2004. Hereditary early-onset Parkinson's disease caused by mutations in PINK1. *Science,* 304**,** 1158-60.

VAN BRUGGEN, N., THIBODEAUX, H., PALMER, J. T., LEE, W. P., FU, L., CAIRNS, B., TUMAS, D., GERLAI, R., WILLIAMS, S. P., VAN LOOKEREN CAMPAGNE, M. & FERRARA, N. 1999. VEGF antagonism reduces edema formation and tissue damage after ischemia/reperfusion injury in the mouse brain. *J Clin Invest,* 104**,** 1613-20.

VAN NIEUW AMERONGEN, G. P., KOOLWIJK, P., VERSTEILEN, A. & VAN HINSBERGH, V. W. 2003. Involvement of RhoA/Rho kinase signaling in VEGF-induced endothelial cell migration and angiogenesis in vitro. *Arterioscler Thromb Vasc Biol,* 23**,** 211-7.

VARELAS, X., SAKUMA, R., SAMAVARCHI-TEHRANI, P., PEERANI, R., RAO, B. M., DEMBOWY, J., YAFFE, M. B., ZANDSTRA, P. W. & WRANA, J. L. 2008. TAZ controls Smad nucleocytoplasmic shuttling and regulates human embryonic stem-cell self-renewal. *Nat Cell Biol,* 10**,** 837-48.

VEIKKOLA, T. & ALITALO, K. 1999. VEGFs, receptors and angiogenesis. *Semin Cancer Biol,* 9**,** 211-20.

VOUSDEN, K. H. & LU, X. 2002. Live or let die: the cell's response to p53. *Nat Rev Cancer,* 2**,** 594-604.

WABNITZ, A. & CHIMOWITZ, M. 2017. Angioplasty, Stenting and Other Potential Treatments of Atherosclerotic Stenosis of the Intracranial Arteries: Past, Present and Future. *J Stroke,* 19**,** 271-276.

WAGNER, E. F. & NEBREDA, A. R. 2009. Signal integration by JNK and p38 MAPK pathways in cancer development. *Nat Rev Cancer,* 9**,** 537-49.

WALDNER, M. J. & NEURATH, M. F. 2012. Targeting the VEGF signaling pathway in cancer therapy. *Expert Opin Ther Targets,* 16**,** 5-13.

WAN, D., ZHOU, Y., WANG, K., HOU, Y., HOU, R. & YE, X. 2016. Resveratrol provides neuroprotection by inhibiting phosphodiesterases and regulating the cAMP/AMPK/SIRT1 pathway after stroke in rats. *Brain Res Bull,* 121**,** 255-62.

WANG, G., CHEN, Z., SONG, Y., WU, H., CHEN, M., LAI, S. & WU, X. 2022a. Xueshuantong injection alleviates cerebral microcirculation disorder in middle cerebral artery occlusion/reperfusion rats by suppressing inflammation via JNK mediated JAK2/STAT3 and NF-κB signaling pathways. *J Ethnopharmacol,* 298**,** 115592.

WANG, H., CHEN, S., ZHANG, Y., XU, H. & SUN, H. 2019. Electroacupuncture ameliorates neuronal injury by Pink1/Parkin-mediated mitophagy clearance in cerebral ischemia-reperfusion. *Nitric Oxide,* 91**,** 23-34.

WANG, H., GUO, M., WEI, H. & CHEN, Y. 2023. Targeting p53 pathways: mechanisms, structures, and advances in therapy. *Signal Transduct Target Ther,* 8**,** 92.

WANG, H., XU, X., YIN, Y., YU, S., REN, H., XUE, Q. & XU, X. 2020. Catalpol protects vascular structure and promotes angiogenesis in cerebral ischemic rats by targeting HIF-1α/VEGF. *Phytomedicine,* 78**,** 153300.

WANG, H., YE, K., LI, D., LIU, Y. & WANG, D. 2022b. DL-3-n-butylphthalide for acute ischemic stroke: An updated systematic review and meta-analysis of randomized controlled trials. *Front Pharmacol,* 13**,** 963118.

WANG, L. P., PAN, J., LI, Y., GENG, J., LIU, C., ZHANG, L. Y., ZHOU, P., TANG, Y. H., WANG, Y., ZHANG, Z. & YANG, G. Y. 2022c. Oligodendrocyte precursor cell transplantation promotes angiogenesis and remyelination via Wnt/β-catenin pathway in a mouse model of middle cerebral artery occlusion. *J Cereb Blood Flow Metab,* 42**,** 757-770.

WANG, P. R., WANG, J. S., ZHANG, C., SONG, X. F., TIAN, N. & KONG, L. Y. 2013. Huang-Lian-Jie-Du-Decotion induced protective autophagy against the injury of cerebral ischemia/reperfusion via MAPK-mTOR signaling pathway. *J Ethnopharmacol,* 149**,** 270-80.

WANG, S., SHI, X., LI, H., PANG, P., PEI, L., SHEN, H. & LU, Y. 2017. DAPK1 Signaling Pathways in Stroke: from Mechanisms to Therapies. *Mol Neurobiol,* 54**,** 4716-4722.

WANG, S., YIN, J., GE, M., DAI, Z., LI, Y., SI, J., MA, K., LI, L. & YAO, S. 2016a. Transforming growth-beta 1 contributes to isoflurane postconditioning against cerebral ischemia-reperfusion injury by regulating the c-Jun N-terminal kinase signaling pathway. *Biomed Pharmacother,* 78**,** 280-290.

WANG, W., LI, M., WANG, Y., LI, Q., DENG, G., WAN, J., YANG, Q., CHEN, Q. & WANG, J. 2016b. GSK-3β inhibitor TWS119 attenuates rtPA-induced hemorrhagic transformation and activates the Wnt/β-catenin signaling pathway after acute ischemic stroke in rats. *Mol Neurobiol,* 53**,** 7028-7036.

WANG, X., LI, X., XU, Y., LI, R., YANG, Q., ZHAO, Y., WANG, F., SHENG, B., WANG, R., CHEN, S., WANG, L., SHEN, L., HOU, X., CUI, Y., WANG, D., PENG, B., ANDERSON, C. S. & CHEN, H. 2021. Effectiveness of intravenous r-tPA versus UK for acute ischaemic stroke: a nationwide prospective Chinese registry study. *Stroke Vasc Neurol,* 6**,** 603-609.

WANG, X., PEI, L., YAN, H., WANG, Z., WEI, N., WANG, S., YANG, X., TIAN, Q. & LU, Y. 2014. Intervention of death-associated protein kinase 1-p53 interaction exerts the therapeutic effects against stroke. *Stroke,* 45**,** 3089-91.

WANG, Y., HONG, F. & YANG, S. 2022d. Roles of Nitric Oxide in Brain Ischemia and Reperfusion. *Int J Mol Sci,* 23.

WARDLAW, J. M., MURRAY, V., BERGE, E. & DEL ZOPPO, G. J. 2014. Thrombolysis for acute ischaemic stroke. *Cochrane Database Syst Rev,* 2014**,** Cd000213.

WEI, R., SONG, L., MIAO, Z., LIU, K., HAN, G., ZHANG, H., MA, D., HUANG, J., TIAN, H., XIAO, B. & MA, C. 2022. Hydroxysafflor Yellow A Exerts Neuroprotective Effects via HIF-1α/BNIP3 Pathway to Activate Neuronal Autophagy after OGD/R. *Cells,* 11.

WEI, Z., CHIGURUPATI, S., ARUMUGAM, T. V., JO, D. G., LI, H. & CHAN, S. L. 2011. Notch activation enhances the microglia-mediated inflammatory response associated with focal cerebral ischemia. *Stroke,* 42**,** 2589-94.

WEILAND, A., WANG, Y., WU, W., LAN, X., HAN, X., LI, Q. & WANG, J. 2019. Ferroptosis and Its Role in Diverse Brain Diseases. *Mol Neurobiol,* 56**,** 4880-4893.

WELLS, A. 1999. EGF receptor. *Int J Biochem Cell Biol,* 31**,** 637-43.

WHARTON, K. A., JOHANSEN, K. M., XU, T. & ARTAVANIS-TSAKONAS, S. 1985. Nucleotide sequence from the neurogenic locus notch implies a gene product that shares homology with proteins containing EGF-like repeats. *Cell,* 43**,** 567-81.

WHITMAN, M., DOWNES, C. P., KEELER, M., KELLER, T. & CANTLEY, L. 1988. Type I phosphatidylinositol kinase makes a novel inositol phospholipid, phosphatidylinositol-3-phosphate. *Nature,* 332**,** 644-6.

WILLMOT, M. R. & BATH, P. M. 2003. The potential of nitric oxide therapeutics in stroke. *Expert Opin Investig Drugs,* 12**,** 455-70.

WU, F., ZHANG, Y., SUN, B., MCMAHON, A. P. & WANG, Y. 2017a. Hedgehog Signaling: From Basic Biology to Cancer Therapy. *Cell Chem Biol,* 24**,** 252-280.

WU, L., WANG, H. M., LI, J. L., FENG, H. X., ZHAO, W. M. & ZHANG, H. Y. 2017b. Dual anti-ischemic effects of rosmarinic acid n-butyl ester via alleviation of DAPK-p53-mediated neuronal damage and microglial inflammation. *Acta Pharmacol Sin,* 38**,** 459-468.

WU, S., HUANG, J., DONG, J. & PAN, D. 2003. hippo encodes a Ste-20 family protein kinase that restricts cell proliferation and promotes apoptosis in conjunction with salvador and warts. *Cell,* 114**,** 445-56.

WU, X., LI, X., LIU, Y., YUAN, N., LI, C., KANG, Z., ZHANG, X., XIA, Y., HAO, Y. & TAN, Y. 2018. Hydrogen exerts neuroprotective effects on OGD/R damaged neurons in rat hippocampal by protecting mitochondrial function via regulating mitophagy mediated by PINK1/Parkin signaling pathway. *Brain Res,* 1698**,** 89-98.

XIAN, M., CAI, J., ZHENG, K., LIU, Q., LIU, Y., LIN, H., LIANG, S. & WANG, S. 2021. Aloe-emodin prevents nerve injury and neuroinflammation caused by ischemic stroke via the PI3K/AKT/mTOR and NF-κB pathway. *Food Funct,* 12**,** 8056-8067.

XIAO, G., LYU, M., LI, Z., CAO, L., LIU, X., WANG, Y., HE, S., CHEN, Z., DU, H., FENG, Y., WANG, J. & ZHU, Y. 2021. Restoration of early deficiency of axonal guidance signaling by guanxinning injection as a novel therapeutic option for acute ischemic stroke. *Pharmacol Res,* 165**,** 105460.

XIN, N., YANG, F. J., LI, Y., LI, Y. J., DAI, R. J., MENG, W. W., CHEN, Y. & DENG, Y. L. 2013. Dragon's blood dropping pills have protective effects on focal cerebral ischemia rats model. *Phytomedicine,* 21**,** 68-74.

XU, D., HOU, K., LI, F., CHEN, S., FANG, W. & LI, Y. 2019. XQ-1H alleviates cerebral ischemia in mice through inhibition of apoptosis and promotion of neurogenesis in a Wnt/β-catenin signaling dependent way. *Life Sci,* 235**,** 116844.

XU, F., NA, L., LI, Y. & CHEN, L. 2020. Roles of the PI3K/AKT/mTOR signalling pathways in neurodegenerative diseases and tumours. *Cell Biosci,* 10**,** 54.

XU, T., WANG, W., ZHANG, S., STEWART, R. A. & YU, W. 1995. Identifying tumor suppressors in genetic mosaics: the Drosophila lats gene encodes a putative protein kinase. *Development,* 121**,** 1053-63.

XU, Y., ZHANG, G., KANG, Z., XU, Y., JIANG, W. & ZHANG, S. 2016. Cornin increases angiogenesis and improves functional recovery after stroke via the Ang1/Tie2 axis and the Wnt/β-catenin pathway. *Arch Pharm Res,* 39**,** 133-42.

XU, Z. & FORD, B. D. 2005. Upregulation of erbB receptors in rat brain after middle cerebral arterial occlusion. *Neurosci Lett,* 375**,** 181-6.

YAGITA, Y., KITAGAWA, K., SASAKI, T., TERASAKI, Y., TODO, K., OMURA-MATSUOKA, E., KAIBUCHI, K. & HORI, M. 2007. Rho-kinase activation in endothelial cells contributes to expansion of infarction after focal cerebral ischemia. *J Neurosci Res,* 85**,** 2460-9.

YAMAGUCHI, A., TANIGUCHI, M., HORI, O., OGAWA, S., TOJO, N., MATSUOKA, N., MIYAKE, S., KASAI, K., SUGIMOTO, H., TAMATANI, M., YAMASHITA, T. & TOHYAMA, M. 2002. Peg3/Pw1 is involved in p53-mediated cell death pathway in brain ischemia/hypoxia. *J Biol Chem,* 277**,** 623-9.

YANG, B., LI, Y., MA, Y., ZHANG, X., YANG, L., SHEN, X., ZHANG, J. & JING, L. 2021. Selenium attenuates ischemia/reperfusion injury‑induced damage to the blood‑brain barrier in hyperglycemia through PI3K/AKT/mTOR pathway‑mediated autophagy inhibition. *Int J Mol Med,* 48.

YANG, L., TAO, L. Y. & CHEN, X. P. 2007. Roles of NF-kappaB in central nervous system damage and repair. *Neurosci Bull,* 23**,** 307-13.

YANG, S., WANG, H., YANG, Y., WANG, R., WANG, Y., WU, C. & DU, G. 2019. Baicalein administered in the subacute phase ameliorates ischemia-reperfusion-induced brain injury by reducing neuroinflammation and neuronal damage. *Biomed Pharmacother,* 117**,** 109102.

YANG, W. S., SRIRAMARATNAM, R., WELSCH, M. E., SHIMADA, K., SKOUTA, R., VISWANATHAN, V. S., CHEAH, J. H., CLEMONS, P. A., SHAMJI, A. F., CLISH, C. B., BROWN, L. M., GIROTTI, A. W., CORNISH, V. W., SCHREIBER, S. L. & STOCKWELL, B. R. 2014. Regulation of ferroptotic cancer cell death by GPX4. *Cell,* 156**,** 317-331.

YANG, Y., HE, B., ZHANG, X., YANG, R., XIA, X., CHEN, L., LI, R., SHEN, Z. & CHEN, P. 2022. Geraniin Protects against Cerebral Ischemia/Reperfusion Injury by Suppressing Oxidative Stress and Neuronal Apoptosis via Regulation of the Nrf2/HO-1 Pathway. *Oxid Med Cell Longev,* 2022**,** 2152746.

YEH, S. H., OU, L. C., GEAN, P. W., HUNG, J. J. & CHANG, W. C. 2011. Selective inhibition of early--but not late--expressed HIF-1α is neuroprotective in rats after focal ischemic brain damage. *Brain Pathol,* 21**,** 249-62.

YOCHEM, J., WESTON, K. & GREENWALD, I. 1988. The Caenorhabditis elegans lin-12 gene encodes a transmembrane protein with overall similarity to Drosophila Notch. *Nature,* 335**,** 547-50.

YONISH-ROUACH, E., RESNITZKY, D., LOTEM, J., SACHS, L., KIMCHI, A. & OREN, M. 1991. Wild-type p53 induces apoptosis of myeloid leukaemic cells that is inhibited by interleukin-6. *Nature,* 352**,** 345-7.

YU, J., WANG, W. N., MATEI, N., LI, X., PANG, J. W., MO, J., CHEN, S. P., TANG, J. P., YAN, M. & ZHANG, J. H. 2020a. Ezetimibe Attenuates Oxidative Stress and Neuroinflammation via the AMPK/Nrf2/TXNIP Pathway after MCAO in Rats. *Oxid Med Cell Longev,* 2020**,** 4717258.

YU, L., LIU, Z., HE, W., CHEN, H., LAI, Z., DUAN, Y., CAO, X., TAO, J., XU, C., ZHANG, Q., ZHAO, Z. & ZHANG, J. 2020b. Hydroxysafflor Yellow A Confers Neuroprotection from Focal Cerebral Ischemia by Modulating the Crosstalk Between JAK2/STAT3 and SOCS3 Signaling Pathways. *Cell Mol Neurobiol,* 40**,** 1271-1281.

YU, P., WANG, L., TANG, F., GUO, S., LIAO, H., FAN, C. & YANG, Q. 2021. Resveratrol-mediated neurorestoration after cerebral ischemic injury - Sonic Hedgehog signaling pathway. *Life Sci,* 280**,** 119715.

YU, P., WANG, L., TANG, F., ZENG, L., ZHOU, L., SONG, X., JIA, W., CHEN, J. & YANG, Q. 2017. Resveratrol Pretreatment Decreases Ischemic Injury and Improves Neurological Function Via Sonic Hedgehog Signaling After Stroke in Rats. *Mol Neurobiol,* 54**,** 212-226.

YU, Y., LI, J., ZHOU, H., XIONG, Y., WEN, Y. & LI, H. 2018. Functional importance of the TGF-β1/Smad3 signaling pathway in oxygen-glucose-deprived (OGD) microglia and rats with cerebral ischemia. *Int J Biol Macromol,* 116**,** 537-544.

YUAN, Y., ZHAI, Y., CHEN, J., XU, X. & WANG, H. 2021. Kaempferol Ameliorates Oxygen-Glucose Deprivation/Reoxygenation-Induced Neuronal Ferroptosis by Activating Nrf2/SLC7A11/GPX4 Axis. *Biomolecules,* 11.

YUAN, Y., ZHENG, Y., ZHANG, X., CHEN, Y., WU, X., WU, J., SHEN, Z., JIANG, L., WANG, L., YANG, W., LUO, J., QIN, Z., HU, W. & CHEN, Z. 2017. BNIP3L/NIX-mediated mitophagy protects against ischemic brain injury independent of PARK2. *Autophagy,* 13**,** 1754-1766.

ZACHAREK, A., CHEN, J., CUI, X., YANG, Y. & CHOPP, M. 2009. Simvastatin increases notch signaling activity and promotes arteriogenesis after stroke. *Stroke,* 40**,** 254-60.

ZANCONATO, F., CORDENONSI, M. & PICCOLO, S. 2016. YAP/TAZ at the Roots of Cancer. *Cancer Cell,* 29**,** 783-803.

ZANIN-ZHOROV, A., WEISS, J. M., NYUYDZEFE, M. S., CHEN, W., SCHER, J. U., MO, R., DEPOIL, D., RAO, N., LIU, B., WEI, J., LUCAS, S., KOSLOW, M., ROCHE, M., SCHUELLER, O., WEISS, S., POYUROVSKY, M. V., TONRA, J., HIPPEN, K. L., DUSTIN, M. L., BLAZAR, B. R., LIU, C. J. & WAKSAL, S. D. 2014. Selective oral ROCK2 inhibitor down-regulates IL-21 and IL-17 secretion in human T cells via STAT3-dependent mechanism. *Proc Natl Acad Sci U S A,* 111**,** 16814-9.

ZENG, J., ZHENG, S., CHEN, Y., QU, Y., XIE, J., HONG, E., LV, H., DING, R., FENG, L. & XIE, Z. 2021. Puerarin attenuates intracerebral hemorrhage-induced early brain injury possibly by PI3K/Akt signal activation-mediated suppression of NF-κB pathway. *J Cell Mol Med,* 25**,** 7809-7824.

ZERLIN, M., JULIUS, M. A. & KITAJEWSKI, J. 2008. Wnt/Frizzled signaling in angiogenesis. *Angiogenesis,* 11**,** 63-9.

ZHAN, S., LIANG, J., LIN, H., CAI, J., YANG, X., WU, H., WEI, J., WANG, S. & XIAN, M. 2022. SATB1/SLC7A11/HO-1 Axis Ameliorates Ferroptosis in Neuron Cells After Ischemic Stroke by Danhong Injection. *Mol Neurobiol*.

ZHANG, C., TAO, W., LIU, M. & WANG, D. 2012. Efficacy and safety of human urinary kallidinogenase injection for acute ischemic stroke: a systematic review. *J Evid Based Med,* 5**,** 31-9.

ZHANG, D. D. 2006. Mechanistic studies of the Nrf2-Keap1 signaling pathway. *Drug Metab Rev,* 38**,** 769-89.

ZHANG, W., SONG, J., LI, W., KONG, D., LIANG, Y., ZHAO, X. & DU, G. 2020. Salvianolic Acid D Alleviates Cerebral Ischemia-Reperfusion Injury by Suppressing the Cytoplasmic Translocation and Release of HMGB1-Triggered NF-κB Activation to Inhibit Inflammatory Response. *Mediators Inflamm,* 2020**,** 9049614.

ZHANG, W., SONG, J. K., YAN, R., LI, L., XIAO, Z. Y., ZHOU, W. X., WANG, Z. Z., XIAO, W. & DU, G. H. 2018. Diterpene ginkgolides protect against cerebral ischemia/reperfusion damage in rats by activating Nrf2 and CREB through PI3K/Akt signaling. *Acta Pharmacol Sin,* 39**,** 1259-1272.

ZHANG, Y., JANSSENS, S. P., WINGLER, K., SCHMIDT, H. H. & MOENS, A. L. 2011. Modulating endothelial nitric oxide synthase: a new cardiovascular therapeutic strategy. *Am J Physiol Heart Circ Physiol,* 301**,** H634-46.

ZHANG, Y., LIU, D., HU, H., ZHANG, P., XIE, R. & CUI, W. 2019. HIF-1α/BNIP3 signaling pathway-induced-autophagy plays protective role during myocardial ischemia-reperfusion injury. *Biomed Pharmacother,* 120**,** 109464.

ZHANG, Y. & MIAO, J. M. 2018. Ginkgolide K promotes astrocyte proliferation and migration after oxygen-glucose deprivation via inducing protective autophagy through the AMPK/mTOR/ULK1 signaling pathway. *Eur J Pharmacol,* 832**,** 96-103.

ZHANG, Z., YANG, X., ZHANG, S., MA, X. & KONG, J. 2007. BNIP3 upregulation and EndoG translocation in delayed neuronal death in stroke and in hypoxia. *Stroke,* 38**,** 1606-13.

ZHANG, Z. G., ZHANG, L., JIANG, Q., ZHANG, R., DAVIES, K., POWERS, C., BRUGGEN, N. & CHOPP, M. 2000. VEGF enhances angiogenesis and promotes blood-brain barrier leakage in the ischemic brain. *J Clin Invest,* 106**,** 829-38.

ZHAO, S., YIN, J., ZHOU, L., YAN, F., HE, Q., HUANG, L., PENG, S., JIA, J., CHENG, J., CHEN, H., TAO, W., JI, X., XU, Y. & YUAN, Z. 2016. Hippo/MST1 signaling mediates microglial activation following acute cerebral ischemia-reperfusion injury. *Brain Behav Immun,* 55**,** 236-248.

ZHAO, X., SUN, G., ZHANG, J., TING, S. M., GONZALES, N. & ARONOWSKI, J. 2015. Dimethyl Fumarate Protects Brain From Damage Produced by Intracerebral Hemorrhage by Mechanism Involving Nrf2. *Stroke,* 46**,** 1923-8.

ZHAO, Y., CHEN, F., CHEN, S., LIU, X., CUI, M. & DONG, Q. 2013. The Parkinson's disease-associated gene PINK1 protects neurons from ischemic damage by decreasing mitochondrial translocation of the fission promoter Drp1. *J Neurochem,* 127**,** 711-22.

ZHAO, Y., QIAN, Y., SUN, Z., SHEN, X., CAI, Y., LI, L. & WANG, Z. 2021. Role of PI3K in the Progression and Regression of Atherosclerosis. *Front Pharmacol,* 12**,** 632378.

ZHONG, W. J., YANG, X. S., ZHOU, H., XIE, B. R., LIU, W. W. & LI, Y. 2022. Role of Mitophagy in the Pathogenesis of Stroke: From Mechanism to Therapy. *Oxid Med Cell Longev,* 2022**,** 6232902.

ZHOU, G., MYERS, R., LI, Y., CHEN, Y., SHEN, X., FENYK-MELODY, J., WU, M., VENTRE, J., DOEBBER, T., FUJII, N., MUSI, N., HIRSHMAN, M. F., GOODYEAR, L. J. & MOLLER, D. E. 2001. Role of AMP-activated protein kinase in mechanism of metformin action. *J Clin Invest,* 108**,** 1167-74.

ZHOU, K., CHEN, J., WU, J., WU, Q., JIA, C., XU, Y. X. Z., CHEN, L., TU, W., YANG, G., KONG, J., KOU, J. & JIANG, S. 2019. Atractylenolide III ameliorates cerebral ischemic injury and neuroinflammation associated with inhibiting JAK2/STAT3/Drp1-dependent mitochondrial fission in microglia. *Phytomedicine,* 59**,** 152922.

ZHOU, Z. Q., LI, Y. L., AO, Z. B., WEN, Z. L., CHEN, Q. W., HUANG, Z. G., XIAO, B. & YAN, X. H. 2017. Baicalin protects neonatal rat brains against hypoxic-ischemic injury by upregulating glutamate transporter 1 via the phosphoinositide 3-kinase/protein kinase B signaling pathway. *Neural Regen Res,* 12**,** 1625-1631.

ZHOU, Z. X., CUI, Q., ZHANG, Y. M., YANG, J. X., XIANG, W. J., TIAN, N., JIANG, Y. L., CHEN, M. L., YANG, B., LI, Q. H. & LIAO, R. J. 2023. Withaferin A inhibits ferroptosis and protects against intracerebral hemorrhage. *Neural Regen Res,* 18**,** 1308-1315.

ZHU, H., JIAN, Z., ZHONG, Y., YE, Y., ZHANG, Y., HU, X., PU, B., GU, L. & XIONG, X. 2021. Janus Kinase Inhibition Ameliorates Ischemic Stroke Injury and Neuroinflammation Through Reducing NLRP3 Inflammasome Activation via JAK2/STAT3 Pathway Inhibition. *Front Immunol,* 12**,** 714943.

ZHU, J., CAO, D., GUO, C., LIU, M., TAO, Y., ZHOU, J., WANG, F., ZHAO, Y., WEI, J., ZHANG, Y., FANG, W. & LI, Y. 2019. Berberine Facilitates Angiogenesis Against Ischemic Stroke Through Modulating Microglial Polarization via AMPK Signaling. *Cell Mol Neurobiol,* 39**,** 751-768.

ZILLE, M., KARUPPAGOUNDER, S. S., CHEN, Y., GOUGH, P. J., BERTIN, J., FINGER, J., MILNER, T. A., JONAS, E. A. & RATAN, R. R. 2017. Neuronal Death After Hemorrhagic Stroke In Vitro and In Vivo Shares Features of Ferroptosis and Necroptosis. *Stroke,* 48**,** 1033-1043.

ZOLEZZI, J. M., SANTOS, M. J., BASTíAS-CANDIA, S., PINTO, C., GODOY, J. A. & INESTROSA, N. C. 2017. PPARs in the central nervous system: roles in neurodegeneration and neuroinflammation. *Biol Rev Camb Philos Soc,* 92**,** 2046-2069.
